# Supplementary material for: Interactions between physicians and the pharmaceutical industry generally and sales representatives specifically and their association with physicians’ attitudes and prescribing habits: a systematic review
Source: BMJ Open. 2017 Sep 27;7(9):e016408. doi: 10.1136/bmjopen-2017-016408 (PMC5623540; doi:10.1136/bmjopen-2017-016408)
Supplement: Supplementary material 2 [file bmjopen-2017-016408supp002.pdf]

Search strategy for PubMed search engine of Medline

In an attempt to find all related literature on the topic, studies related to physician-pharmaceutical representative interactions that affect the prescribing behavior of the physicians were identified through computerized searches using, but not limited to, the following subject headings and text words in PubMed from 1992 to 2016.

1. Physician interactions with pharmaceutical industry
2. Physician attitude towards pharmaceutical representatives
3. Behavior of physicians towards pharmaceutical representatives
4. Gifts AND physician AND pharmaceutical representatives
5. Honoraria AND physician AND pharmaceutical representatives
6. Continuing medical education AND physician AND pharmaceutical representatives
7. Research funding AND physician AND pharmaceutical representatives
8. Conference travel AND physician AND pharmaceutical representatives
9. Industry sponsored meals AND physician behavior
