# Supplementary material for: Interactions between physicians and the pharmaceutical industry generally and sales representatives specifically and their association with physicians’ attitudes and prescribing habits: a systematic review
Source: BMJ Open. 2017 Sep 27;7(9):e016408. doi: 10.1136/bmjopen-2017-016408 (PMC5623540; doi:10.1136/bmjopen-2017-016408)
Supplement: Author's manuscript [file bmjopen-2017-016408.draft_revisions.pdf]

BMJ Open

BMJ Open is committed to open peer review. As part of this commitment we make the peer review history of every article we publish publicly available.

When an article is published we post the peer reviewers' comments and the authors' responses online. We also post the versions of the paper that were used during peer review. These are the versions that the peer review comments apply to.

The versions of the paper that follow are the versions that were submitted during the peer review process. They are not the versions of record or the final published versions. They should not be cited or distributed as the published version of this manuscript.

BMJ Open is an open access journal and the full, final, typeset and author-corrected version of record of the manuscript is available on our site with no access controls, subscription charges or pay-per-view fees (http://bmjopen.bmj.com).

If you have any questions on BMJ Open's open peer review process please email editorial.bmjopen@bmj.com

BMJ Open

# Interactions between Physicians and Pharmaceutical Industry-Systematic Review

| | |
|---|---|
| Journal: | *BMJ Open* |
| Manuscript ID | bmjopen-2017-016408 |
| Article Type: | Research |
| Date Submitted by the Author: | 18-Feb-2017 |
| Complete List of Authors: | Urbach, Ewout; Crowd for Cure<br>Fickweiler, Freek; Crowd for Cure<br>Fickweiler, Ward; Crowd for Cure, |
| <b>Primary Subject Heading</b>: | Health policy |
| Secondary Subject Heading: | Patient-centred medicine |
| Keywords: | Change management < HEALTH SERVICES ADMINISTRATION & MANAGEMENT, Health policy < HEALTH SERVICES ADMINISTRATION & MANAGEMENT, Protocols & guidelines < HEALTH SERVICES ADMINISTRATION & MANAGEMENT |
| | |

SCHOLARONE™
Manuscripts

# Interactions between Physicians and Pharmaceutical Industry- Systematic Review

Urbach E[1], Fickweiler F[1], Fickweiler W[1]

1: Crowd for Cure, Jacob van Ruysdaelstraat 34, 9718 SG Groningen, the Netherlands

For peer review only

**All authors have completed the ICMJE uniform disclosure form at http://www.icmje.org/coi_disclosure.pdf and declare: no support from any organisation for the submitted work; no financial relationships with any organisations that might have an interest in the submitted work in the previous three years, no other relationships or activities that could appear to have influenced the submitted work**

**Contributor Statement: All authors have contributed equally and have substantial contributions to the conception or design of the work; Author Ewout Urbach for the acquisition, analysis, and interpretation of data for the work; Author Ewout Urbach for drafting the work and Authors Freek Fickweiler and Ward Fickweiler for revising it critically for important intellectual content; and all authors (Ewout Urbach, Freek Fickweiler and Ward Fickweiler) contributed to final approval of the version to be published and agreed to be accountable for all aspects of the work in ensuring that questions related to the accuracy or integrity of any part of the work are appropriately investigated and resolved.**

**Competing interest: no financial relationships with any organisations that might have an interest in the submitted work in the previous three years, no other relationships or activities that could appear to have influenced the submitted work**

**Funding: all authors declare no support from any organisation for the submitted work.**

**Data sharing statement: any data relevant to a published article will be made available alongside the article when published.**

**Abstract**

**Objectives**

The objective of this review is to explore the frequency of physician and pharmaceutical industry interactions, their impact on physicians' attitude, knowledge and behavior

**Data Sources**

Pubmed, Embase, Cochrane library and Google scholar electronic databases were searched from 1992 to August 2016 using free text words and medical subject headings relevant to the topic.

**Study Selection**

Studies included were cross sectional studies, cohort studies, randomized trials and survey designs. Studies with narrative reviews, case reports, opinion polls, letters to the editor, systematic reviews and non-English studies were excluded from data synthesis.

**Data Extraction**

Two reviewers independently extracted the data. Data on study design, study year, country, participant characteristics, setting, and number of participants were collected.

**Data Synthesis**

PSR interactions influences the physicians' attitudes towards the representatives, their prescribing behavior and increases the number of formulary addition requests for the company's drug. Other interactions such as CME and attending pharmaceutical industry sponsored seminars lead to higher prescribing of the company drug and increasing irrational prescribing behavior.

**Conclusion**

Physician-PSR interactions and acceptance of gifts and favors from the company's PSRs have been found to affect the physicians' prescribing behavior and contribute to irrational prescription of the company's drug. Therefore, intervention in the form of policy implementation and education about the implications of these interactions are needed.

**Strengths and limitations of the Study**

For peer review only

- Large up-to-date systematic review of studies exploring the effects of physician and pharmaceutical industry representative interactions and their impact on physician attitudes, knowledge and behavior.
- Pubmed, Embase, Cochrane library and Google scholar electronic databases were not searched before 1992 and other databases were not searched.

## Introduction

The relationship between physicians and the pharmaceutical industry has evoked heated debate since decades[1]. In 2012, pharmaceutical industry spent $89.5 billion on physician-pharmaceutical sales representative (PSR) interactions, accounting for 60% of the global sales and marketing spending[3-6]. Previous reports have demonstrated that PSRs may influence prescribing behavior[,11, 14, 36, 38, 39]. However, the attitudes about PSR interactions are divided and contradictory. Studies have indicated that physicians may be unable to distinguish between promotional information and scientific evidence, while their colleagues more than themselves are susceptible to PSR marketing strategies[22, 27, 32, 34] Most medical and governmental institutions have installed guidelines and self-regulatory and legislative checks to address this controversy[-5,8,9] However, while administration's proposals for deregulatory reforms of Big Pharma are increasing, scientific evidence rigoursly examining this controversy are needed. This review address this question by critically and systemically evaluating the evidence on the impact of PSR interactions on the attitudes of physicians.

## Methodology

*Inclusion and exclusion criteria:*

The following inclusion and exclusion criteria were used to perform this systematic review.

(a) Types of studies: Included for data synthesis in this review were cross sectional studies, cohort studies, randomized trials and survey designs that have used analytical methodologies and have focused on at least one facet of extent, impact on behavior and attitude. Excluded were narrative reviews, case reports, opinion polls, letters to the editor, systematic reviews and non-English studies.

(b) Types of participants: Physicians and pharmaceutical representatives.

(c) Types of exposure: Any type of interaction between physicians and the pharmaceutical industry such as meeting with drug representatives, participating in pharmaceutical-sponsored continuing medical education program, and receiving travel funding, free drug samples, industry-provided meals and gifts.

For peer review

(d) Types of outcome: Knowledge, beliefs, and/or attitudes of physicians regarding physician-industry interactions.

*Search strategy:*

Pubmed, Embase, Cochrane library and Google scholar electronic databases were searched from 1992 to August 2016 using free text words and medical subject heading relevant to the topic. Databases were not searched before 1992, introducing reporting bias. However, while the relationship between physicians and pharmaceutical representatives is likely to change in time, we did not found studies before 1992 to be reflective of this relationship. Search terms were physician, doctor, healthcare professional, attitude, knowledge, behavior, hospital formulary, professional behavior, prescribing behavior, pharmaceutical representative, interests, marketing strategy, research grant, gifts and meals. Two independent reviewers assessed selected articles as per inclusion/exclusion criteria and shortlisted them for writing the review. Full review protocol is available upon request to the corresponding author.

For peer review only

## Results

We independently screened the titles and abstracts of the 2170 identified records for potential eligibility. Out of 2170, full text of 49 eligible citations, which matched the inclusion criteria, were retrieved and used for qualitative assessment during the writing of the review (Figure 1, Table 1).

### *Characteristics of included studies*

The identified studies were published between 1992 and August 2016. Most of the studies included were cross-sectional studies[1, 11-15, 19-23, 26-43, 55, 57, 62, 63, 72, 76]. Only two studies were cohort studies[16, 18], three were randomized trials[17, 25, 74] and one study was a case-control study[24].

### *Extent of interactions between physicians and the pharmaceutical industry*

We found that PSR interactions are a regular feature in the daily lives of physicians across the world [11, 14, 31, 36, 39, 55]. Most of the attending physicians and residents have at least one interaction with the industry representatives per month [14,21, 31, 32, 33]. The frequency of interactions or gifts offered and accepted varies with private versus public hospital setting and the position of the physicians in the medical hierarchy [12, 14, 17, 26, 31, 34, 39, 55, 56]. Medical students are exposed to PSRs from the beginning of their career [34,56]. Junior residents received twice as much free drug samples from PSR interactions than senior residents [14]. PSR interactions were significantly higher at the beginning of residency[39]. The majority of program directors of internal medicine residencies in USA allowed PSRs to meet with residents during working hours and permitted PSR sponsorship of conferences[29]. Attending physicians and physician specialists have greater encounters with PSRs and received more number of medical samples and promotional material than residents[11,31]. Participants working in private practice alone or in both sectors were more likely to receive gifts than doctors working in the public sector [31, 55]. Physicians in academic or hospital-based practice settings had less PSR interactions and significantly lower prescribing costs than physicians in nonacademic and nonhospital practices [26].

For peer review only

Most common gifts received were medical samples[11, 12, 21, 22, 31, 32, 33], promotional material[11, 19, 31] invitations for dinners[11], invitations for CMEs[19, 33], scientific journals[19] and free lunches[22, 32].

### *Attitude of physicians towards the interactions*

We found that physicians have a positive attitude towards PSRs.[1, 12, 13, 17, 23, 27, 29, 33, 39, 43] Physicians perceived PSRs as important sources of education and funding [13, 14, 33, 34, 37, 40]; while some studies reporting skeptical attitudes about contribution of PSRs towards teaching and education [21, 28, 29, 32, 43]. Conference registration fees, informational luncheons, sponsorship of departmental journal clubs, anatomical models, and free drug samples were considered as appropriate gifts [17, 23, 28, 57]. Most of the physicians considered pharmaceutical information provided by PSRs, industry sponsored conferences and CME events as important instruments for enhancing their scientific knowledge [13, 33, 37, 40]. Compared to senior residents, significantly more junior residents felt that pharmaceutical representatives have a valuable teaching role [14].

Most of the physicians considered themselves immune to the influence of gifts[1, 13, 14, 15, 20 22, 25, 34]. We found that better scores on knowledge and attitudes were significantly associated with lesser number of interactions with representatives and their gifts[23]. Most studies found that physicians do not believe that PSR interactions impact their prescribing behavoir [1,11, 14, 36, 38, 39, 66, 67], while other studies report found that there was some extent of influence [19, 21, 22, 28, 32, 33, 34]. In addition, physicians considered their colleagues more susceptible than themselves to PSR marketing strategies [1, 22, 27, 32, 34]. There was a strong correlation between the amount of gifts and the belief that PSR interactions did not influence their prescribing behavior [14].

### *Gifts*

Most common gifts received were medical samples[11, 12, 21, 22, 31, 32, 33, 34, 35, 41], promotional material[11, 19, 31, 58] invitations for dinners[11] and scientific journals[19].

### *Drug samples*

For peer review only

Most of the physicians who accepted drug samples had a positive attitude towards the pharmaceutical representatives[11, 12, 21, 22, 31, 32, 33, 34]. Accepting samples lead to higher branded drug prescription rather than generic prescribing [33, 41].

### *Pharmaceutical representative speakers*

Sponsored lectures/symposia of pharmaceutical companies influenced behavior of the attendees, as they prescribed more drugs of the industry without sufficient evidence supporting the drug's superiority [16, 18]. The majority of attending physicians failed to identify inaccurate information about the company drug[59].

### *Honoraria and Research Funding*

Physicians who received money to attend pharmaceutical symposia or conduct research for the company's drug requested formulary addition of that company's drug more often than other physicians[24] (Table 2). Brief encounters with PSRs and receipt of honoraria or research support were predictors of faculty requested change in hospital formulary[60]. Physicians considered company funded clinical trials with skepticism albeit their prescribing behavior was affected favoring the company's drug[61].

### *Conference travel*

Pharmaceutical company sponsored conference travels to touristic locations have quantifiable impact on the prescribing rational of attendees. A significant increase (three times) in the prescribing rate of two company drugs was observed after the physicians attended a company sponsored symposium with all their expenses covered. Despite this significant difference in the prescribing patterns, physicians insisted there was no impact on their prescribing behaviour.[18]

### *Industry paid lunches*

Most physicians received invitations for dinners[11] and free lunches [14, 20, 32, 34]. Clerks, interns and junior residents attended more company sponsored lunches than senior residents [14]. Pharmaceuticals also sponsored departmental lunches during journal clubs[28]. There was no significant association between attending industry paid lunches[22] and dinners[11] and formulary request for that company's drug (Table 2). However, there was a significant association between attending industry paid lunches and increased prescription of branded drugs [62, 63, 64].

For peer review only

### *CME sponsorship*

Physicians who attended company sponsored CME events had more positive attitudes towards and inclination to prescribe the branded drugs [19, 34, 58, 61, 65]. We found that physicians who refused CME sponsorship were seen to prescribe higher proportion of generics and lower expenditure medicines when compared to physicians who attended CMEs[34].

## Discussion

We report that there is widespread interaction between the pharmaceutical industry and physicians[11, 14, 31, 36, 39, 55]. Interactions are in the form of personal communications, free gifts such as drug samples, sponsored meals, sponsored conference travel, funding for research and CMEs and honoraria[11, 12, 21, 22, 31, 32, 33]. The frequency of these interactions is comparable between residents and physicians[14, 21, 31, 32, 33]. However, the amount and type of gifts vary with the position of the physician in medical hierarchy, specialization and location of practice[12, 14, 17, 26, 31, 34, 39, 55, 56]. In general, trainees (residents, interns) are treated with more drug samples, stationery items and free meals than senior physicians[14, 39]. Senior physicians usually avail of sponsored conferences/ trips, research funding, honoraria and CME events. The extent of these interactions varies with academic versus non-academic institutions: non-academic hospitals record more interactions than others[12, 26, 31, 55, 76]. The majority of the physicians do not believe that they are affected by PSR interactions [1, 13, 14, 15, 20 22, 25, 34]. However, a sizeable percentage in various surveys responded in the affirmative when asked whether they thought that their peers are vulnerable[1, 22, 27, 32, 34].

We observe that there is a positive correlation between acceptance of gifts and physicians' urge to reciprocate favorably towards the benefactor[33, 38, 41]. More the amount and monetary value of the interactions, Considering that physicians have a social contract with society at large to provide unbiased and altruistic service, this is an alarming observation. Countries have put into effect legislation and policies to curb activities that abuse the role of physicians as gatekeepers of society's health, which are discussed below.

### *Policies and educational intervention*

The relationship of physicians with patients is of fiduciary nature. Hence activities that might affect that relationship by altering physicians' clinical behavior are not acceptable. Physician-PSR interactions may put the trust of patients in physicians at risk. Interaction with PSRs

For peer review only

begins at medical school. Trainees are exposed to PSR marketing and promotional techniques from the initial years of their medical education, which impact their prescribing behavior in future. Overall, trainees, i.e., residents and interns, are more vulnerable to PSR interactions than senior physicians[30,36,56]. Physicians are susceptible to PSR interactions, which influences their clinical decision-making leading to greater prescriptions of branded drugs over low cost generic medicines and increasing healthcare cost [33, 41, 62, 63, 64]. In addition, this is accompanied by requests to add the benefactor company's drug to existing hospital formulary[24].. Therefore, there is need to institute and implement stringent policies curtailing physician-PSR relationships, as well as educational programs to increase awareness among medical students in their formative years. Previous reports have indicated that implementing policies and conducting educational programs are effective in increasing awareness of physician's attitudes towards PSR interactions[25,51,71, 72, 73, 74]. .

*Limitiations of the study*

Pubmed, Embase, Cochrane library and Google scholar electronic databases were not searched before 1992 and other databases were not searched.

*Future implications*

PSR interactions compromise the objectivity of the physicians and results in irrational prescribing behavior and increasing healthcare cost Educating physicians and increasing regulation of PSR interactions may lower the likelihood of prescribing new non-superior industry drugs and irrational prescription behavior. Further studies are required to evaluate the benefits of various intervention based education programs on the clinical and ethical behavior of the physicians.

For peer review only

## References


1. Steinman MA, Shlipak MG, McPhee SJ. Of principles and pens: attitudes and practices of medicine housestaff toward pharmaceutical industry promotions. The American journal of medicine. 2001; 110 (7): 551-7.

2. Wazana A. Physicians and the pharmaceutical industry: is a gift ever just a gift? JAMA. 2000; 283(3): 373-80.

3. Wolfe SM. Why do American drug companies spend more than $12 billion a year pushing drugs? Is it education or promotion? Journal of general internal medicine. 1996; 11 (10): 637-9.

4. Wall LL, Brown D. The high cost of free lunch. Obstetrics & Gynecology. 2007; 110 (1): 169-73.

5. Chressanthis GA, Sfekas A, Khedkar P, Jain N, Poddar P. Determinants of pharmaceutical sales representative access limits to physicians. [published online ahead of print April 29, 2015]. J Med Mark.

6. KPMG International. "Rising costs of litigation in pharmaceuti- cals industry." Issues Monitor-Pharmaceuticals. 2011; 9(14): 1-3.

7. Fugh-Berman A, Ahari S. Following the script: how drug reps make friends and influence doctors. In: Sismondo S, Greene JA, eds. The Pharmaceutical Studies Reader. New York, NY: John Wiley; 2015:123-134.

8. Kesselheim AS, Mello MM, Studdert DM. Strategies and practices in off-label marketing of pharmaceuticals: a retrospective analysis of whistleblower complaints. PLoS Med. 2011;8(4): e1000431.

9. Mulinari S. Unhealthy marketing of pharmaceutical products: an international public health concern. J Public Health Policy. 2016; 37 (2): 149-159.

10. Francer J, Izquierdo JZ, Music T, et al. Ethical pharmaceutical promotion and communications worldwide: codes and regulations. Philos Ethics Humanit Med. 2014; 9(1): 7.

11. De Ferrari A, Gentille C, Davalos L, Huayanay L, Malaga G. Attitudes and relationship between physicians and the pharmaceutical industry in a public general hospital in Lima, Peru. PloS one. 2014; 9(6): e100114.


For peer review only

12. Thomson AN, Craig BJ, Barham PM. Attitudes of general practitioners in New Zealand to pharmaceutical representatives. Br J Gen Pract. 1994 May 1; 44(382): 220-3.

13. Kamal S, Holmberg C, Russell J, Bochenek T, Tobiasz-Adamczyk B, Fischer C, Tinnemann P. Perceptions and Attitudes of Egyptian Health Professionals and Policy-Makers towards Pharmaceutical Sales Representatives and Other Promotional Activities. PloS one. 2015; 10(10): e0140457.

14. Hodges B. Interactions with the pharmaceutical industry: experiences and attitudes of psychiatry residents, interns and clerks. CMAJ: Canadian Medical Association Journal. 1995; 153(5): 553.

15. Gibbons RV, Landry FJ, Blouch DL, Jones DL, Williams FK, Lucey CR, Kroenke K. A comparison of physicians' and patients' attitudes toward pharmaceutical industry gifts. Journal of General Internal Medicine. 1998 Mar 1; 13(3): 151-4.

16. Spingarn RW, Berlin JA, Strom BL. When pharmaceutical manufacturers' employees present grand rounds, what do residents remember? Academic Medicine. 1996; 71(1): 86-8.

17. Zaki NM. Pharmacists' and physicians' perception and exposure to drug promotion: A Saudi study. Saudi Pharmaceutical Journal. 2014; 22(6): 528-36.

18. Orlowski JP, Wateska L. The effects of pharmaceutical firm enticements on physician prescribing patterns: there's no such thing as a free lunch. Chest. 1992; 102 (1): 270-3.

19. Scheffer MC. Interaction between pharmaceutical companies and physicians who prescribe antiretroviral drugs for treating AIDS. Sao Paulo Medical Journal. 2014; 132 (1): 55-60.

20. Brett AS, Burr W, Moloo J. Are gifts from pharmaceutical companies ethically problematic?: a survey of physicians. Archives of internal medicine. 2003; 163(18): 2213-8.

21. Gupta SK, Nayak RP, Sivaranjani R. A study on the interactions of doctors with medical representatives of pharmaceutical companies in a Tertiary Care Teaching Hospital of South India. Journal of pharmacy & bioallied sciences. 2016;8(1): 47.

22. Morgan MA, Dana J, Loewenstein G, Zinberg S, Schulkin J. Interactions of doctors with the pharmaceutical industry. Journal of Medical Ethics. 2006; 32(10): 559-63.

23. Alosaimi FD, Al Kaabba A, Qadi M, Albahlal A, Alabdulkarim Y, Alabduljabbar M, Alqahtani F. Physicians' attitudes towards interaction with the pharmaceutical industry/Attitudes des médecins par rapport à l'interaction avec l'industrie pharmaceutique. Eastern Mediterranean Health Journal. 2014; 20(12): 812.

24. Chren MM, Landefeld CS. Physicians' behavior and their interactions with drug companies: a controlled study of physicians who requested additions to a hospital drug formulary. JAMA. 1994; 271(9): 684-9.

25. Randall ML, Rosenbaum JR, Rohrbaugh RM, Rosenheck RA. Attitudes and behaviors of psychiatry residents toward pharmaceutical representatives before and after an educational intervention. Academic Psychiatry. 2005; 29(1): 33-9.

26. Caudill TS, Johnson MS, Rich EC, McKinney WP. Physicians, pharmaceutical sales representatives, and the cost of prescribing. Archives of Family Medicine. 1996; 5(4): 201-6.

27. Andaleeb SS, Tallman RF. Physician attitudes toward pharmaceutical sales representatives. Health care management review. 1995; 20(3): 68-76.

28. Reeder M, Dougherty J, White LJ. Pharmaceutical representatives and emergency medicine residents: a national survey. Annals of emergency medicine. 1993; 22(10): 1593-6.

29. Lichstein PR, Turner RC, O'Brien K. Impact of pharmaceutical company representatives on internal medicine residency programs: a survey of residency program directors. Archives of Internal Medicine. 1992; 152(5): 1009-13.

30. Brotzman GL, Mark DH. Policies regulating the activities of pharmaceutical representatives in residency programs. Journal of family practice. 1992; 34(1): 54-8.

31. Alssageer MA, Kowalski SR. A survey of pharmaceutical company representative interactions with doctors in Libya. Libyan Journal of Medicine. 2012; 7(1).

32. Lieb K, Brandtönies S. A survey of german physicians in private practice about contacts with pharmaceutical sales representatives. Dtsch Arztebl Int. 2010 Jun 4; 107(22): 392-8.

33. Lieb K, Scheurich A. Contact between doctors and the pharmaceutical industry, their perceptions, and the effects on prescribing habits. PloS one. 2014 Oct 16; 9(10): e110130.

34. Lieb K, Koch C. Medical Students' Attitudes to and Contact With the Pharmaceutical Industry: A Survey at Eight German University Hospitals. Dtsch. Arztebl. Int. 2013; 110(35-36): 584-90.

35. Brown SR, Evans DV, Berman AF. Pharmaceutical Industry Interactions in Family Medicine Residencies Decreased Between 2008 and 2013: A CERA Study. Fam Med. 2015; 47(4): 279-82.

36. Riese F, Guloksuz S, Roventa C, Fair JD, Haravuori H, Rolko T et al. Pharmaceutical industry interactions of psychiatric trainees from 20 European countries. Eur Psychiatry. 2014.

For peer review only

37. Rahmana MH, Agarwala S, Tuddenhama S, Petoa H, Iqbalb M, Bhuiyab A, Peters DH. What do they do? Interactions between village doctors and medical representatives in Chakaria, Bangladesh. Int Health. 2015; 7:266–71.

38. Lee D, Begley CE. Physician report of industry gifts and quality of care. Health Care Manage Rev. 2016; 41(3): 275-83.

39. Montastruc F, Moulis G, Palmaro A, Gardette V, Durrieu G, Montastruc JL. Interactions between Medical Residents and Drug Companies: A National Survey after the Mediator Affair. PLoS One. 2014; 9(10): e104828.

40. Ketis ZK, Kersnik J. Which Pharmaceutical Sales Representatives' Features do Slovenian Family Physicians Value?. Acta Inform Med. 2013; 21(4): 257–60.

41. Hurley MP, Stafford RS, Lane AT. Characterizing the Relationship Between Free Drug Samples and Prescription Patterns for Acne Vulgaris and Rosacea. JAMA Dermatol. 2014; 150(5): 487-93.

42. Makowska M. Interactions between Doctors and Pharmaceutical Sales Representatives in a Former Communist Country. Camb. Q. Healthc. Ethics. 2014; 23:349 – 55.

43. Siddiqui UT, Shakoor A, Kiani S, Ali F, Sharif M, Kumar A et al. Attitudes of medical students towards incentives offered by pharmaceutical companies- perspective from a developing nation- a cross sectional study. Bio Med Cent. 2014; 15:36.

44. Pokorny AM, Gittins CB. Dangerous liaisons: doctors-in-training and the pharmaceutical industry. Internal medicine journal. 2015 Oct 1; 45(10): 1085-8.

45. Physicians and the pharmaceutical industry (update 1994). Canadian Medical Association. CMAJ: Canadian Medical Association Journal. 1994; 150(2): 256A-256F.

46. College of Family Physicians of Canada. Royal College of Physicians and Surgeons of Canada. Guide to enhancing referrals and consultations between physicians. Mississauga, ON: College of Family Physicians of Canada; 2009.

47. McMurray RJ, Clarke OW, Barrasso JA, et al. Gifts to Physicians From Industry. JAMA.1991; 265(4): 501.

48. Petersdorf R. Guidelines for faculty involvement in commercially supported continuing medical-education-september 1992-preface.

49. Physicians and the Pharmaceutical Industry. Ann Intern Med. 1990;112:624-626. doi:10.7326/0003-4819-112-8-624.

50. Silverman E. Everything you need to know about the Sunshine Act. BMJ. 2013; 347:f4704.

51. Sergeant MD, Hodgetts PG, Godwin M, Walker DM, McHenry P. Interactions with the pharmaceutical industry: a survey of family medicine residents in Ontario. CMAJ: Canadian Medical Association Journal. 1996 Nov 1; 155 (9): 1243.

52. Carroll AE, Vreeman RC, Buddenbaum J, Inui TS. To what extent do educational interventions impact medical trainees' attitudes and behaviors regarding industry-trainee and industry-physician relationships? Pediatrics. 2007; 120(6): e1528-35.

53. Othman N, Vitry AI, Roughead EE, Ismail SB, Omar K. Medicines information provided by pharmaceutical representatives: a comparative study in Australia and Malaysia. BMC Public Health. 2010; 10(1): 1.

54. Lotfi T, Morsi RZ, Rajabbik MH, Alkhaled L, Kahale L, Nass H, Brax H, Fadlallah R, Akl EA. Knowledge, beliefs and attitudes of physicians in low and middle-income countries regarding interacting with pharmaceutical companies: a systematic review. BMC health services research. 2016; 16 (1): 1.

55. Workneh BD, Gebrehiwot MG, Bayo TA, Gidey MT, Belay YB, Tesfaye DM, Kassa TT. Influence of Medical Representatives on Prescribing Practices in Mekelle, Northern Ethiopia. PLoS One. 2016; 11(6): e0156795.

56. Zipkin DA, Steinman MA. Interactions Between Pharmaceutical Representatives and Doctors in Training. J Gen Intern Med. 2005; 20:777–786.

57. Khan N, Naqvi AA, Ahmad R, Ahmed FR, McGarry K, Fazlani RY, Ahsan M. Perceptions and Attitudes of Medical Sales Representatives (MSRs) and Prescribers Regarding Pharmaceutical Sales Promotion and Prescribing Practices in Pakistan. Journal of Young Pharmacists, 2016; 8(3): 244-250.

58. Saito S, Mukohara K, Bito S. Japanese practicing physicians' relationships with pharmaceutical representatives: a national survey. PLoS One. 2010 Aug 13; 5(8): e12193.

59. Ziegler MG, Lew P, Singer BC. The accuracy of drug information from pharmaceutical sales representatives. JAMA. 1995; 273(16): 1296-8.

60. Lurie N, Rich EC, Simpson DE, Meyer J, Schiedermayer DL, Goodman JL, McKinney WP. Pharmaceutical representatives in academic medical centers: interaction with faculty and housestaff. J Gen Intern Med. 1990; 5(3): 240-3.

61. Lexchin J. Interactions between physicians□and the pharmaceutical industry: What does the literature say? CMAJ. 1993 Nov 15; 149(10): 1401–1407.

62. DeJong C, Aguilar T, Tseng CW, Lin GA, Boscardin WJ, Dudley RA. Pharmaceutical Industry-Sponsored Meals and Physician Prescribing Patterns for Medicare Beneficiaries. JAMA Intern Med. 2016; 176(8): 1114-10.

63. Yeh JS, Franklin JM, Avorn J, Landon J, Kesselheim AS. Association of Industry Payments to Physicians With the Prescribing of Brand-name Statins in Massachusetts. JAMA Intern Med. 2016;176(6):763-8.

64. Steinbrook R. Industry Payments to Physicians and Prescribing of Brand-name Drugs. JAMA Intern Med. 2016 Aug 1;176(8):1123.

65. Bowman MA, Pearle DL. Changes in drug prescribing patterns related to commercial company funding of continuing medical education. J. Contin. Educ. Health Prof. 1988;8(1): 13-20.

66. Fischer MA, Keough ME, Baril JL, et al. Prescribers and pharmaceutical representatives: why are we still meeting? J Gen Intern Med. 2009; (7): 795-801.

67. Chimonas S, Brennan TA, Rothman DJ. Physicians and drug representatives: exploring the dynamics of the relationship. J Gen Intern Med. 2007; 22(2): 184-190.

68. Grande D. Limiting the influence of pharmaceutical industry gifts on physicians: self-regulation or government intervention? J Gen Intern Med. 2010; 25(1): 79-83.

69. Brennan TA, Rothman DJ, Blank L, Blumenthal D, Chimonas SC, Cohen JJ, Goldman J, Kassirer JP, Kimball H, Naughton J, Smelser N. Health industry practices that create conflicts of interest: a policy proposal for academic medical centers. JAMA. 2006; 295(4): 429-433.

70. Greenland P. Time for the medical profession to act: new policies needed now on interactions between pharmaceutical companies and physicians. Arch Intern Med. 2009; 169(9): 829-831.

71. Montague BT, Fortin AH 6th, Rosenbaum J. A systematic review of curricula on relationships between residents and the pharmaceutical industry. Med Educ. 2008; 42(3): 301-8.

72. Yeh JS, Austad KE, Franklin JM, Chimonas S, Campbell EG, Avorn J, Kesselheim AS. Association of medical students' reports of interactions with the pharmaceutical and medical device industries and medical school policies and characteristics: a cross-sectional study. PLoS Med. 2014; 11(10): e1001743.

73. Larkin I, Ang D, Avorn J, Kesselheim AS. Restrictions on pharmaceutical detailing reduced off-label prescribing of antidepressants and antipsychotics in children. Health Aff (Millwood). 2014; 33(6): 1014-23.

74. Esmaily HM, Silver I, Shiva S, Gargani A, Maleki-Dizaji N, Al-Maniri A, Wahlstrom R. Can rational prescribing be improved by an outcome-based educational approach? A randomized trial completed in Iran. J. Contin. Educ. Health Prof. 2010 Winter; 30 (1): 11-8.

75. Shalowitz DI, Spillman MA, Morgan MA. Interactions with industry under the Sunshine Act: an example from gynecologic oncology. Am. J. Obstet. Gynecol. 2016; 214(6): 703-7

76. Parikh K, Fleischman W, Agrawal S. Industry Relationships With Pediatricians: Findings From the Open Payments Sunshine Act. Pediatrics. 2016; 137(6).

77. Birkhahn RH, Jauch E, Kramer DA, Nowak RM, Raja AS, Summers RL, Weber JE, Diercks DB. A review of the federal guidelines that inform and influence

For peer review only

relationships between physicians and industry. Acad. Emerg. Med. 2009; 16(8): 776-81.

78. Chressanthis GA, Khedkar P, Jain N, Poddar P, Seiders MG. Can access limits on sales representatives to physicians affect clinical prescription decisions? A study of recent events with diabetes and lipid drugs. J Clin Hypertens (Greenwich). 2012; 14(7): 435-46.

79. Grundy Q, Bero L, Malone R. Interactions between non-physician clinicians and industry: a systematic review. PLoS Med. 2013;10(11): e1001561.

For peer review only

**Legends**

**Figure 1:** PRISMA flow diagram showing search strategy and included studies

**Table 1:** Characteristics of included studies

**Table 2:** Impact of physician-pharmaceutical industry interaction on physician

**The Corresponding Author has the right to grant on behalf of all authors and does grant on behalf of all authors, a worldwide licence (http://www.bmj.com/sites/default/files/BMJ%20Author%20Licence%20March%2020 13.doc) to the Publishers and its licensees in perpetuity, in all forms, formats and media (whether known now or created in the future), to i) publish, reproduce, distribute, display and store the Contribution, ii) translate the Contribution into other languages, create adaptations, reprints, include within collections and create summaries, extracts and/or, abstracts of the Contribution and convert or allow conversion into any format including without limitation audio, iii) create any other derivative work(s) based in whole or part on the on the Contribution, iv) to exploit all subsidiary rights to exploit all subsidiary rights that currently exist or as may exist in the future in the Contribution, v) the inclusion of electronic links from the Contribution to third party material where-ever it may be located; and, vi) licence any third party to do any or all of the above. All research articles will be made available on an Open Access basis (with authors being asked to pay an open access fee—see http://www.bmj.com/about-bmj/resources-**

authors/forms-policies-and-checklists/copyright-open-access-and-permission-reuse).
The terms of such Open Access shall be governed by a Creative Commons licence—details as to which Creative Commons licence will apply to the research article are set out in our worldwide licence referred to above.

For peer review only

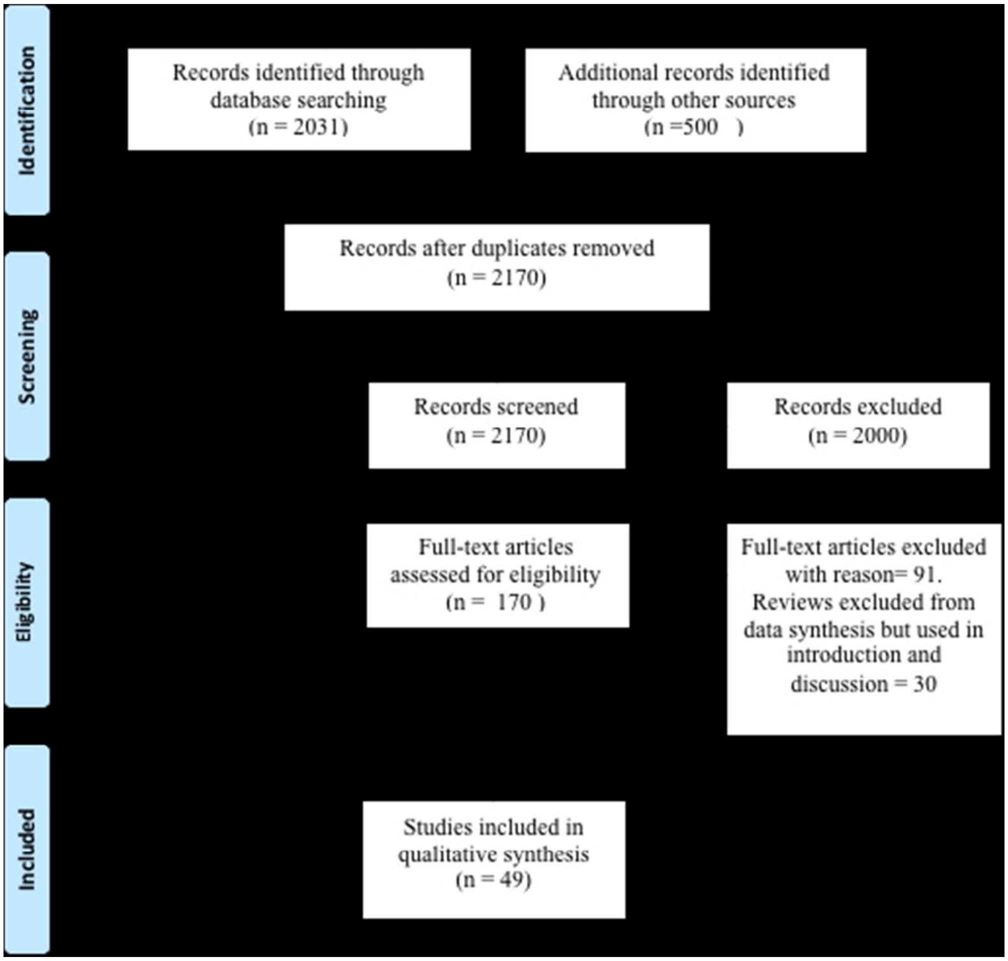


PRISMA flow diagram showing search strategy and included studies

180x171mm (72 x 72 DPI)

| Study | Authors | Study sample | Study design | Interaction | Summary |
|---|---|---|---|---|---|
| 1 | Steinman et al., 2001 | Residents | Cross-sectional | Interaction with representative, drug samples | Impact on prescribing |
| 11 | De Ferrari et al., 2014 | Physicians | Cross-sectional | Interaction, medical samples, promotional material, dinners | Positive attitude towards representatives |
| 12 | Thomson et al., 1994 | Physicians | Cross-sectional | Interaction with representative, drug samples | Positive attitude towards industry |
| 13 | Kamal et al., 2015 | Physicians | Cross-sectional | Interaction with representative | Positive attitude towards industry |
| 14 | Hodges, 1995 | Residents of psychiatry | Cross-sectional | Interaction with representative | Positive attitude towards gifts |
| 15 | Gibbons et al., 1998 | Physicians and residents | Cross-sectional | Gifts, samples, travel, lunches | Positive attitude towards gifts |
| 16 | Spingarn et al., 1996 | Internal medicine residents | Cohort | Teaching | Negative effect on prescribing |
| 17 | Zaki, 2014 | Physicians | Randomized, cross-sectional survey | Conferences, drug samples | Favorable towards promotion |
| 18 | Orlowski et al., 1994 | Physicians | Cohort | Conference travel | Negative effect on prescribing |
| 19 | Scheffer et al., 2014 | Physicians prescribing antiretroviral drugs | Cross-sectional | Interaction with representative, drug samples, journals | Frequency of interaction |
| 20 | Brett et al., 2003 | Physicians | Cross-sectional | Interaction with representative | Impact on attitudes |
| 21 | Gupta et al., 2016 | Doctors | Cross-sectional | Interaction with representative, drug samples, journals | Impact on prescribing |
| 22 | Morgan et al., 2006 | Obstetrician-gynaecologists | Cross-sectional | Drug samples, lunch | Impact on prescribing, positive attitudes |
| 23 | Alosaimi et al., 2014 | Physicians | Cross-sectional | Interaction with | Positive attitude |

For peer review only

| | | | | | |
|---|---|---|---|---|---|
| | | | | representative | towards industry |
| 24 | Chren et al., 1994 | Faculty physicians | Case control | Honoraria, research | Requested formulary additions of drug |
| 25 | Randall et al., 2005 | Residents | Controlled trial | Interaction with representative | Impact on prescribing and attitudes |
| 26 | Caudil et al., 1996 | Physicians | Cross-sectional | Interaction with representative | Negative impact on prescribing patterns |
| 27 | Andaleeb et al., 1995 | Physicians | Cross-sectional | Interaction with representative | Positive attitude towards industry |
| 28 | Reeder et al., 1993 | Residents of emergency medicine | Cross-sectional | Interaction with representative | Believed that no impact on their prescribing |
| 29 | Lichstein et al., 1992 | Directors, internal medicine | Cross-sectional | Interaction with representative | Positive attitude towards industry |
| 30 | Brotzman et al., 1992 | Directors, residency programs | Cross-sectional | Interaction with representative | No guidelines for interaction with representatives |
| 31 | Alssageer et al., 2012 | Doctors | Cross-sectional | Interaction with representative, drug samples, printed materials | Positive attitude towards industry |
| 32 | Lieb & Brandtonies, 2010 | Physicians | Cross-sectional | Interaction with representative, drug samples, printed materials, lunches | Frequency and impact on attitudes |
| 33 | Lieb & Scheurich, 2014 | Physicians | Cross-sectional | Interaction with representative, drug samples, printed materials, CME | High expenditure prescribing |
| 34 | Lieb & Koch, 2013 | Medical students | Cross-sectional | Interaction with representative, drug samples, printed | Positive attitude towards industry, impact on their |

For peer review only

| | | | | | |
|---|---|---|---|---|---|
| | | | | materials, lunches | prescribing |
| 35 | Brown et al., 2015 | Directors, residency programs | Cross-sectional | Interaction with representative, gifts, lunches | Negative attitude towards industry |
| 37 | Rahmana et al., 2015 | Doctors | Cross-sectional | Interaction with representative | Impact on their prescribing |
| 38 | Lee & Begley, 2016 | Physicians | Cross-sectional | Gifts | Negative impact on their prescribing |
| 39 | Montastruc et al., 2014 | Medical residents | Cross-sectional | Interaction with representative | Negative attitude towards industry Believed that no impact on their prescribing |
| 40 | Ketis & Kersnik, 2013 | Family physicians | Cross-sectional | Interaction with representative | Positive effect on knowledge |
| 41 | Hurley et al., 2014 | Dermatologists | Cross-sectional | Free drug samples | Impact on their prescribing (less generic prescribing) |
| 42 | Makowska, 2014 | Doctors | Cross-sectional | Gifts | Positive attitude towards industry |
| 43 | Siddiqui et al., 2014 | Medical students | Cross-sectional | Interaction with representative | Positive attitude towards industry |
| 55 | Workneh BD et al., 2016 | Physicians | Cross-sectional | Interaction with representative, gifts | Positive attitude towards industry, impact on prescribing behavior |
| 57 | Khan N et al., 2016 | Doctors | Cross-sectional | Interaction with representative, gifts | Positive attitude towards industry |
| 58 | Saito S et al. 2010 | Physicians | National Survey | Interaction with industry, receipt of gifts, funds, CME, samples | Positive attitude towards representatives and gifts, value information from representatives, |

For peer review only

| | | | | | |
|---|---|---|---|---|---|
| | | | | | interactions higher with physicians who prefer to prescribe brand names. |
| 59 | Ziegler MG et al. 1995 | Pharmaceutical representative speakers | Survey | Accuracy of information provided by PSRs about drugs | Incorrect information often provided by speakers goes unnoticed by physicians |
| 60 | Lurie N et al., 1990 | Internal medicine house staff and faculty | Survey | Effect of conversation with PSRs, free meals, honoraria and research support | Impact on prescribing behavior and formuary change requests. |
| 62 | DeJong C et al., 2016 | Physicians | Cross-sectional | Industry sponsored meals | Receipt of industry-sponsored meals was associated with an increased rate of brand name prescription. |
| 63 | Yeh JS et al., 2016 | Physicians | Cross-sectional | Effect of industry payment on prescription of branded drugs for cholesterol control | Payment for meals and educational programs increased prescription of brand names. |
| 65 | Bowman MA et al., 1988 | Physician attendees | Self report survey | Effect of CME on prescribing behavior | Sponsoring company's drugs were favored during prescription |
| 66 | Fischer MA et al., 2009 | Physicians, trainees | Survey of focused groups | Effect of industry marketing strategies on prescription and cognitive dissonance of physicians | Believed that no impact on their prescribing, have ability to evaluate information of PSRs |
| 67 | Chimonas S et al., 2007 | Physicians | Survey of focused groups | Determine how physicians handle their cognitive | Physicians understood the conflict of interests but developed |

For peer review only

| | | | | | |
|---|---|---|---|---|---|
| | | | | dissonance | denials and rationalizations to deal with cognitive dissonance. |
| 72 | Yeh JS et al., 2014 | Medical students | Cross-sectional | Interaction with representative, gifts, lunches | Policies separating students from representatives reduced number of interactions |
| 73 | Larkin I et al., 2014 | Pediatricians, child & adolescent psychitrist | Survey | Interaction with representative | Anti detailing policies reduced the prescription of off-label antidepressants and anti psychotics for children |
| 74 | Esmaily HM et al., 2010 | General physicians | Randomized trial | Effect of outcome based CME | Outcome based CME reduced total number of prescriptions, prescriptions of antibiotics, anti-inflammatories and injections compared to traditional CME. It also improved compliance to regulations. |
| 76 | Parikh K et al., 2016 | Pediatricians | Cross-sectional | Comparison of industry interactions between pediatricians and other specialists; among subspecialities of pediatrics. | Pediatricians get fewer gifts from industry than internists. There is variation among sub specialities for extent of interaction. |
| 78 | Chressanthis GA et al. 2012 | Physicians | Survey | Effect of restricting PSRs on clinical practice and knowledge | Restricting PSRs affected information flow about drugs, both negative and positive. |

For peer review only

| # | Attitudes | Prescribing behavior | Knowledge | Formulary requests |
|---|---|---|---|---|
| **Gifts** | Receiving higher number of gifts was associated with belief that PRs have no impact on their prescribing behaviour ($p<0.05$)[14] | - | - | - |
| **Drug samples** | Positive attitude towards the drug industry and the representatives[11, 12, 21, 22, 31, 32, 33, 34] | Higher prescription of the company drug[21, 33, 41] | - | - |
| **Pharmaceutical representative speakers** | - | Rational treatment (OR, 8.4; 95% CI, 2.1-38.9) Irrational treatment ($p= 0.03$ [16]) [16, 18, 34] | Inability to identify the false claims[16, 59] | Request for sponsor's drug vs physicians who did not benefit (OR, 3.9; 95% CI, 1.2-12.7)[24] |
| **Honoraria and Research Funding** | Positive attitude towards sponsor's drug ($p<0.05$ [19])[19, 60, 61] | - | - | Request for sponsor's drug vs physicians who did not benefit (OR, 3.9; 95% CI, 1.2-12.7)[24] |
| **Conference travel** | - | Significant increase in prescribing of sponsor drug (about 3 times higher than before attending) ($p<0.001$)[18] | - | Request for sponsor's drug vs physicians who did not benefit (OR, 3.9; 95% CI, 1.2-12.7)[24] |
| **Industry paid lunches** | Positive attitude towards sponsor's drug ($p<0.05$)[19, 14, 20, 32, 34] | Significant increase in prescribing of sponsor drug [62, 63, 64] | - | There was no significant association between attending the industry paid lunches[21] and dinners[11] and formulary request for that company drug |
| **CME sponsorship** | Positive attitude towards sponsor's drug ($p<0.05$ [19]) [19, 34, 58, 61, 65] | High expenditure prescribing[34] | | |

| | | | | |
|---|---|---|---|---|
| **Interaction with PR** | Interaction with PR was associated with positive attitude towards PR (p= 0.02) Positive attitude towards the gifts, travel, samples, etc ($r$ = 0.706; p = 0.02)[30] | Higher prescription of the company drug[21] | A significant positive correlation was found between the physicians' prescribing cost and the information provided by the drug representative during the interaction (P < 0.01)[26] | Interaction with PR resulted in increased request for PR's drug vs physicians who had no interactions with PR (OR, 3.4; 95% CI, 1.8-6.6)[24] |

OR-Odds ratio, PR-Pharmaceutical representative, CI-Confidence interval

For peer review only

# PRISMA 2009 Checklist

| Section/topic | # | Checklist item | Reported on page # |
|---|---|---|---|
| **TITLE** | | | |
| Title | 1 | Identify the report as a systematic review, meta-analysis, or both. | 1 |
| **ABSTRACT** | | | |
| Structured summary | 2 | Provide a structured summary including, as applicable: background; objectives; data sources; study eligibility criteria, participants, and interventions; study appraisal and synthesis methods; results; limitations; conclusions and implications of key findings; systematic review registration number. | 2 |
| **INTRODUCTION** | | | |
| Rationale | 3 | Describe the rationale for the review in the context of what is already known. | 3 |
| Objectives | 4 | Provide an explicit statement of questions being addressed with reference to participants, interventions, comparisons, outcomes, and study design (PICOS). | 3 |
| **METHODS** | | | |
| Protocol and registration | 5 | Indicate if a review protocol exists, if and where it can be accessed (e.g., Web address), and, if available, provide registration information including registration number. | 4 |
| Eligibility criteria | 6 | Specify study characteristics (e.g., PICOS, length of follow-up) and report characteristics (e.g., years considered, language, publication status) used as criteria for eligibility, giving rationale. | 3 |
| Information sources | 7 | Describe all information sources (e.g., databases with dates of coverage, contact with study authors to identify additional studies) in the search and date last searched. | 4 |
| Search | 8 | Present full electronic search strategy for at least one database, including any limits used, such that it could be repeated. | 4 |
| Study selection | 9 | State the process for selecting studies (i.e., screening, eligibility, included in systematic review, and, if applicable, included in the meta-analysis). | 3/4 |
| Data collection process | 10 | Describe method of data extraction from reports (e.g., piloted forms, independently, in duplicate) and any processes for obtaining and confirming data from investigators. | 4 |
| Data items | 11 | List and define all variables for which data were sought (e.g., PICOS, funding sources) and any assumptions and simplifications made. | 4 |
| Risk of bias in individual studies | 12 | Describe methods used for assessing risk of bias of individual studies (including specification of whether this was done at the study or outcome level), and how this information is to be used in any data synthesis. | 3 |
| Summary measures | 13 | State the principal summary measures (e.g., risk ratio, difference in means). | 3 |
| Synthesis of results | 14 | Describe the methods of handling data and combining results of studies, if done, including measures of consistency (e.g., $I^2$) for each meta-analysis. | 3 |

For peer review only - http://bmjopen.bmj.com/site/about/guidelines.xhtml

# PRISMA 2009 Checklist



| Section/topic | # | Checklist item | Reported on page # |
|---|---|---|---|
| Risk of bias across studies | 15 | Specify any assessment of risk of bias that may affect the cumulative evidence (e.g., publication bias, selective reporting within studies). | 3 |
| Additional analyses | 16 | Describe methods of additional analyses (e.g., sensitivity or subgroup analyses, meta-regression), if done, indicating which were pre-specified. | 3 |
| **RESULTS** | | | |
| Study selection | 17 | Give numbers of studies screened, assessed for eligibility, and included in the review, with reasons for exclusions at each stage, ideally with a flow diagram. | 5 |
| Study characteristics | 18 | For each study, present characteristics for which data were extracted (e.g., study size, PICOS, follow-up period) and provide the citations. | 5-8 |
| Risk of bias within studies | 19 | Present data on risk of bias of each study and, if available, any outcome level assessment (see item 12). | 5-8 |
| Results of individual studies | 20 | For all outcomes considered (benefits or harms), present, for each study: (a) simple summary data for each intervention group (b) effect estimates and confidence intervals, ideally with a forest plot. | 5-8 |
| Synthesis of results | 21 | Present results of each meta-analysis done, including confidence intervals and measures of consistency. | NA |
| Risk of bias across studies | 22 | Present results of any assessment of risk of bias across studies (see Item 15). | 5-8 |
| Additional analysis | 23 | Give results of additional analyses, if done (e.g., sensitivity or subgroup analyses, meta-regression [see Item 16]). | NA |
| **DISCUSSION** | | | |
| Summary of evidence | 24 | Summarize the main findings including the strength of evidence for each main outcome; consider their relevance to key groups (e.g., healthcare providers, users, and policy makers). | 8-9 |
| Limitations | 25 | Discuss limitations at study and outcome level (e.g., risk of bias), and at review-level (e.g., incomplete retrieval of identified research, reporting bias). | 9 |
| Conclusions | 26 | Provide a general interpretation of the results in the context of other evidence, and implications for future research. | 9 |
| **FUNDING** | | | |
| Funding | 27 | Describe sources of funding for the systematic review and other support (e.g., supply of data); role of funders for the systematic review. | 1 |

*From:* Moher D, Liberati A, Tetzlaff J, Altman DG, The PRISMA Group (2009). Preferred Reporting Items for Systematic Reviews and Meta-Analyses: The PRISMA Statement. PLoS Med 6(6): e1000097. doi:10.1371/journal.pmed1000097

For more information, visit: **www.prisma-statement.org**.



For peer review only - http://bmjopen.bmj.com/site/about/guidelines.xhtml

BMJ Open

# Association between pharmaceutical sales representatives' interaction on physicians' attitudes and prescribing habits: A systematic review

| | |
|---|---|
| Journal: | *BMJ Open* |
| Manuscript ID | bmjopen-2017-016408.R1 |
| Article Type: | Research |
| Date Submitted by the Author: | 03-May-2017 |
| Complete List of Authors: | Fickweiler, Freek; Crowd for Cure<br>Fickweiler, Ward; Crowd for Cure,<br>Urbach, Ewout; Crowd for Cure |
| <b>Primary Subject Heading</b>: | Health policy |
| Secondary Subject Heading: | Patient-centred medicine |
| Keywords: | Change management < HEALTH SERVICES ADMINISTRATION & MANAGEMENT, Health policy < HEALTH SERVICES ADMINISTRATION & MANAGEMENT, Protocols & guidelines < HEALTH SERVICES ADMINISTRATION & MANAGEMENT |
| | |

SCHOLARONE™
Manuscripts

# Association between pharmaceutical sales representatives' interaction on physicians' attitudes and prescribing habits: A systematic review

**Fickweiler F[1], Fickweiler W[1], Urbach E[1],**

**1: Crowd for Cure, Jacob van Ruysdaelstraat 34, 9718 SG Groningen, the Netherlands,**
**Correspondence to: F. Fickweiler, MD; freek@crowdforcure.com**

For peer review only

**All authors have completed the ICMJE uniform disclosure form at http://www.icmje.org/coi_disclosure.pdf and declare: no support from any organisation for the submitted work; no financial relationships with any organisations that might have an interest in the submitted work in the previous three years, no other relationships or activities that could appear to have influenced the submitted work**

**Contributor Statement: All authors have contributed equally and have substantial contributions to the conception or design of the work; Author Freek Fickweiler for the acquisition, analysis, and interpretation of data for the work; Author Freek Fickweiler for drafting the work and Authors Ewout Urbach and Ward Fickweiler for revising it critically for important intellectual content; and all authors (Freek Fickweiler, Ward Fickweiler and Ewout Urbach) contributed to final approval of the version to be published and agreed to be accountable for all aspects of the work in ensuring that questions related to the accuracy or integrity of any part of the work are appropriately investigated and resolved.**

**Competing interest: no financial relationships with any organisations that might have an interest in the submitted work in the previous three years, no other relationships or activities that could appear to have influenced the submitted work**

**Funding: all authors declare no support from any organisation for the submitted work.**

**Data sharing statement: any data relevant to a published article will be made available alongside the article when published.**

**Abstract**

**Objectives**

The objective of this review is to explore the frequency of physician and pharmaceutical industry interactions, their impact on physicians' attitude, knowledge and behavior.

**Data Sources**

Pubmed, Embase, Cochrane library and Google scholar electronic databases were searched from 1992 to August 2016 using free text words and medical subject headings relevant to the topic.

**Study Selection**

Studies included were cross sectional studies, cohort studies, randomized trials and survey designs. Studies with narrative reviews, case reports, opinion polls, letters to the editor, systematic reviews and non-English studies were excluded from data synthesis.

**Data Extraction**

Two reviewers independently extracted the data. Data on study design, study year, country, participant characteristics, setting, and number of participants were collected.

**Data Synthesis**

Pharmaceutical sales representative (PSR) interactions influences the physicians' attitudes towards the representatives, their prescribing behavior and increases the number of formulary addition requests for the company's drug. Other interactions such as continuing medical education (CME) and attending pharmaceutical industry sponsored seminars lead to higher prescribing of the company drug and increasing irrational prescribing behavior.

**Conclusion**

Physician-pharmaceutical sales representatives interactions and acceptance of gifts and favors from the company's pharmaceutical sales representatives have been found to affect the physicians' prescribing behavior and are likely to contribute to irrational prescription of the company's drug. Therefore, intervention in the form of policy implementation and education about the implications of these interactions are needed.

**Strengths and limitations of the study**

For peer review only

- Large up-to-date systematic review of studies exploring the effects of physician and pharmaceutical industry representative interactions and their impact on physician attitudes, knowledge and behavior.

- This systematic review used the recommendations outlined in the Cochrane Handbook for conducting systematic reviews and the GRADE methodology to assess the quality of the evidence by outcome.

- Pubmed, Embase, Cochrane library and Google scholar electronic databases were searched from 1992, as well as grey literature.

- Most studies identified were observational and of varying methodological design

- Some studies did not provide evidence for the significance of their findings

**Keywords:** pharmaceutical sales representative; physicians, drug industry; brand prescriptions; conflict of interest ;physicians-industry interactions; pharmaceutical industry; attitude of health personnel; gifts to physicians; medical education; irrational prescriptions

For peer review only

## Introduction

The relationship between physicians and the pharmaceutical industry has evoked heated debate for many decades [1]. In 2012, the pharmaceutical industry spent $89.5 billion on physician-pharmaceutical sales representative (PSR) interactions, accounting for 60% of the global sales and marketing spending [2-8]. Previous reports have demonstrated that PSRs may influence prescribing behavior [9-16]. However, the attitudes about PSR interactions are divided and contradictory. Studies have indicated that physicians may be unable to distinguish between promotional information and scientific evidence [17, 18]. Physicians on the other hand believe their colleagues are more susceptible to PSR marketing strategies than themselves [19-22]. Most medical and governmental institutions have installed guidelines and self-regulatory and legislative checks to address this controversy [5, 15, 16, 23-26]. However, while administrative proposals for deregulatory reforms of the pharmaceutical industry are increasing, scientific evidence rigorously examining this controversy are needed. This review addresses this question by critically and systemically evaluating the evidence on the impact of PSR interactions on the attitudes of physicians.

For peer re

## Methodology

*Protocol*

We followed a detailed methodology that we described in our review protocol, which is available upon request to the corresponding author. Two independent reviewers assessed selected articles as per inclusion/exclusion criteria as per standardization in the protocol, shortlisted them for writing the review and cross-checked each other. The review followed the Preferred Reporting Items for Systematic Reviews and Meta-Analyses (PRISMA) guidelines (Appendix 1).

*Eligibility criteria*

The eligibility criteria were:

- Types of studies: cross sectional studies, cohort studies, randomized trials and survey designs comparing an intervention of interest to a comparator on at least one facet of extent, impact on behavior and attitude. Excluded were narrative reviews, case reports, opinion polls, letters to the editor and systematic reviews.
- Types of participants: physicians, pharmaceutical representatives, physicians in training/residents. We did not consider medical students or other health professionals.

For peer review only - http://bmjopen.bmj.com/site/about/guidelines.xhtml

- Types of exposure: any type of interaction between physicians and the pharmaceutical industry where there is direct interaction with the physician, such as meeting with drug representatives, participating in pharmaceutical-sponsored CME event, receiving travel funding, free drug samples, industry-provided meals, gifts to the individual and active presentation of industry-related information to the physician.
- Types of outcome: knowledge of physicians (e.g. accuracy of knowledge related to a specific medication), beliefs and/or attitudes of physicians regarding physician-industry interactions (e.g. perceived influence of information from the pharmaceutical company on their behavior), behavior of physicians (e.g. prescribing quantity/frequency).Type of control: either not interaction or a lower level of interaction.
- Exclusion criteria were: qualitative, ecological, econometric studies, editorials, letters to the editor, studies on medical students, small samples sizes, studies assessing non-targeted or indirect interactions (e.g. journal advertisement) and research funding.

We did not exclude studies based on risk of bias. We took risk of bias into account when grading the quality of evidence using GRADE approach.

*Search strategy*

The search strategy included Pubmed, Embase, Cochrane library and Google scholar electronic databases (January 1992 to August 2016). Databases were not searched before 1992, as these studies were already investigated in an earlier review [27]. The search combined terms for physicians and pharmaceutical, and included both free text words and medical subject heading relevant to the topic. We did not use a search filter. The supplementary information file provides the full details of the search strategies. Additional search strategies included a search of the grey literature (theses and dissertations). Also, we reviewed the references lists of included and relevant papers [27-29].

*Assessment of risk of bias in included studies*

Two reviewers assessed in duplicate and independently the risk of bias in each eligible study. They resolved disagreements by discussion or with the help of a third reviewer. We used the recommendations outlined in the Cochrane Handbook to assess the risk of bias in randomized

For peer review only - http://bmjopen.bmj.com/site/about/guidelines.xhtml

studies. We graded each potential source of bias and rated the studies as high, low or unclear risk of bias.

*Data analysis and synthesis*

We assessed the agreement between reviewers for full-text screening by calculating the kappa statistic. We did not conduct a meta-analysis due to the heterogeneity of study design, types of interventions, outcomes assessed, and outcome measures used. Instead, we summarized the data narratively. We assessed the quality of evidence by outcome using the GRADE methodology [30].

**Results**

We independently screened the titles and abstracts of the 2170 identified records for potential eligibility. Out of 2170, full text of 49 eligible citations, which matched the inclusion criteria, were retrieved and used for qualitative assessment during the writing of the review (Figure 1, Table 1). We excluded 2000 records as they were not relevant (n = 1641), not original research (n=269), about medical students (n=4) and non-medical (e.g. ecological, econometric; n=86).

*Characteristics of included studies*

The identified studies were published between 1992 and August 2016. Most of the studies included were cross-sectional studies [1, 9-13, 19, 21, 22, 31-55]. Only two studies were cohort studies [56, 57], three were randomized trials [58-60] and one study was a case-control study [61].

*Extent of interactions between physicians and the pharmaceutical industry*

We found that PSR interactions are a regular feature in the daily lives of physicians across the world [9-11, 13, 42, 50]. Most of the attending physicians and residents have at least one interaction with the industry representatives per month [10, 21, 22, 36, 42]. The frequency of interactions or gifts offered and accepted varies with private versus public hospital setting and the position of the physicians in the medical hierarchy [10, 13, 31, 38, 42, 43, 50, 58, 62]. Junior residents received twice as much free drug samples from PSR interactions than senior residents [10]. PSR interactions were significantly higher at the beginning of residency [13]. The majority of program directors of internal medicine residencies in USA allowed PSRs to meet with residents during working hours and permitted PSR sponsorship of conferences [40]. Attending physicians and physician specialists have greater encounters with PSRs and received more

For peer review only

numbers of medical samples and promotional material than residents [9, 42]. Participants working in private practice alone or in both sectors were more likely to receive gifts than physicians working in the public sector [42, 50]. Physicians in academic or hospital-based practice settings had less PSR interactions and significantly lower prescribing costs than physicians in nonacademic and nonhospital practices [38]. Most common gifts received were medical samples [9, 21, 22, 31, 36, 37, 42, 63], promotional material [9, 34, 42] invitations for dinners [9], invitations for CMEs [22, 34], scientific journals [34] and free lunches [21, 37].

*Attitude of physicians towards the interactions*

We found that physicians have a positive attitude towards PSRs [1, 13, 19, 20, 22, 31, 32, 40, 49, 58, 64]. Physicians perceived PSRs as important sources of education and funding [10, 22, 32, 43, 45, 46], while some studies reporting skeptical attitudes about contribution of PSRs towards teaching and education [21, 36, 39, 40, 49]. Conference registration fees, informational luncheons, sponsorship of departmental journal clubs, anatomical models, and free drug samples were considered as appropriate gifts [19, 39, 51, 58]. Most of the physicians considered pharmaceutical information provided by PSRs, industry sponsored conferences and CME events as important instruments for enhancing their scientific knowledge [22, 32, 45, 46]. Compared to senior residents, significantly more junior residents felt that pharmaceutical representatives have a valuable teaching role [10]. Most of the physicians considered themselves immune to the influence of gifts [1, 10, 32, 33, 35, 37, 43, 59]. We found that better scores on knowledge and attitudes were significantly associated with lesser number of interactions with representatives and their gifts [19]. Most studies found that physicians do not believe that PSR interactions impact their prescribing behavoir [1, 9-13, 65, 66], while other studies found that there was some extent of influence [21, 22, 34, 36, 37, 39, 43]. In addition, physicians considered their colleagues more susceptible than themselves to PSR marketing strategies [1, 20, 21, 37, 43]. There was a strong correlation between the amount of gifts and the belief that PSR interactions did not influence their prescribing behavior [10].

*Gifts*

Most common gifts received were medical samples [9, 21, 22, 31, 36, 37, 42-44, 47], promotional material [9, 34, 42, 67] invitations for dinners [9] and scientific journals [34].

For peer review only

*Drug samples*

Most of the physicians who accepted drug samples had a positive attitude towards the pharmaceutical representatives [9, 21, 22, 31, 36, 37, 42, 43]. Accepting samples lead to higher branded drug prescription rather than generic prescribing [22, 47].

*Pharmaceutical representative speakers*

Sponsored lectures/symposia of pharmaceutical companies influenced behavior of the attendees leading to the attendees prescribing more drugs from the sponsoring companies without sufficient evidence supporting superiority of those drugs [56, 57]. The majority of attending physicians failed to identify inaccurate information about the company drug [18]. This might make them more prone to interactions of pharmaceutical sales representatives to prescribe in favor of the company drug.

*Honoraria and Research Funding*

Physicians who received money to attend pharmaceutical symposia or to perform research requested formulary addition of the company's drug more often than other physicians, This association was independent of many confounding factors [61] (Table 2). Brief encounters with PSRs and receipt of honoraria or research support were predictors of faculty requested change in hospital formulary [68].

*Conference travel*

Pharmaceutical company sponsored conference travels to touristic locations have quantifiable impact on the prescribing rational of attendees. A significant increase (three times) in the prescribing rate of two company drugs was observed after the physicians attended a company sponsored symposium with all their expenses covered. Despite this significant difference in the prescribing patterns, physicians insisted there was no impact on their prescribing behaviour [57].

*Industry paid lunches*

Most physicians received invitations for dinners [9] and free lunches [10, 21, 35, 43]. Clerks, interns and junior residents attended more company sponsored lunches than senior residents [10]. Pharmaceutical companies also sponsored departmental lunches during journal clubs [39]. There was no significant association between attending industry paid lunches [37] and dinners [9] and formulary request for that company's drug (Table 2). However, there was a significant

For peer review only

association between attending industry paid lunches and increased prescription of branded drugs [52, 53, 69].

*CME sponsorship*

Physicians who attended company sponsored CME events had more positive attitudes towards and inclination to prescribe the branded drugs [28, 34, 43, 67, 70]. We found that physicians who refused CME sponsorship were seen to prescribe higher proportion of generics and lower expenditure medicines when compared to physicians who attended CMEs [22].

## Discussion

We report that there is widespread interaction between the pharmaceutical industry and physicians [9-11, 13, 42, 50]. Interactions are in the form of personal communications, free gifts such as drug samples, sponsored meals, sponsored conference travel, funding for research and CMEs and honoraria [9, 21, 22, 31, 36, 42]. The frequency of these interactions is comparable between residents and physicians [10, 21, 22, 36, 42]. However, the amount and type of gifts vary with the position of the physician in medical hierarchy, specialization and location of practice [10, 13, 31, 38, 42, 43, 50, 58, 62]. In general, trainees (residents, interns) are treated with more drug samples, stationery items and free meals than senior physicians [10, 13]. Senior physicians usually avail of sponsored conferences/ trips, research funding, honoraria and CME events. The extent of these interactions varies with academic versus non-academic institutions: non-academic hospitals record more interactions than others [31, 38, 42, 50, 55]. The majority of the physicians do not believe that they are affected by PSR interactions [1, 10, 32, 33, 35, 37, 43, 59]. However, a sizeable percentage in various surveys responded in the affirmative when asked whether they thought that their peers are vulnerable [1, 20, 21, 37, 43]. It is further noted that there is a trend towards non-physician clinicians interactions and prescribing, such as nurses whom, also, generally hold a positive attitude toward PSR-interactions. This further depicts the extent of interactions and also might expose a risk of replicating irrational prescribing in non-physicians [71].

We observe that there is a positive correlation between acceptance of gifts and physicians' urge to reciprocate favorably towards the benefactor [12, 22, 47]. Considering that physicians have a social contract with society at large to provide unbiased and altruistic service and also the impact of these interactions on healthcare costs, this is an alarming observation. Countries have put into effect legislation and policies to curb activities that abuse the role of physicians

For peer review only

as gatekeepers of society's health, such as the The Sunshine Act which is the first Congressional involvement in regulating the disclosure by physicians of payments by pharmaceutical companies and manufacturers of drugs, federal health care programmes are required to report certain payments and items of value given to physicians and teaching hospitals (e.g. speaking fees, consulting arrangements, and free food) [72-74]. The purpose is to prevent undue influence and protect the public interest. Moreover, a 2005 joint report by the WHO and Health Action International (HAI) reported on interventions to counter promotional activities [75]. The evidence presented in that report was not eligible for our systematic review, mostly because it related to interventions on students or doctors-in-training. Nevertheless, the findings suggested that interventions such as industry self-regulation and guidelines for sales representatives are not effective, while education about drug promotion might influence physician attitudes [76-78].

*Policies and educational intervention*

The relationship of physicians with patients is of a fiduciary nature. Hence activities that might affect that relationship by altering physicians' clinical behavior are not acceptable. Physician-PSR interactions may put the trust of patients in physicians at risk. Interaction with PSRs begins at medical school. Trainees are exposed to PSR marketing and promotional techniques from the initial years of their medical education, which impact their prescribing behavior in future. Overall, trainees, i.e., residents and interns, are more vulnerable to PSR interactions than senior physicians [11, 41, 62]. Physicians are susceptible to PSR interactions, which influences their clinical decision-making leading to greater prescriptions of branded drugs over low cost generic medicines and increasing healthcare costs [22, 47, 52, 53, 69]. In addition, this is accompanied by requests to add the benefactor company's drug to existing hospital formulary [61]. Therefore, there is need to institute and implement stringent policies curtailing physician-PSR relationships, as well as educational programs to increase awareness. Previous reports have indicated that implementing policies and conducting educational programs are effective in increasing awareness of physician's attitudes towards PSR interactions [54, 59, 60, 76, 79-83].

*Strengths and Limitations of the study*

A major strength of this study is that is a large up-to-date systematic review of studies exploring the effects of physician and pharmaceutical industry representative interactions and their impact on attitudes, knowledge and prescribing behavior of practicing physicians and

For peer review only

residents in different settings (e.g. academic, primary care). Another strength of this study is the use of Cochrane and GRADE methodologies for conducting a review and assessing the quality of the studies. Moreover, we performed an extensive search in 3 databases and the grey literature. Some of the limitations of this review are related to the included studies, as some did not provide evidence for the significance of their findings or had varying study designs and outcomes, which made it impossible to conduct a meta-analysis. Also, the included studies were subject to risk of bias related to the lack of validity of outcome measurement, and inadequate handling of significant potential confounders. Another limitation is that our search was limited from January 1992 to August 2016.

*Future implications*

PSR interactions compromise the objectivity of the physicians and are likely to result in irrational prescribing behavior and increasing healthcare costs. Educating physicians and increasing regulation of PSR interactions may lower the likelihood of prescribing new non-superior industry drugs and irrational prescription behavior. Further studies are required to evaluate the benefits of various intervention based education programs on the clinical and ethical behavior of the physicians.

For peer review only

## References


1. Steinman, M.A., M.G. Shlipak, and S.J. McPhee, *Of principles and pens: attitudes and practices of medicine housestaff toward pharmaceutical industry promotions.* The American Journal of Medicine, 2001. **110**(7): p. 551-557.
2. Wazana, A., *Physicians and the Pharmaceutical Industry.* JAMA, 2000. **283**(3): p. 373.
3. Wolfe, S.M., *Why do american drug companies spend more than $12 billion a year pushing drugs? Is it education or promotion?* Journal of General Internal Medicine, 1996. **11**(10): p. 637-639.
4. Wall, L.L. and D. Brown, *The High Cost of Free Lunch.* Obstetrics & Gynecology, 2007. **110**(1): p. 169-173.
5. Chressanthis, G.A., et al., *Determinants of pharmaceutical sales representative access limits to physicians.* Journal of Medical Marketing, 2014. **14**(4): p. 220-243.
6. International, K., *Rising costs of litigation in pharmaceutical industry.* Issues Monitor-Pharmaceuticals, 2011. **9**(14): p. 1-3.
7. MedAdNews, *Table insert on the top 50 pharma companies. Data provided by Cegedim Strategic Data.* 2010. **September**.
8. Mack J, *Pharma promotional spending in 2013.* Pharma marketing news, 2014. **13**(5).
9. De Ferrari, A., et al., *Attitudes and Relationship between Physicians and the Pharmaceutical Industry in a Public General Hospital in Lima, Peru.* PLoS ONE, 2014. **9**(6): p. e100114.
10. Hodges B. *Interactions with the pharmaceutical industry: experiences and attitudes of psychiatry residents, interns and clerks.* CMAJ: Canadian Medial Association Journal, 1995. **153**(5): p. 553.
11. Riese, F., et al., *Pharmaceutical industry interactions of psychiatric trainees from 20 European countries.* European Psychiatry, 2015. **30**(2): p. 284-290.
12. Lee, D. and C.E. Begley, *Physician report of industry gifts and quality of care.* Health Care Management Review, 2016. **41**(3): p. 275-283.
13. Montastruc, F., et al., *Interactions between Medical Residents and Drug Companies: A National Survey after the Mediator® Affair.* PLoS ONE, 2014. **9**(10): p. e104828.
14. Fugh-Berman, A. and S. Ahari, *Following the Script: How Drug Reps Make Friends and Influence Doctors.* PLoS Medicine, 2007. **4**(4): p. e150.
15. Kesselheim, A.S., M.M. Mello, and D.M. Studdert, *Strategies and Practices in Off-Label Marketing of Pharmaceuticals: A Retrospective Analysis of Whistleblower Complaints.* PLoS Medicine, 2011. **8**(4): p. e1000431.
16. Mulinari, S., *Unhealthy marketing of pharmaceutical products: An international public health concern.* Journal of Public Health Policy, 2016. **37**(2): p. 149-159.
17. Othman, N., et al., *Medicines information provided by pharmaceutical representatives: a comparative study in Australia and Malaysia.* BMC Public Health, 2010. **10**(1).
18. Ziegler, M.G., *The Accuracy of Drug Information From Pharmaceutical Sales Representatives.* JAMA: The Journal of the American Medical Association, 1995. **273**(16): p. 1296.
19. Alosaimi FD, A.K.A., Qadi M, Albahlal A, Alabdulkarim Y, Alabduljabbar M, Alqahtani F., *Physicians' attitudes towards interaction with the pharmaceutical industry/Attitudes des médecins par rapport á l'interaction avec l'industrie pharmaceutique.* Eastern Mediterranean Health Journal, 2014. **20**(12): p. 812.


For peer review only

20. Andaleeb, S.S. and R.F. Tallman, *Physician attitudes toward pharmaceutical sales representatives.* Health Care Management Review, 1995. **20**(3): p. 68-76.
21. Lieb K, B.S., *A survey of German physicians in private practice about contacts with pharmaceutical sales representatives.* Dtsch Arztebl Int., 2012. **107**(22): p. 392-8.
22. Lieb, K. and A. Scheurich, *Contact between Doctors and the Pharmaceutical Industry, Their Perceptions, and the Effects on Prescribing Habits.* PLoS ONE, 2014. **9**(10): p. e110130.
23. Francer, J., et al., *Ethical pharmaceutical promotion and communications worldwide: codes and regulations.* Philosophy, Ethics, and Humanities in Medicine, 2014. **9**(1): p. 7.
24. Canada., C.o.f.p.o.C.R.c.o.p.a.s.o., *Guide tot enhancing referrals and consultations between physicians.* College of Family Physicians of Canada., 2009.
25. Grande, D., *Limiting the Influence of Pharmaceutical Industry Gifts on Physicians: Self-Regulation or Government Intervention?* Journal of General Internal Medicine, 2009. **25**(1): p. 79-83.
26. Brennan, T.A., et al., *Health Industry Practices That Create Conflicts of Interest.* JAMA, 2006. **295**(4): p. 429.
27. Spurling, G.K., et al., *Information from Pharmaceutical Companies and the Quality, Quantity, and Cost of Physicians' Prescribing: A Systematic Review.* PLoS Medicine, 2010. **7**(10): p. e1000352.
28. J., Lexchin., *Interactions between physicians and the pharmaceutical industry: what does the literature say?* . CMAJ: Canadian Medial Association Journal, 1993. **14**(10): p. 1401-7.
29. Wazana, A., et al., *Using the Literature in Developing McGill's Guidelines for Interactions between Residents and the Pharmaceutical Industry.* Academic Medicine, 2004. **79**(11): p. 1033-1040.
30. Guyatt, G., et al., *GRADE guidelines: 1. Introduction—GRADE evidence profiles and summary of findings tables.* Journal of Clinical Epidemiology, 2011. **64**(4): p. 383-394.
31. Thomson AN, C.B., Barham PM., *Attitudes of general practitioners in New Zealand to pharmaceutical representatives.* Br J Gen Pract., 1994. **44**(382): p. 220-3.
32. Kamal, S., et al., *Perceptions and Attitudes of Egyptian Health Professionals and Policy-Makers towards Pharmaceutical Sales Representatives and Other Promotional Activities.* PLOS ONE, 2015. **10**(10): p. e0140457.
33. Gibbons, R.V., et al., *A comparison of physicians' and patients' attitudes toward pharmaceutical industry gifts.* Journal of General Internal Medicine, 1998. **13**(3): p. 151-154.
34. Scheffer, M.C., *Interaction between pharmaceutical companies and physicians who prescribe antiretroviral drugs for treating AIDS.* Sao Paulo Medical Journal, 2014. **132**(1): p. 55-60.
35. Brett, A.S., W. Burr, and J. Moloo, *Are Gifts From Pharmaceutical Companies Ethically Problematic?* Archives of Internal Medicine, 2003. **163**(18): p. 2213.
36. Gupta, S., R. Nayak, and R. Sivaranjani, *A study on the interactions of doctors with medical representatives of pharmaceutical companies in a Tertiary Care Teaching Hospital of South India.* Journal of Pharmacy and Bioallied Sciences, 2016. **8**(1): p. 47.
37. Morgan, M.A., *Interactions of doctors with the pharmaceutical industry.* Journal of Medical Ethics, 2006. **32**(10): p. 559-563.
38. Caudill, T.S., *Physicians, pharmaceutical sales representatives, and the cost of prescribing.* Archives of Family Medicine, 1996. **5**(4): p. 201-206.

For peer review only

39. Reeder, M., J. Dougherty, and L.J. White, *Pharmaceutical representatives and emergency medicine residents: A national survey.* Annals of Emergency Medicine, 1993. **22**(10): p. 1593-1596.
40. Lichstein PR, T.R., O'Brien K., *Impact of pharmaceutical company representatives on internal medicine residency programs: a survey of residency program directors.* Archives of Internal Medicine, 1992. **152**(5): p. 1009-13.
41. Brotzman GL, M.D., *Policies regulating the activities of pharmaceutical representatives in residency programs.* Journal of Family Practice, 1992. **34**(1): p. 54-8.
42. Alssageer, M. and S. Kowalski, *A survey of pharmaceutical company representative interactions with doctors in Libya.* Libyan Journal of Medicine, 2012. **7**(1): p. 18556.
43. Lieb K, K.C., *Medical students' attitudes to and contact with the pharmaceutical industry: a survey at eight German University hopitals.* Dtsch. Arztebl. Int., 2013. **110**(35): p. 584-90.
44. Brown SR, E.D., Berman AF., *Pharmaceutical industry interactions in family medicine residencies decreased between 2008 and 2013: a CERA study.* Fam. Med., 2015. **47**(4): p. 279-82.
45. Rahmana MH, A.S., Tuddenhama S, Petoa H, Iqbalb M, Bhuiyab A, Peters DH., *What do they do? Interactions between village doctors and medical representatives in Chakaria, Banladesh.* Int. Health., 2015. **7**(1): p. 266-71.
46. KlemencKetis, Z. and J. Kersnik, *Which Pharmaceutical Sales Representatives' Features do Slovenian Family Physicians Value?* Acta Informatica Medica, 2013. **21**(4): p. 257.
47. Hurley, M.P., R.S. Stafford, and A.T. Lane, *Characterizing the Relationship Between Free Drug Samples and Prescription Patterns for Acne Vulgaris and Rosacea.* JAMA Dermatology, 2014. **150**(5): p. 487.
48. Makowska, M., *Interactions between Doctors and Pharmaceutical Sales Representatives in a Former Communist Country.* Cambridge Quarterly of Healthcare Ethics, 2014. **23**(03): p. 349-355.
49. Siddiqui, U.T., et al., *Attitudes of medical students towards incentives offered by pharmaceutical companies- perspective from a developing nation- a cross sectional study.* BMC Medical Ethics, 2014. **15**(1).
50. Workneh, B.D., et al., *Influence of Medical Representatives on Prescribing Practices in Mekelle, Northern Ethiopia.* PLOS ONE, 2016. **11**(6): p. e0156795.
51. Khan, N., et al., *Perceptions and Attitudes of Medical Sales Representatives (MSRs) and Prescribers Regarding Pharmaceutical Sales Promotion and Prescribing Practices in Pakistan.* Journal of Young Pharmacists, 2016. **8**(3): p. 244-250.
52. DeJong, C., et al., *Pharmaceutical Industry–Sponsored Meals and Physician Prescribing Patterns for Medicare Beneficiaries.* JAMA Internal Medicine, 2016. **176**(8): p. 1114.
53. Yeh, J.S., et al., *Association of Industry Payments to Physicians With the Prescribing of Brand-name Statins in Massachusetts.* JAMA Internal Medicine, 2016. **176**(6): p. 763.
54. Yeh, J.S., et al., *Association of Medical Students' Reports of Interactions with the Pharmaceutical and Medical Device Industries and Medical School Policies and Characteristics: A Cross-Sectional Study.* PLoS Medicine, 2014. **11**(10): p. e1001743.
55. Parikh, K., W. Fleischman, and S. Agrawal, *Industry Relationships With Pediatricians: Findings From the Open Payments Sunshine Act.* PEDIATRICS, 2016. **137**(6): p. e20154440-e20154440.

For peer review only

56. Spingarn, R.W., J.A. Berlin, and B.L. Strom, *When pharmaceutical manufacturers' employees present grand rounds, what do residents remember?* Academic Medicine, 1996. **71**(1): p. 86-8.

57. Orlowski, J.P. and L. Wateska, *The Effects of Pharmaceutical Firm Enticements on Physician Prescribing Patterns: there's no such thing as a free lunch.* Chest, 1992. **102**(1): p. 270-273.

58. Zaki, N.M., *Pharmacists' and physicians' perception and exposure to drug promotion: A Saudi study.* Saudi Pharmaceutical Journal, 2014. **22**(6): p. 528-536.

59. Randall, M., Rosenbaum JR, Rohrbaugh RM, Rosenheck RA., *Attitudes and Behaviors of Psychiatry Residents Toward Pharmaceutical Representatives Before and After an Educational Intervention.* Academic Psychiatry, 2005. **29**(1): p. 33-39.

60. Esmaily, H.M., et al., *Can rational prescribing be improved by an outcome-based educational approach? A randomized trial completed in Iran.* Journal of Continuing Education in the Health Professions, 2010. **30**(1): p. 11-18.

61. Chren MM, L.C., *Physicians' Behavior and Their Interactions With Drug Companies: a controlled study of physicians who requested additions to a hospital drug formulary.* JAMA, 1994. **271**(9): p. 684.

62. Zipkin, D.A. and M.A. Steinman, *Interactions between pharmaceutical representatives and doctors in training.* Journal of General Internal Medicine, 2005. **20**(8): p. 777-786.

63. McMurray, R.J., *Gifts to Physicians From Industry.* JAMA: The Journal of the American Medical Association, 1991. **265**(4): p. 501.

64. Lotfi, T., et al., *Knowledge, beliefs and attitudes of physicians in low and middle-income countries regarding interacting with pharmaceutical companies: a systematic review.* BMC Health Services Research, 2016. **16**(1).

65. Fischer, M.A., et al., *Prescribers and Pharmaceutical Representatives: Why Are We Still Meeting?* Journal of General Internal Medicine, 2009. **24**(7): p. 795-801.

66. Chimonas, S., T.A. Brennan, and D.J. Rothman, *Physicians and Drug Representatives: Exploring the Dynamics of the Relationship.* Journal of General Internal Medicine, 2007. **22**(2): p. 184-190.

67. Saito, S., K. Mukohara, and S. Bito, *Japanese Practicing Physicians' Relationships with Pharmaceutical Representatives: A National Survey.* PLoS ONE, 2010. **5**(8): p. e12193.

68. Lurie, N., et al., *Pharmaceutical representatives in academic medical centers.* Journal of General Internal Medicine, 1990. **5**(3): p. 240-243.

69. Steinbrook, R., *Industry Payments to Physicians and Prescribing of Brand-name Drugs.* JAMA Internal Medicine, 2016. **176**(8): p. 1123.

70. Bowman, M.A. and D.L. Pearle, *Changes in drug prescribing patterns related to commercial company funding of continuing medical education.* Journal of Continuing Education in the Health Professions, 1988. **8**(1): p. 13-20.

71. Grundy Q, B.L., Malone R 2013, *Interactions between non-physician clinicians and industry: a systematic review.* PLoS Med. , 2013. **11**(Nov 26).

72. Dixon, D., et al., *Controlling Quality in CME/CPD by Measuring and Illuminating Bias.* Journal of Continuing Education in the Health Professions, 2011. **31**(2): p. 109-116.

73. Silverman, E., *Everything you need to know about the Sunshine Act.* BMJ, 2013. **347**(jul26 1): p. f4704-f4704.

74. Shalowitz, D.I., M.A. Spillman, and M.A. Morgan, *Interactions with industry under the Sunshine Act: an example from gynecologic oncology.* American Journal of Obstetrics and Gynecology, 2016. **214**(6): p. 703-707.

For peer review only

75. Norris P, H.A., Lexchin J, et al., *Drug promotion—what we know, what we have yet to learn—reviews of materials in the WHO/HAI database on drug promotion.* EDM Research Series, 2005(No. 032.).
76. Carroll, A.E., et al., *To What Extent Do Educational Interventions Impact Medical Trainees' Attitudes and Behaviors Regarding Industry-Trainee and Industry-Physician Relationships?* PEDIATRICS, 2007. **120**(6): p. e1528-e1535.
77. Birkhahn, R.H., et al., *A Review of the Federal Guidelines That Inform and Influence Relationships Between Physicians and Industry.* Academic Emergency Medicine, 2009. **16**(8): p. 776-781.
78. Chressanthis, G.A., et al., *Can Access Limits on Sales Representatives to Physicians Affect Clinical Prescription Decisions? A Study of Recent Events With Diabetes and Lipid Drugs.* The Journal of Clinical Hypertension, 2012. **14**(7): p. 435-446.
79. Sergeant MD, H.P., Godwin M, Walker DM, McHenry P., *Interactions with the pharmaceutical industry: a survey of family medicine residents in Ontario.* CMAJ: Canadian Medial Association Journal, 1996. **155**(9): p. 1243.
80. T Montague, B., A.H. Fortin Vi, and J. Rosenbaum, *A systematic review of curricula on relationships between residents and the pharmaceutical industry.* Medical Education, 2008. **42**(3): p. 301-308.
81. Larkin, I., et al., *Restrictions On Pharmaceutical Detailing Reduced Off-Label Prescribing Of Antidepressants And Antipsychotics In Children.* Health Affairs, 2014. **33**(6): p. 1014-1023.
82. R., P., *Guidelines for faculty involvement in commercially supported continuing medical-education.* Academic Medicine, 1992.
83. Greenland, P., *Time for the Medical Profession to Act.* Archives of Internal Medicine, 2009. **169**(9): p. 829.

For peer review only

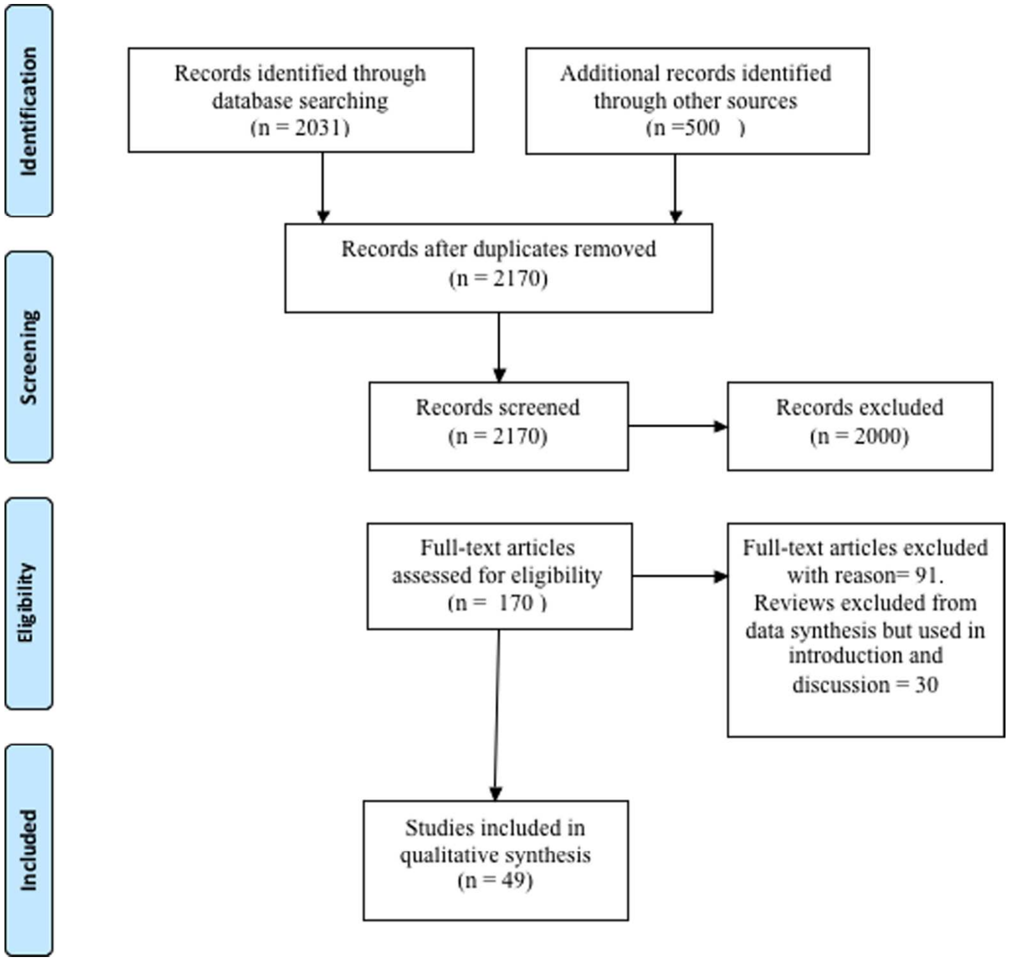


Flow diagram of study selection.

| Study | Authors | Country | Participants, setting | Study design | Interaction with | Outcomes |
|---|---|---|---|---|---|---|
| 1 | Steinman et al., 2001 | USA | Surveys about attitudes and behaviors toward industry gifts in 105 residents at a university-based internal medicine residency program | Cross-sectional | PSR interactions, gifts | Most participants (61%) hold positive attitudes toward gifts from industry and PSR interactions and believe they do not influence their own prescribing, but only 16% believed other physicians were similarly unaffected (P< .0001) |
| 11 | De Ferrari et al., 2014 | Peru | Questionnaire in 155 faculty and trainee physicians of five different clinical departments working in a public general hospital | Cross-sectional | PSR interactions, medical samples, promotional material, dinners | Positive attitude towards representatives (88.5% of participants). Faculty physicians received a larger amount of medical samples and promotional material and were more prone to believe that gifts and lunches do not influence their prescribing behavior (42.2% vs. 23.6%; p=0.036) |
| 12 | Thomson et | New | Questionnaire | Cross- | Interactions with | Most general |

For peer review only

| | | | | | | |
|---|---|---|---|---|---|---|
| | al., 1994 | Zealand | survey of 67 general practitioners | sectional | PSR | practitioners (67%) had a negative attitude toward PSR interaction |
| 13 | Kamal et al., 2015 | Egypt | Interviews with 18 physicians | Cross-sectional | Interaction with PSR | Positive attitude towards PSR interaction |
| 14 | Hodges, 1995 | Canada | Survey in 105 residents of psychiatry | Cross-sectional | Interaction with PSR, drug samples, lunches | Positive attitude towards PSR interaction (56.5% of participants). The more money and promotional items a participant had received, the more likely he or she was to believe that discussions with representatives did not affect prescribing ($p < 0.05$) |
| 15 | Gibbons et al., 1998 | USA | Survey of 392 physicians in two tertiary-care medical centers | Cross-sectional | PSR interactions,gifts, samples, travel, lunches | Positive attitude towards PSR interactions, gifts, samples and lunches |
| 16 | Spingarn et al., 1996 | USA | 75 internal medicine physicians in university medical center | Retrospective cohort | PSR interaction (teaching) | Attendees inappropriately prescribed PSR speakers drug compared to non-attendees ($p=0.029$) |
| 17 | Zaki, 2014 | Saudi Arabia | Survey of 250 physicians | Randomized, cross-sectional survey | Conferences, drug samples | Favorable towards promotion |
| 18 | Orlowski et | USA | 10 physicians that | Cohort | Conference | Significant |

For peer review only

| | | | | | | |
|---|---|---|---|---|---|---|
| | al., 1994 | | were invited for a symposium and tracking the pharmacy inventory usage reports for these drugs before and after the symposia | | travel | increase in the prescribing pattern of drugs occurred following the symposia (p<0.001) |
| 19 | Scheffer et al., 2014 | Brazil | Survey of 300 physicians prescribing antiretroviral drugs | Cross-sectional | Interaction with representative, drug samples, journals | Frequency of interaction; the majority of (64%) of the physicians had multiple forms of interactions with PSR |
| 20 | Brett et al., 2003 | USA | Questionnaire of 93 physicians in a medical school | Cross-sectional | Interaction with PSR | Impact on attitudes; most physicians believed that most of PSR activities do not pose major ethical problems |
| 21 | Gupta et al., 2016 | India | Survey of 81 physicians in single hospital | Cross-sectional | Interaction with representative, drug samples, journals | Impact on prescribing; 61.7% of participants think that PSR has an impact on their prescribing (P = 0.0001) |
| 22 | Morgan et al., 2006 | USA | Survey of 397 obstetrician-gynaecologists | Cross-sectional | Drug samples, promotional material, lunch | Impact on prescribing, positive attitudes; most respondents thought it's proper to accept drug samples (92%), lunch (77%), an anatomical model (75%) or a well- |

For peer review only

| | | | | | | |
|---|---|---|---|---|---|---|
| | | | | | | paid consultantship (53%) from PSR |
| 23 | Alosaimi et al., 2014 | Saudi Arabia | Survey of 659 physicians | Cross-sectional | Interaction with PSR | Positive attitude towards PSR interaction |
| 24 | Chren et al., 1994 | USA | 40 case physicians and 80 control physicians | Case control | PSR interactions, honoraria, research | Increased prescription of company's drug after PSR interaction, honoraria and research ($p<0.001$, all) |
| 25 | Randall et al., 2005 | USA | Intervention group of physicians (n=18) that received education about PSR interaction and control group (n=14) | Controlled trial | Interaction with PSR | The majority of residents found the interactions and gifts useful. Compared to the comparison group, the intervention group significantly decreased the reported number of office supplies and noneducational gifts ($p<0.05$) |
| 26 | Caudil et al., 1996 | USA | Survey of 446 primary care physicians | Cross-sectional | Interaction with PSR | Significant positive correlation between physician cost of prescribing and perceived credibility, availability, applicability, and use of information |

For peer review only

| | | | | | | |
|---|---|---|---|---|---|---|
| | | | | | | provided by PSR ($p < 0.01$) |
| 27 | Andaleeb et al., 1995 | USA | 223 physicians in northwestern Pennsylvania | Cross-sectional | Interaction with PSR | Positive attitude towards PSR interaction |
| 28 | Reeder et al., 1993 | USA | 87 residents of emergency medicine | Cross-sectional | Interaction with PSR, gifts | Most participants believed that PSR interaction had no impact on their prescribing |
| 29 | Lichstein et al., 1992 | USA | 272 directors of internal medicine residency programs | Cross-sectional | Interaction with PSR | Most participants had a positive attitude towards PSR interactions |
| 30 | Brotzman et al., 1992 | USA | Directors of 386 family practice residency programs | Cross-sectional | Interaction with PSR | Majority of programs do not have guidelines for interaction with PSR |
| 31 | Alssageer et al., 2012 | Libya | Survey of 608 physicians in public and private practice settings | Cross-sectional | Interaction with PSR, drug samples, printed materials | Positive attitude towards PSR interactions |
| 32 | Lieb & Brandtonies, 2010 | German y | Survey of 208 physicians (neurology, cardiology and general medicine) | Cross-sectional | Interaction with PSR, drug samples, printed materials, lunches | Frequency and impact on attitudes |
| 33 | Lieb & Scheurich, 2014 | German y | Survey of 160 physicians in private and public practices | Cross-sectional | Interaction with representative, drug samples, printed materials, CME | High expenditure prescribing; avoidance of industry-sponsored CME is associated with more rational prescribing habits |
| 34 | Lieb & Koch, 2013 | German y | Survey of 1038 medical students at 8 universities | Cross-sectional | Interaction with representative, drug samples, | Most participants have contact |

For peer review only

| | | | | | | |
|---|---|---|---|---|---|---|
| | | | | | printed materials, lunches | with the pharmaceutical company; 24.6% of the participants thought gifts would influence their future prescribing behavior, while 45.1% thought gifts would influence their classmates' future prescribing behavior (p<0.001) |
| 35 | Brown et al., 2015 | USA | 251 directors of family medicine residency programs | Cross-sectional | Interaction with PSR, gifts, lunches | Negative attitude towards PSR interactions |
| 37 | Rahmana et al., 2015 | Bangladesh | Survey of 83 village physicians | Cross-sectional | Interaction with PSR | Impact on their prescribing |
| 38 | Lee & Begley, 2016 | USA | Nationally representative survey of 4720 physicians | Cross-sectional | Gifts | Gifts were associated with lower perceived quality of patient care; an inverse relationship between the frequency of received gifts and the perceived quality of care was observed |
| 39 | Montastruc et al., 2014 | France | Survey among 631 medical residents | Cross-sectional | Interaction with representative | Most participants believed that PSR interaction had no impact on their prescribing; participants |

| | | | | | | |
|---|---|---|---|---|---|---|
| | | | | | | who had a more positive opinion were more frequently exposed to PSR (p<0.001) |
| 40 | Ketis & Kersnik, 2013 | Slovenia | 895 family physicians at the primary level of care | Cross-sectional | Interaction with PSR | Positive effect on knowledge; Participants value PSRs' selling and communication skills and trustworthiness highly |
| 41 | Hurley et al., 2014 | USA | 3500 dermatologists | Cross-sectional | Free drug samples | Impact on their prescribing; the provision of samples with a prescription by dermatologists has been increasing over time, and this increase is correlated (r = 0.92) with the use of the branded generic drugs promoted by these sample |
| 42 | Makowska, 2014 | Poland | Survey of 382 physicians | Cross-sectional | Gifts | Positive attitude towards PSR interactions |
| 43 | Siddiqui et al., 2014 | Pakistan | Questionnaires of 352 medical students | Cross-sectional | Interaction with representative | Positive attitude towards PSR interaction |
| 55 | Workneh BD et al., 2016 | Ethiopia | Survey of 90 physicians from public and private health facilities | Cross-sectional | Interaction with representative, gifts | Positive attitude towards industry, impact on |

For peer review only

| | | | | | | |
|---|---|---|---|---|---|---|
| | | | | | | prescribing behavior; Nearly half of the physicians reported that their prescribing decisions were influenced by PSR |
| 57 | Khan N et al., 2016 | Pakistan | Questionnaires in 472 physicians | Cross-sectional | Interaction with representative, gifts | Positive attitude towards PSR interaction |
| 58 | Saito S et al. 2010 | Japan | 1417 physicians working in internal medicine, general surgery, orthopedic surgery, pediatrics, obstetrics-gynecology, psychiatry, and ophthalmology | National Survey | Interaction with industry, receipt of gifts, funds, CME, samples | Positive attitude towards PSR and gifts, value information from PSR, interactions higher with physicians who prefer to prescribe brand names |
| 59 | Ziegler MG et al. 1995 | USA | 27 physicians working in public and private hospitals | Survey | Accuracy of information provided by PSRs about drugs | Incorrect information often provided by speakers goes unnoticed by physicians |
| 60 | Lurie N et al., 1990 | USA | 240 internal medicine faculty physicians in academic medical centers | Survey | Effect of interaction with PSR, free meals, honoraria and research support | Impact on prescribing behavior and formulary change requests |
| 62 | DeJong C et al., 2016 | USA | 279.669 physicians who wrote Medicare prescriptions in any of 4 drug classes: statins, cardioselective β-blockers, angiotensin-converting enzyme inhibitors and angiotensin-receptor | Cross-sectional | Industry sponsored meals | Receipt of industry-sponsored meals was associated with an increased rate of brand name prescription. |

For peer review only

| | | | | | | |
|---|---|---|---|---|---|---|
| | | | blockers (ACE inhibitors and ARBs), and selective serotonin and serotonin-norepinephrine reuptake inhibitors (SSRIs and SNRIs) Physicians | | | |
| 63 | Yeh JS et al., 2016 | USA | All licensed Massachusetts physicians who wrote prescriptions for statins paid for under the Medicare drug benefit in 2011 (n=2444) | Cross-sectional | Effect of industry payment on prescription of branded drugs for cholesterol control | Payment for meals and educational programs increased prescription of brand name statins. |
| 65 | Bowman MA et al., 1988 | USA | 121 physician attendees | Self report survey | Effect of CME on prescribing behavior | Sponsoring company's drugs were favored during prescription |
| 66 | Fischer MA et al., 2009 | USA | Multi-disciplinary focus groups with 61 physicians | Survey | Effect of industry marketing strategies on prescription and cognitive dissonance of physicians | Most participants reported no PSR impact on their prescribing, value to have ability to evaluate information of PSRs |
| 67 | Chimonas S et al., 2007 | USA | Six focus groups in 32 academic and community physicians | Survey | PSR interactions | Positive attitude towards PSR interaction |
| 72 | Yeh JS et al., 2014 | USA | 1610 US medical students | Cross-sectional | Interaction with representative, gifts, lunches | Policies separating students from representatives reduced number of interactions |
| 73 | Larkin I et al., 2014 | USA | Pediatricians, child & adolescent psychiatrists in five medical centers | Survey | Interaction with PSR | Anti detailing policies reduced the prescription of off-label antidepressants and anti |

For peer review only

| | | | | | | |
|---|---|---|---|---|---|---|
| | | | | | | psychotics for children |
| 74 | Esmaily HM et al., 2010 | Iran | 112 general physicians were randomized in two groups: 1) outcome-based educational intervention for rational prescribing and 2) concurrent CME program in the field of rational prescribing | Randomized trial | Effect of outcome and retinal prescribing | Rational prescribing improved in some of the important outcome-based indicators. No difference between two arms of the study |
| 76 | Parikh K et al., 2016 | USA | descriptive, cross-sectional analysis of Open Payments data and 9 638 825 payments to physicians and pediatricians from January 1 to December 31, 2014 | Cross-sectional | Comparison of PSR interactions between pediatricians and other specialists; among subspecialities of pediatrics. | Pediatricians get fewer gifts from PSR than internists. There is variation among sub specialities for extent of interaction. |
| 78 | Chressanthis GA et al. 2012 | USA | Clinical decisions of 72,114 physicians were statistically analyzed using prescription data | Survey | Effect of restricting PSRs on clinical practice and knowledge | Restricting PSRs affected information flow about drugs, both negative and positive. |

For peer review only

| # | Attitudes | Prescribing behavior | Knowledge | Formulary requests | Quality of Evidence (GRADE) |
|---|---|---|---|---|---|
| **Gifts** | Receiving higher number of gifts associated with belief that PRs have no impact on their prescribing behaviour [1,10,13] | - | - | - | **Moderate** |
| **Drug samples** | Positive attitude towards the drug industry and the representatives [9,36,43] | Higher prescription of the company drug [36, 47] | - | - | **High** |
| **Pharmaceutical representative speakers** | - | Irrational prescribing [56, 57, 43] | Inability to identify false claims[56] | Increased prescription of sponsor's drug[61] | **High** |
| **Honoraria and Research Funding** | Positive attitude towards sponsor's drug [68] | - | - | Increased prescription of sponsor's drug[61] | **Low** |
| **Conference travel** | - | Significant increase in prescribing of sponsor drug[57] | - | Increased prescription of sponsor's drug[61] | **Low** |
| **Industry paid lunches** | Positive attitude towards sponsor's drug [10, 43] | Significant increase in prescribing of sponsor drug[52] | - | Increased formulary request for company drug [9,36] | **High** |
| **CME sponsorship** | Positive attitude towards sponsor's drug [61, 70] | Avoidance of industry-sponsored CME associated with more rational prescribing habits [22] | | | **Moderate** |

For peer review only

| | | | | | |
|---|---|---|---|---|---|
| **Interaction with PRS** | Positive attitude towards PSR drugs [1,9,10,67] | Higher prescription of the company drug[61] | Positive correlation between the physicians' prescribing cost and the information provided by the drug representative during the interaction[38] | Increased prescription of sponsor's drug[61] | **High** |

For peer review only

Search strategy for PubMed search engine of Medline

In an attempt to find all related literature on the topic, studies related to physician-pharmaceutical representative interactions that affect the prescribing behavior of the physicians were identified through computerized searches using, but not limited to, the following subject headings and text words in PubMed from 1992 to 2016.

1. Physician interactions with pharmaceutical industry
2. Physician attitude towards pharmaceutical representatives
3. Behavior of physicians towards pharmaceutical representatives
4. Gifts AND physician AND pharmaceutical representatives
5. Honoraria AND physician AND pharmaceutical representatives
6. Continuing medical education AND physician AND pharmaceutical representatives
7. Research funding AND physician AND pharmaceutical representatives
8. Conference travel AND physician AND pharmaceutical representatives
9. Industry sponsored meals AND physician behavior

For peer review only

# PRISMA 2009 Checklist

| Section/topic | # | Checklist item | Reported on page # |
|---|---|---|---|
| **TITLE** | | | |
| Title | 1 | Identify the report as a systematic review, meta-analysis, or both. | 1 |
| **ABSTRACT** | | | |
| Structured summary | 2 | Provide a structured summary including, as applicable: background; objectives; data sources; study eligibility criteria, participants, and interventions; study appraisal and synthesis methods; results; limitations; conclusions and implications of key findings; systematic review registration number. | 2 |
| **INTRODUCTION** | | | |
| Rationale | 3 | Describe the rationale for the review in the context of what is already known. | 3 |
| Objectives | 4 | Provide an explicit statement of questions being addressed with reference to participants, interventions, comparisons, outcomes, and study design (PICOS). | 3 |
| **METHODS** | | | |
| Protocol and registration | 5 | Indicate if a review protocol exists, if and where it can be accessed (e.g., Web address), and, if available, provide registration information including registration number. | 4 |
| Eligibility criteria | 6 | Specify study characteristics (e.g., PICOS, length of follow-up) and report characteristics (e.g., years considered, language, publication status) used as criteria for eligibility, giving rationale. | 3 |
| Information sources | 7 | Describe all information sources (e.g., databases with dates of coverage, contact with study authors to identify additional studies) in the search and date last searched. | 4 |
| Search | 8 | Present full electronic search strategy for at least one database, including any limits used, such that it could be repeated. | 4 |
| Study selection | 9 | State the process for selecting studies (i.e., screening, eligibility, included in systematic review, and, if applicable, included in the meta-analysis). | 3/4 |
| Data collection process | 10 | Describe method of data extraction from reports (e.g., piloted forms, independently, in duplicate) and any processes for obtaining and confirming data from investigators. | 4 |
| Data items | 11 | List and define all variables for which data were sought (e.g., PICOS, funding sources) and any assumptions and simplifications made. | 4 |
| Risk of bias in individual studies | 12 | Describe methods used for assessing risk of bias of individual studies (including specification of whether this was done at the study or outcome level), and how this information is to be used in any data synthesis. | 3 |
| Summary measures | 13 | State the principal summary measures (e.g., risk ratio, difference in means). | 3 |
| Synthesis of results | 14 | Describe the methods of handling data and combining results of studies, if done, including measures of consistency (e.g., $I^2$) for each meta-analysis. | 3 |

For peer review only - http://bmjopen.bmj.com/site/about/guidelines.xhtml

# PRISMA 2009 Checklist

| Section/topic | # | Checklist item | Reported on page # |
|---|---|---|---|
| Risk of bias across studies | 15 | Specify any assessment of risk of bias that may affect the cumulative evidence (e.g., publication bias, selective reporting within studies). | 3 |
| Additional analyses | 16 | Describe methods of additional analyses (e.g., sensitivity or subgroup analyses, meta-regression), if done, indicating which were pre-specified. | 3 |
| **RESULTS** | | | |
| Study selection | 17 | Give numbers of studies screened, assessed for eligibility, and included in the review, with reasons for exclusions at each stage, ideally with a flow diagram. | 5 |
| Study characteristics | 18 | For each study, present characteristics for which data were extracted (e.g., study size, PICOS, follow-up period) and provide the citations. | 5-8 |
| Risk of bias within studies | 19 | Present data on risk of bias of each study and, if available, any outcome level assessment (see item 12). | 5-8 |
| Results of individual studies | 20 | For all outcomes considered (benefits or harms), present, for each study: (a) simple summary data for each intervention group (b) effect estimates and confidence intervals, ideally with a forest plot. | 5-8 |
| Synthesis of results | 21 | Present results of each meta-analysis done, including confidence intervals and measures of consistency. | NA |
| Risk of bias across studies | 22 | Present results of any assessment of risk of bias across studies (see Item 15). | 5-8 |
| Additional analysis | 23 | Give results of additional analyses, if done (e.g., sensitivity or subgroup analyses, meta-regression [see Item 16]). | NA |
| **DISCUSSION** | | | |
| Summary of evidence | 24 | Summarize the main findings including the strength of evidence for each main outcome; consider their relevance to key groups (e.g., healthcare providers, users, and policy makers). | 8-9 |
| Limitations | 25 | Discuss limitations at study and outcome level (e.g., risk of bias), and at review-level (e.g., incomplete retrieval of identified research, reporting bias). | 9 |
| Conclusions | 26 | Provide a general interpretation of the results in the context of other evidence, and implications for future research. | 9 |
| **FUNDING** | | | |
| Funding | 27 | Describe sources of funding for the systematic review and other support (e.g., supply of data); role of funders for the systematic review. | 1 |

*From:* Moher D, Liberati A, Tetzlaff J, Altman DG, The PRISMA Group (2009). Preferred Reporting Items for Systematic Reviews and Meta-Analyses: The PRISMA Statement. PLoS Med 6(6): e1000097. doi:10.1371/journal.pmed1000097

For more information, visit: www.prisma-statement.org.



For peer review only - http://bmjopen.bmj.com/site/about/guidelines.xhtml

# Association between pharmaceutical sales representatives' interaction on physicians' attitudes and prescribing habits: A systematic review


| | |
|---|---|
| Journal: | *BMJ Open* |
| Manuscript ID | bmjopen-2017-016408.R2 |
| Article Type: | Research |
| Date Submitted by the Author: | 28-Jun-2017 |
| Complete List of Authors: | Fickweiler, Freek; Crowd for Cure<br>Fickweiler, Ward; Crowd for Cure,<br>Urbach, Ewout; Crowd for Cure |
| <b>Primary Subject Heading</b>: | Health policy |
| Secondary Subject Heading: | Patient-centred medicine |
| Keywords: | Change management < HEALTH SERVICES ADMINISTRATION & MANAGEMENT, Health policy < HEALTH SERVICES ADMINISTRATION & MANAGEMENT, Protocols & guidelines < HEALTH SERVICES ADMINISTRATION & MANAGEMENT |
| | |


For peer review only

# Association between pharmaceutical sales representatives' interaction on physicians' attitudes and prescribing habits: A systematic review

**Fickweiler F[1], Fickweiler W[1], Urbach E[1],**

**1: Crowd for Cure, Jacob van Ruysdaelstraat 34, 9718 SG Groningen, the Netherlands**

**Corresponding author's email address: freek@crowdforcure.com**

For peer review only

**All authors have completed the ICMJE uniform disclosure form at http://www.icmje.org/coi_disclosure.pdf and declare: no support from any organisation for the submitted work; no financial relationships with any organisations that might have an interest in the submitted work in the previous three years, no other relationships or activities that could appear to have influenced the submitted work**

**Contributor Statement: All authors have contributed equally and have substantial contributions to the conception or design of the work; Author Freek Fickweiler for the acquisition, analysis, and interpretation of data for the work; Author Freek Fickweiler for drafting the work and Authors Ewout Urbach and Ward Fickweiler for revising it critically for important intellectual content; and all authors (Freek Fickweiler, Ward Fickweiler and Ewout Urbach) contributed to final approval of the version to be published and agreed to be accountable for all aspects of the work in ensuring that questions related to the accuracy or integrity of any part of the work are appropriately investigated and resolved.**

**Competing interest: no financial relationships with any organisations that might have an interest in the submitted work in the previous three years, no other relationships or activities that could appear to have influenced the submitted work**

**Funding: all authors declare no support from any organisation for the submitted work.**

**Data sharing statement: any data relevant to a published article will be made available alongside the article when published.**

## Abstract

### Objectives

The objective of this review is to explore the frequency of physician and pharmaceutical industry interactions andits impact on physicians' attitude, knowledge and behavior.

### Data Sources

Pubmed, Embase, Cochrane library and Google scholar electronic databases were searched from 1992 to August 2016 using free text words and medical subject headings relevant to the topic.

### Study Selection

Studies included cross sectional studies, cohort studies, randomized trials and survey designs. Studies with narrative reviews, case reports, opinion polls, letters to the editor, systematic reviews and non-English studies were excluded from data synthesis.

### Data Extraction

Two reviewers independently extracted the data. Data on study design, study year, country, participant characteristics, setting, and number of participants were collected.

### Data Synthesis

Pharmaceutical sales representative (PSR) interactions influences the physicians' attitudes towards the representatives, their prescribing behavior and increases the number of formulary addition requests for the company's drug.

### Conclusion

Physician-pharmaceutical sales representatives interactions and acceptance of gifts and favors from the company's pharmaceutical sales representatives have been found to affect the physicians' prescribing behavior and are likely to contribute to irrational prescription of the company's drug**.** Therefore, intervention in the form of policy implementation and education about the implications of these interactions are needed.


## Strengths and limitations of the study

- Large up-to-date systematic review of studies exploring the impact of pharmaceutical industry representative interactions on physicians
- This systematic review used the recommendations outlined in the Cochrane Handbook for conducting systematic reviews and the GRADE methodology to assess the quality of the evidence by outcome.

For peer review only

- Pubmed, Embase, Cochrane library and Google scholar electronic databases were searched from 1992, as well as grey literature.
- Most studies identified were observational and of varying methodological design
- Some studies did not provide evidence for the significance of their findings

**Keywords:** pharmaceutical sales representative; physicians, drug industry; brand prescriptions; conflict of interest ;physicians-industry interactions; pharmaceutical industry; attitude of health personnel; gifts to physicians; medical education; irrational prescriptions

For peer review only

## Introduction

The relationship between physicians and the pharmaceutical industry has evoked heated debate for many decades[1]. In 2012, the pharmaceutical industry spent $89.5 billion on physician-pharmaceutical sales representative (PSR) interactions whichaccounted for 60% of the global sales and marketing spending[2-8]. Previous reports have demonstrated that PSRs may influence prescribing behavior,[9-16]. However, the evidence determining whether or not PSR interactions influence physicians are divided and contradictory. Studies have indicated that physicians may be unable to distinguish between promotional information and scientific evidence[17-18]. Physicians on the other hand believe their colleagues are more susceptible to PSR marketing strategies than themselves [19-22]. The majority of the physicians do not believe that they are affected by PSR interactions. Most medical and governmental institutions have installed guidelines and self-regulatory and legislative checks to address this controversy[5,15,16, 23-26]. However, while administrative proposals for deregulatory reforms that would remove some governmental authority over the industry are increasing, scientific evidence rigorously examining this controversy are needed. This review addresses this controversy by critically and systemically evaluating the evidence on the impact of PSR interactions on physicians.

## Methodology

*Protocol*

We followed a detailed methodology that we described in our review protocol, which is available upon request to the corresponding author. Two independent reviewers assessed selected articles as per inclusion/exclusion criteria, shortlisted them for writing the review and cross-checked each other. The review followed the Preferred Reporting Items for Systematic Reviews and Meta-Analyses (PRISMA) guidelines (Appendix 1).

*Eligibility criteria*

The eligibility criteria were:

- Types of studies: cross sectional studies, cohort studies, randomized trials and survey designs comparing an intervention of interest on at least one facet of extent, impact on behavior and attitude.
- Types of participants: physicians, pharmaceutical representatives, physicians in training/residents. We did not consider medical students or other health professionals.

- Types of exposure: any type of interaction between physicians and the pharmaceutical industry where there is direct interaction with the physician, such as meeting with drug representatives, participating in pharmaceutical-sponsored CME event, receiving travel funding, free drug samples, industry-provided meals, gifts to the individual and active presentation of industry-related information to the physician
- Types of outcome: knowledge of physicians (e.g. accuracy of knowledge related to a specific medication), beliefs and/or attitudes of physicians regarding physician-industry interactions (e.g. perceived influence of information from the pharmaceutical company on their behavior), behavior of physicians (e.g. prescribing quantity/frequency)
- Type of control: no interaction.
- Exclusion criteria were: qualitative, ecological, econometric studies, editorials, letters to the editor, studies on medical students, small samples sizes, studies assessing non-targeted or indirect interactions (e.g. journal advertisement) and research funding.

We did not exclude studies based on risk of bias. We took risk of bias into account when grading the quality of evidence using GRADE approach.

*Search strategy*

The search strategy included Pubmed, Embase, Cochrane library and Google scholar electronic databases (January 1992 to August 2016). Databases were not searched before 1992, as these studies were already investigated in an earlier review[27]. The search combined terms for physicians and pharmaceutical, and included both free text words and medical subject heading relevant to the topic. We did not use a search filter. The supplementary information file provides the full details for one database. Additional search strategies included a search of the grey literature (theses and dissertations). Also, we reviewed the references lists of included and relevant papers.[27-29]

*Assessment of risk of bias in included studies*

Two reviewers assessed in duplicate and independently the risk of bias in each eligible study.Disagreements were resolved by discussion or adjudication by a third reviewer. . We used the recommendations outlined in the Cochrane Handbook to assess the risk of bias in randomized studies. We graded each potential source of bias and rated the studies as high, low or unclear risk of bias.

review only

*Data analysis and synthesis*

The information extracted from the selected studies included type of study, study design, type of PSR interaction and type of outcome. We did not conduct a meta-analysis due to the heterogeneity of study design, types of interventions, outcomes assessed, and outcome measures used. Instead, we summarized the data narratively. We assessed the quality of evidence by outcome using the GRADE methodology[30].

**Results**

We independently screened the titles and abstracts of the 2170 identified records for potential eligibility. Out of 2170, the full text of 49 eligible citations which matched the inclusion criteria were retrieved and used for qualitative assessment during the writing of the review (Figure 1, Table 1)

| Study | Authors | Country | Participants, setting | Study design | Interaction | Outcomes |
|---|---|---|---|---|---|---|
| 1 | Steinman et al., 2001 | USA | Surveys about attitudes and behaviors toward industry gifts in 105 residents at a university-based internal medicine residency program | Cross-sectional | PSR interactions, gifts | Most participants (61%) hold positive attitudes toward gifts from industry and PSR interactions and believe they do not influence their own prescribing, but only 16% believed other physicians were similarly unaffected (P< .0001) |
| 11 | De Ferrari et al., 2014 | Peru | Questionnaire in 155 faculty and trainee physicians of five different clinical departments working in a public | Cross-sectional | PSR interactions, medical samples, promotional material, dinners | Positive attitude towards representatives (88.5% of participants). |

For peer review only

| | | | | | | |
|---|---|---|---|---|---|---|
| | | | general hospital | | | Faculty physicians received a larger amount of medical samples and promotional material and were more prone to believe that gifts and lunches do not influence their prescribing behavior (42.2% vs. 23.6%; p=0.036) |
| 12 | Thomson et al., 1994 | New Zealand | Questionnaire survey of 67 general practitioners | Cross-sectional | Interactions with PSR | Most general practitioners (67%) had a negative attitude toward PSR interaction |
| 13 | Kamal et al., 2015 | Egypt | Interviews with 18 physicians | Cross-sectional | Interaction with PSR | Positive attitude towards PSR interaction |
| 14 | Hodges, 1995 | Canada | Survey in 105 residents of psychiatry | Cross-sectional | Interaction with PSR, drug samples, lunches | Positive attitude towards PSR interaction (56.5% of participants). The more money and promotional items a participant had received, the more likely he or she was to believe that discussions with representatives did not affect prescribing |

For peer review only

| | | | | | | |
|---|---|---|---|---|---|---|
| | | | | | | ($p < 0.05$) |
| 15 | Gibbons et al., 1998 | USA | Survey of 392 physicians in two tertiary-care medical centers | Cross-sectional | PSR interactions,gifts, samples, travel, lunches | Positive attitude towards PSR interactions, gifts, samples and lunches |
| 16 | Spingarn et al., 1996 | USA | 75 internal medicine physicians in university medical center | Retrospective cohort | PSR interaction (teaching) | Attendees inappropriately prescribed PSR speakers drug compared to non-attendees ($p=0.029$) |
| 17 | Zaki, 2014 | Saudi Arabia | Survey of 250 physicians | Randomized, cross-sectional survey | Conferences, drug samples | Favorable towards promotion |
| 18 | Orlowski et al., 1994 | USA | 10 physicians that were invited for a symposium and tracking the pharmacy inventory usage reports for these drugs before and after the symposia | Cohort | Conference travel | Significant increase in the prescribing pattern of drugs occurred following the symposia ($p<0.001$) |
| 19 | Scheffer et al., 2014 | Brazil | Survey of 300 physicians prescribing antiretroviral drugs | Cross-sectional | Interaction with representative, drug samples, journals | Frequency of interaction; the majority of (64%) of the physicians had multiple forms of interactions with PSR |
| 20 | Brett et al., 2003 | USA | Questionnaire of 93 physicians in a medical school | Cross-sectional | Interaction with PSR | Impact on attitudes; most physicians believed that most of PSR activities do not pose major ethical problems |
| 21 | Gupta et al., 2016 | India | Survey of 81 physicians in single hospital | Cross-sectional | Interaction with representative, drug samples, | Impact on prescribing; 61.7% of |

For peer review only

| | | | | | | |
|---|---|---|---|---|---|---|
| | | | | | journals | participants think that PSR has an impact on their prescribing (P = 0.0001) |
| 22 | Morgan et al., 2006 | USA | Survey of 397 obstetrician-gynaecologists | Cross-sectional | Drug samples, promotional material, lunch | Impact on prescribing, positive attitudes; most respondents thought it's proper to accept drug samples (92%), lunch (77%), an anatomical model (75%) or a well-paid consultantship (53%) from PSR |
| 23 | Alosaimi et al., 2014 | Saudi Arabia | Survey of 659 physicians | Cross-sectional | Interaction with PSR | Positive attitude towards PSR interaction |
| 24 | Chren et al., 1994 | USA | 40 case physicians and 80 control physicians | Case control | PSR interactions, honoraria, research | Increased prescription of company's drug after PSR interaction, honoraria and research (p<0.001, all) |
| 25 | Randall et al., 2005 | USA | Intervention group of physicians (n=18) that received education about PSR interaction and control group (n=14) | Controlled trial | Interaction with PSR | The majority of residents found the interactions and gifts useful. Compared to the comparison group, the intervention group significantly |

For peer review only

| | | | | | | |
|---|---|---|---|---|---|---|
| | | | | | | decreased the reported number of office supplies and noneducational gifts (p<0.05) |
| 26 | Caudil et al., 1996 | USA | Survey of 446 primary care physicians | Cross-sectional | Interaction with PSR | Significant positive correlation between physician cost of prescribing and perceived credibility, availability, applicability, and use of information provided by PSR ($p < 0.01$) |
| 27 | Andaleeb et al., 1995 | USA | 223 physicians in northwestern Pennsylvania | Cross-sectional | Interaction with PSR | Positive attitude towards PSR interaction |
| 28 | Reeder et al., 1993 | USA | 87 residents of emergency medicine | Cross-sectional | Interaction with PSR, gifts | Most participants believed that PSR interaction had no impact on their prescribing |
| 29 | Lichstein et al., 1992 | USA | 272 directors of internal medicine residency programs | Cross-sectional | Interaction with PSR | Most participants had a positive attitude towards PSR interactions |
| 30 | Brotzman et al., 1992 | USA | Directors of 386 family practice residency programs | Cross-sectional | Interaction with PSR | Majority of programs do not have guidelines for interaction with PSR |
| 31 | Alssageer et al., 2012 | Libya | Survey of 608 physicians in public | Cross-sectional | Interaction with PSR, drug | Positive attitude |

For peer review only

| | | | | | | |
|---|---|---|---|---|---|---|
| | | | and private practice settings | | samples, printed materials | towards PSR interactions |
| 32 | Lieb & Brandtonies, 2010 | Germany | Survey of 208 physicians (neurology, cardiology and general medicine) | Cross-sectional | Interaction with PSR, drug samples, printed materials, lunches | Frequency and impact on attitudes |
| 33 | Lieb & Scheurich, 2014 | Germany | Survey of 160 physicians in private and public practices | Cross-sectional | Interaction with representative, drug samples, printed materials, CME | High expenditure prescribing; avoidance of industry-sponsored CME is associated with more rational prescribing habits |
| 34 | Lieb & Koch, 2013 | Germany | Survey of 1038 medical students at 8 universities | Cross-sectional | Interaction with representative, drug samples, printed materials, lunches | Most participants have contact with the pharmaceutical company; 24.6% of the participants thought gifts would influence their future prescribing behavior, while 45.1% thought gifts would influence their classmates' future prescribing behavior ($p<0.001$) |
| 35 | Brown et al., 2015 | USA | 251 directors of family medicine residency programs | Cross-sectional | Interaction with PSR, gifts, lunches | Negative attitude towards PSR interactions |
| 37 | Rahmana et al., 2015 | Bangladesh | Survey of 83 village physicians | Cross-sectional | Interaction with PSR | Impact on their prescribing |
| 38 | Lee & Begley, 2016 | USA | Nationally representative survey of 4720 physicians | Cross-sectional | Gifts | Gifts were associated with lower perceived |

| | | | | | | |
|---|---|---|---|---|---|---|
| | | | | | | quality of patient care; an inverse relationship between the frequency of received gifts and the perceived quality of care was observed |
| 39 | Montastruc et al., 2014 | France | Survey among 631 medical residents | Cross-sectional | Interaction with representative | Most participants believed that PSR interaction had no impact on their prescribing; participants who had a more positive opinion were more frequently exposed to PSR (p<0.001) |
| 40 | Ketis & Kersnik, 2013 | Slovenia | 895 family physicians at the primary level of care | Cross-sectional | Interaction with PSR | Positive effect on knowledge; Participants value PSRs' selling and communication skills and trustworthiness highly |
| 41 | Hurley et al., 2014 | USA | 3500 dermatologists | Cross-sectional | Free drug samples | Impact on their prescribing; the provision of samples with a prescription by dermatologists has been increasing over time, and this |

For peer review only

| | | | | | | |
|---|---|---|---|---|---|---|
| | | | | | | increase is correlated (r = 0.92) with the use of the branded generic drugs promoted by these sample |
| 42 | Makowska, 2014 | Poland | Survey of 382 physicians | Cross-sectional | Gifts | Positive attitude towards PSR interactions |
| 43 | Siddiqui et al., 2014 | Pakistan | Questionnaires of 352 medical students | Cross-sectional | Interaction with representative | Positive attitude towards PSR interaction |
| 55 | Workneh BD et al., 2016 | Ethiopia | Survey of 90 physicians from public and private health facilities | Cross-sectional | Interaction with representative, gifts | Positive attitude towards industry, impact on prescribing behavior; Nearly half of the physicians reported that their prescribing decisions were influenced by PSR |
| 57 | Khan N et al., 2016 | Pakistan | Questionnaires in 472 physicians | Cross-sectional | Interaction with representative, gifts | Positive attitude towards PSR interaction |
| 58 | Saito S et al. 2010 | Japan | 1417 physicians working in internal medicine, general surgery, orthopedic surgery, pediatrics, obstetrics-gynecology, psychiatry, and ophthalmology | National Survey | Interaction with industry, receipt of gifts, funds, CME, samples | Positive attitude towards PSR and gifts, value information from PSR, interactions higher with physicians who prefer to prescribe brand names |
| 59 | Ziegler MG et al. 1995 | USA | 27 physicians working in public and private hospitals | Survey | Accuracy of information provided by | Incorrect information often |

For peer review only

| | | | | | | |
|---|---|---|---|---|---|---|
| | | | | | PSRs about drugs | provided by speakers goes unnoticed by physicians |
| 60 | Lurie N et al., 1990 | USA | 240 internal medicine faculty physicians in academic medical centers | Survey | Effect of interaction with PSR, free meals, honoraria and research support | Impact on prescribing behavior and formulary change requests |
| 62 | DeJong C et al., 2016 | USA | 279.669 physicians who wrote Medicare prescriptions in any of 4 drug classes: statins, cardioselective β-blockers, angiotensin-converting enzyme inhibitors and angiotensin-receptor blockers (ACE inhibitors and ARBs), and selective serotonin and serotonin-norepinephrine reuptake inhibitors (SSRIs and SNRIs) Physicians | Cross-sectional | Industry sponsored meals | Receipt of industry-sponsored meals was associated with an increased rate of brand name prescription. |
| 63 | Yeh JS et al., 2016 | USA | All licensed Massachusetts physicians who wrote prescriptions for statins paid for under the Medicare drug benefit in 2011 (n=2444) | Cross-sectional | Effect of industry payment on prescription of branded drugs for cholesterol control | Payment for meals and educational programs increased prescription of brand name statins. |
| 65 | Bowman MA et al., 1988 | USA | 121 physician attendees | Self report survey | Effect of CME on prescribing behavior | Sponsoring company's drugs were favored during prescription |
| 66 | Fischer MA et al., 2009 | USA | Multi-disciplinary focus groups with 61 physicians | Survey | Effect of industry marketing strategies on prescription and cognitive dissonance of physicians | Most participants reported no PSR impact on their prescribing, value to have ability to evaluate |

For peer review only

| | | | | | | |
|---|---|---|---|---|---|---|
| | | | | | | information of PSRs |
| 67 | Chimonas S et al., 2007 | USA | Six focus groups in 32 academic and community physicians | Survey | PSR interactions | Positive attitude towards PSR interaction |
| 72 | Yeh JS et al., 2014 | USA | 1610 US medical students | Cross-sectional | Interaction with representative, gifts, lunches | Policies separating students from representatives reduced number of interactions |
| 73 | Larkin I et al., 2014 | USA | Pediatricians, child & adolescent psychiatrists in five medical centers | Survey | Interaction with PSR | Anti detailing policies reduced the prescription of off-label antidepressants and anti psychotics for children |
| 74 | Esmaily HM et al., 2010 | Iran | 112 general physicians were randomized in two groups: 1) outcome-based educational intervention for rational prescribing and 2) concurrent CME program in the field of rational prescribing | Randomized trial | Effect of outcome and retinal prescribing | Rational prescribing improved in some of the important outcome-based indicators. No difference between two arms of the study |
| 76 | Parikh K et al., 2016 | USA | descriptive, cross-sectional analysis of Open Payments data and 9 638 825 payments to physicians and pediatricians from January 1 to December 31, 2014 | Cross-sectional | Comparison of PSR interactions between pediatricians and other specialists; among subspecialities of pediatrics. | Pediatricians get fewer gifts from PSR than internists. There is variation among sub specialities for extent of interaction. |
| 78 | Chressanthis GA et al. 2012 | USA | Clinical decisions of 72,114 physicians were statistically analyzed using prescription data | Survey | Effect of restricting PSRs on clinical practice and knowledge | Restricting PSRs affected information flow about drugs, both |

For peer review only

| | | | | | | |
|---|---|---|---|---|---|---|
| | | | | | | negative and positive. |

. We excluded 2000 records as they were not relevant (n = 1641), not original research (n=269), about medical students (n=4) and non-medical (e.g. ecological, econometric; n=86).

*Characteristics of included studies*

The identified studies were published between 1992 and August 2016. Most of the studies included were cross-sectional studies[1,9-13,19,21,22,31-55]. Only two studies were cohort studies[56, 57], three were randomized trials[58-60] and one study was a case-control study[61].

*Extent of interactions between physicians and the pharmaceutical industry*

We found that PSR interactions are a regular feature in the daily lives of physicians across the world [9-11,13,42,50]. Most of the attending physicians and residents have at least one interaction with industry representatives per month [10,21,22,36,42]. The frequency of interactions or gifts offered and accepted varies with private versus public hospital setting and the position of the physicians in the medical hierarchy [10,13,31,38,42,43,50,58,62]. Junior residents received twice as much free drug samples from PSR interactions than senior residents [10]. PSR interactions were significantly higher at the beginning of residency[13]. The majority of program directors of internal medicine residencies in the USA allowed PSRs to meet with residents during working hours and permitted PSR sponsorship of conferences[40]. Attending physicians and physician specialists had more PSR interactions and received higher numbers of medical samples and promotional material than residents[9,42]. Participants working in private practice alone or in both sectors were more likely to receive gifts than physicians working in the public sector [42,50]. Physicians in academic or hospital-based practice settings had less PSR interactions and significantly lower prescribing costs than physicians in nonacademic and nonhospital practices[38]. Most common gifts received were medical samples[9,21,22,31,36,37,42,63], promotional material[9,34,42] invitations for dinners[9], invitations for CMEs[22,34], scientific journals[34] and free lunches[21,37].

*Perspectives of physicians towards PSR interactions*

We found that physicians have a positive attitude towards PSRs[1, 13, 19, 20, 22, 31, 32, 40, 49, 58, 64] Physicians perceived PSRs as important sources of education and funding[10, 22, 32, 43, 45, 46]; while some studies reporting skeptical attitudes about the contribution of PSRs towards teaching and education[21, 36, 39, 40, 49]. Conference registration fees, informational luncheons, sponsorship of departmental journal clubs, anatomical models, and free drug samples were

For peer review only

considered as appropriate gifts[19, 39, 51, 58]. Most of the physicians considered pharmaceutical information provided by PSRs, industry sponsored conferences and CME events as important instruments for enhancing their scientific knowledge[22, 32, 45, 46]. Compared to senior residents, significantly more junior residents felt that pharmaceutical representatives have a valuable teaching role [10].

Most of the physicians considered themselves immune to the influence of gifts[1, 10, 32, 33, 35, 37, 43, 59]. We found that better scores on knowledge and attitudes were significantly associated with lesser number of interactions with representatives and their gifts[19]. Most studies found that physicians do not believe that PSR interactions impact their prescribing behavoir,[1, 9-13, 65, 66], while other studies found that there was some extent of influence[21, 22, 34, 36, 37, 39, 43]. In addition, physicians considered their colleagues more susceptible than themselves to PSR marketing strategies[1, 20, 21, 37, 43]. There was a strong correlation between the amount of gifts and the belief that PSR interactions did not influence their prescribing behavior [10].

*Gifts*

Most common gifts received were medical samples[9, 21, 22, 31, 36, 37, 42-44, 47], promotional material[9, 34, 42, 67] invitations for dinners[9] and scientific journals[34].

*Drug samples*

Most of the physicians who accepted drug samples had a positive attitude towards the pharmaceutical representatives[9, 21, 22, 31, 36, 37, 42, 43]. Accepting samples lead to higher branded drug prescription rather than generic prescribing [22,47].

*Pharmaceutical representative speakers*

Sponsored lectures/symposia of pharmaceutical companies influenced behavior of the attendees leading to the attendees prescribing more drugs from the sponsoring companies without sufficient evidence supporting superiority of those drugs[56,57]. The majority of attending physicians failed to identify inaccurate information about the company drug[18].

*Honoraria and Research Funding*

Physicians who received money to attend pharmaceutical symposia or to perform research requested formulary addition of the company's drug more often than other physicians, This association was independent of many confounding factors[61] (Table 2). Brief encounters with PSRs and receipt of honoraria or research support were predictors of faculty requested

For peer review only

change in hospital formulary[68].

*Conference travel*

Pharmaceutical company sponsored conference travels to touristic locations have quantifiable impact on the prescribing rational of attendees. A significant increase (three times) in the prescribing rate of two company drugs was observed after the physicians attended a company sponsored symposium with all their expenses covered. Despite this significant difference in the prescribing patterns, physicians insisted there was no impact on their prescribing behaviour.[57]

*Industry paid lunches*

Most physicians received invitations for dinners[9] and free lunches[10, 21, 35, 43] . Clerks, interns and junior residents attended more company sponsored lunches than senior residents [10]. Pharmaceutical companies also sponsored departmental lunches during journal clubs[39]. There was no significant association between attending industry paid lunches[37] and dinners[9] and formulary request for that company's drug (Table 2).

| # | Attitudes | Prescribing behavior | Knowledge | Formulary requests | Quality of Evidence (GRADE) |
|---|---|---|---|---|---|
| **Gifts** | Receiving higher number of gifts associated with belief that PRs have no impact on their prescribing behaviour [1,14,39] | - | - | - | **Moderate** |
| **Drug samples** | Positive attitude towards the drug industry and the representatives[11,21,34] | Higher prescription of the company drug[21, 41] | - | - | **High** |
| **Pharmaceutical representative speakers** | - | Irrational prescribing [16, 18, 34] | Inability to identify false claims[16] | Increased prescription of sponsor's drug[24] | **High** |
| **Honoraria and Research Funding** | Positive attitude towards sponsor's drug [60] | - | - | Increased prescription of sponsor's drug[24] | **Low** |

For peer review only

| | | | | | |
|---|---|---|---|---|---|
| **Conference travel** | - | Significant increase in prescribing of sponsor drug[18] | - | Increased prescription of sponsor's drug[24] | **Low** |
| **Industry paid lunches** | Positive attitude towards sponsor's drug [14, 34] | Significant increase in prescribing of sponsor drug[62] | - | Increased formulary request for company drug [11,21] | **High** |
| **CME sponsorship** | Positive attitude towards sponsor's drug [24, 65] | Avoidance of industry-sponsored CME associated with more rational prescribing habits [33] | | | **Moderate** |
| **Interaction with PRS** | Positive attitude towards PSR drugs [1,11,14,58] | Higher prescription of the company drug[24] | Positive correlation between the physicians' prescribing cost and the information provided by the drug representative during the interaction[26] | Increased prescription of sponsor's drug[24] | **High** |

However, there was a significant association between attending industry paid lunches and increased prescription of branded drugs [52,53, 69].

*CME sponsorship*

Physicians who attended company sponsored CME events had more positive attitudes towards and inclination to prescribe the branded drugs[28, 34, 43, 67, 70-72]. We found that physicians who refused CME sponsorship were seen to prescribe higher proportion of generics and lower expenditure medicines when compared to physicians who attended CMEs[22].

## Discussion

We report that there is widespread interaction between the pharmaceutical industry and physicians[9-11, 13, 42, 50]. Interactions are in the form of personal communications, free gifts such as drug samples, sponsored meals, sponsored conference travel, funding for research and CMEs and honoraria[9, 21, 22, 31, 36, 42]. The frequency of these interactions is comparable

For peer review only

between residents and physicians[10, 21, 22, 36, 42]. However, the amount and type of gifts vary with the position of the physician in medical hierarchy, specialization and location of practice[10, 13, 31, 38, 42, 43, 50, 58, 62]. In general, trainees (residents, interns) are treated with more drug samples, stationery items and free meals than senior physicians[10,13]. Senior physicians usually avail of sponsored conferences/ trips, research funding, honoraria and CME events. The extent of these interactions varies with academic versus non-academic institutions: non-academic hospitals record more interactions than others[31, 38, 42, 50, 55]. The majority of the physicians do not believe that they are affected by PSR interactions[1, 10, 32, 33, 35, 37, 43, 59] . However, a sizeable percentage in various surveys responded in the affirmative when asked whether they thought that their peers are vulnerable[1, 20, 21, 37, 43].

We observe that there is a positive correlation between acceptance of gifts and physicians' urge to reciprocate favorably towards the benefactor[12,22,47, 73, 74] Considering that physicians have a social contract with society at large to provide unbiased and altruistic service, this is an alarming observation. In 2005 a joint report by the WHO and Health Action International (HAI) reported on interventions to counter promotional activities.[75] The evidence presented in that report was not eligible for our systematic review, mostly because it related to interventions on students or residents. Nevertheless, the findings suggested that interventions such as industry self-regulation and guidelines for sales representatives are not effective, while education about drug promotion might influence physician attitudes[76-78].

*Policies and educational intervention*

The relationship of physicians with patients is of a fiduciary nature. Hence activities that might affect that relationship by altering physicians' clinical behavior are not acceptable. Physician-PSR interactions may put the trust of patients in physicians at risk. Interaction with PSRs begins early in the physicians' career. Trainees are exposed to PSR marketing and promotional techniques from the initial years of their medical education, which impact their prescribing behavior in future. Overall, trainees, i.e., residents and interns, are more vulnerable to PSR interactions than senior physicians[11, 41, 62] Physicians are susceptible to PSR interactions, which influences their clinical decision-making leading to greater prescriptions of branded drugs over low cost generic medicines and increasing healthcare cost[22, 47, 52, 53, 69] Therefore, there is need to institute and implement stringent policies curtailing physician-PSR relationships, as well as educational programs to increase awareness. Previous reports have indicated that implementing policies and conducting

For peer review only

educational programs are effective in increasing awareness of physician's attitudes towards PSR interactions[54, 59, 60, 76, 79-83]

*Strengths and Limitations of the study*

A major strength of this study is that is a large up-to-date systematic review of studies exploring the effects of physician and pharmaceutical industry representative interactions and residents in different settings (e.g. academic, primary care). Another strength of this study is the use of Cochrane and GRADE methodologies for conducting a review and assessing the quality of the studies. Moreover, we performed an extensive search in 3 databases and the grey literature. Some of the limitations of this review are related to the included studies, as some did not provide evidence for the significance of their findings or had varying study designs and outcomes, which made it impossible to conduct a meta-analysis. Also, the included studies were subject to risk of bias related to the lack of validity of outcome measurement, and inadequate handling of significant potential confounders.

*Future implications*

PSR interactions compromise the objectivity of the physicians. Educating physicians and increasing regulation of PSR interactions may lower the likelihood of prescribing new non-superior industry drugs and irrational prescription behavior. Further studies are required to evaluate the impact of PSR interactions on physicians over time and the benefits of various intervention based education programs on the clinical and ethical behavior of the physicians.

**References**


1. Steinman, M.A., M.G. Shlipak, and S.J. McPhee, *Of principles and pens: attitudes and practices of medicine housestaff toward pharmaceutical industry promotions.* The American Journal of Medicine, 2001. **110**(7): p. 551-557.
2. Wazana, A., *Physicians and the Pharmaceutical Industry.* JAMA, 2000. **283**(3): p. 373.
3. Wolfe, S.M., *Why do american drug companies spend more than $12 billion a year pushing drugs? Is it education or promotion?* Journal of General Internal Medicine, 1996. **11**(10): p. 637-639.
4. Wall, L.L. and D. Brown, *The High Cost of Free Lunch.* Obstetrics & Gynecology, 2007. **110**(1): p. 169-173.
5. Chressanthis, G.A., et al., *Determinants of pharmaceutical sales representative access limits to physicians.* Journal of Medical Marketing, 2014. **14**(4): p. 220-243.


For peer review only

6. International, K., *Rising costs of litigation in pharmaceutical industry.* Issues Monitor-Pharmaceuticals, 2011. **9**(14): p. 1-3.
7. MedAdNews, *Table insert on the top 50 pharma companies. Data provided by Cegedim Strategic Data.* 2010. **September**.
8. Mack J, *Pharma promotional spending in 2013.* Pharma marketing news, 2014. **13**(5).
9. De Ferrari, A., et al., *Attitudes and Relationship between Physicians and the Pharmaceutical Industry in a Public General Hospital in Lima, Peru.* PLoS ONE, 2014. **9**(6): p. e100114.
10. Hodges B. *Interactions with the pharmaceutical industry: experiences and attitudes of psychiatry residents, interns and clerks.* CMAJ: Canadian Medial Association Journal, 1995. **153**(5): p. 553.
11. Riese, F., et al., *Pharmaceutical industry interactions of psychiatric trainees from 20 European countries.* European Psychiatry, 2015. **30**(2): p. 284-290.
12. Lee, D. and C.E. Begley, *Physician report of industry gifts and quality of care.* Health Care Management Review, 2016. **41**(3): p. 275-283.
13. Montastruc, F., et al., *Interactions between Medical Residents and Drug Companies: A National Survey after the Mediator® Affair.* PLoS ONE, 2014. **9**(10): p. e104828.
14. Fugh-Berman, A. and S. Ahari, *Following the Script: How Drug Reps Make Friends and Influence Doctors.* PLoS Medicine, 2007. **4**(4): p. e150.
15. Kesselheim, A.S., M.M. Mello, and D.M. Studdert, *Strategies and Practices in Off-Label Marketing of Pharmaceuticals: A Retrospective Analysis of Whistleblower Complaints.* PLoS Medicine, 2011. **8**(4): p. e1000431.
16. Mulinari, S., *Unhealthy marketing of pharmaceutical products: An international public health concern.* Journal of Public Health Policy, 2016. **37**(2): p. 149-159.
17. Othman, N., et al., *Medicines information provided by pharmaceutical representatives: a comparative study in Australia and Malaysia.* BMC Public Health, 2010. **10**(1).
18. Ziegler, M.G., *The Accuracy of Drug Information From Pharmaceutical Sales Representatives.* JAMA: The Journal of the American Medical Association, 1995. **273**(16): p. 1296.
19. Alosaimi FD, A.K.A., Qadi M, Albahlal A, Alabdulkarim Y, Alabduljabbar M, Alqahtani F., *Physicians' attitudes towards interaction with the pharmaceutical industry/Attitudes des médecins par rapport á l'interaction avec l'industrie pharmaceutique.* Eastern Mediterranean Health Journal, 2014. **20**(12): p. 812.
20. Andaleeb, S.S. and R.F. Tallman, *Physician attitudes toward pharmaceutical sales representatives.* Health Care Management Review, 1995. **20**(3): p. 68-76.
21. Lieb K, B.S., *A survey of German physicians in private practice about contacts with pharmaceutical sales representatives.* Dtsch Arztebl Int., 2012. **107**(22): p. 392-8.
22. Lieb, K. and A. Scheurich, *Contact between Doctors and the Pharmaceutical Industry, Their Perceptions, and the Effects on Prescribing Habits.* PLoS ONE, 2014. **9**(10): p. e110130.
23. Francer, J., et al., *Ethical pharmaceutical promotion and communications worldwide: codes and regulations.* Philosophy, Ethics, and Humanities in Medicine, 2014. **9**(1): p. 7.
24. Canada., C.o.f.p.o.C.R.c.o.p.a.s.o., *Guide tot enhancing referrals and consultations between physicians.* College of Family Physicians of Canada., 2009.
25. Grande, D., *Limiting the Influence of Pharmaceutical Industry Gifts on Physicians: Self-Regulation or Government Intervention?* Journal of General Internal Medicine, 2009. **25**(1): p. 79-83.

For peer review only

26. Brennan, T.A., et al., *Health Industry Practices That Create Conflicts of Interest.* JAMA, 2006. **295**(4): p. 429.
27. Spurling, G.K., et al., *Information from Pharmaceutical Companies and the Quality, Quantity, and Cost of Physicians' Prescribing: A Systematic Review.* PLoS Medicine, 2010. **7**(10): p. e1000352.
28. J., Lexchin., *Interactions between physicians and the pharmaceutical industry: what does the literature say? .* CMAJ: Canadian Medial Association Journal, 1993. **14**(10): p. 1401-7.
29. Wazana, A., et al., *Using the Literature in Developing McGill's Guidelines for Interactions between Residents and the Pharmaceutical Industry.* Academic Medicine, 2004. **79**(11): p. 1033-1040.
30. Guyatt, G., et al., *GRADE guidelines: 1. Introduction—GRADE evidence profiles and summary of findings tables.* Journal of Clinical Epidemiology, 2011. **64**(4): p. 383-394.
31. Thomson AN, C.B., Barham PM., *Attitudes of general practitioners in New Zealand to pharmaceutical representatives.* Br J Gen Pract., 1994. **44**(382): p. 220-3.
32. Kamal, S., et al., *Perceptions and Attitudes of Egyptian Health Professionals and Policy-Makers towards Pharmaceutical Sales Representatives and Other Promotional Activities.* PLOS ONE, 2015. **10**(10): p. e0140457.
33. Gibbons, R.V., et al., *A comparison of physicians' and patients' attitudes toward pharmaceutical industry gifts.* Journal of General Internal Medicine, 1998. **13**(3): p. 151-154.
34. Scheffer, M.C., *Interaction between pharmaceutical companies and physicians who prescribe antiretroviral drugs for treating AIDS.* Sao Paulo Medical Journal, 2014. **132**(1): p. 55-60.
35. Brett, A.S., W. Burr, and J. Moloo, *Are Gifts From Pharmaceutical Companies Ethically Problematic?* Archives of Internal Medicine, 2003. **163**(18): p. 2213.
36. Gupta, S., R. Nayak, and R. Sivaranjani, *A study on the interactions of doctors with medical representatives of pharmaceutical companies in a Tertiary Care Teaching Hospital of South India.* Journal of Pharmacy and Bioallied Sciences, 2016. **8**(1): p. 47.
37. Morgan, M.A., *Interactions of doctors with the pharmaceutical industry.* Journal of Medical Ethics, 2006. **32**(10): p. 559-563.
38. Caudill, T.S., *Physicians, pharmaceutical sales representatives, and the cost of prescribing.* Archives of Family Medicine, 1996. **5**(4): p. 201-206.
39. Reeder, M., J. Dougherty, and L.J. White, *Pharmaceutical representatives and emergency medicine residents: A national survey.* Annals of Emergency Medicine, 1993. **22**(10): p. 1593-1596.
40. Lichstein PR, T.R., O'Brien K., *Impact of pharmaceutical company representatives on internal medicine residency programs: a survey of residency program directors.* Archives of Internal Medicine, 1992. **152**(5): p. 1009-13.
41. Brotzman GL, M.D., *Policies regulating the activities of pharmaceutical representatives in residency programs.* Journal of Family Practice, 1992. **34**(1): p. 54-8.
42. Alssageer, M. and S. Kowalski, *A survey of pharmaceutical company representative interactions with doctors in Libya.* Libyan Journal of Medicine, 2012. **7**(1): p. 18556.
43. Lieb K, K.C., *Medical students' attitudes to and contact with the pharmaceutical industry: a survey at eight German University hopitals.* Dtsch. Arztebl. Int., 2013. **110**(35): p. 584-90.


For peer review only

44. Brown SR, E.D., Berman AF., *Pharmaceutical industry interactions in family medicine residencies decreased between 2008 and 2013: a CERA study.* Fam. Med., 2015. **47**(4): p. 279-82.
45. Rahmana MH, A.S., Tuddenhama S, Petoa H, Iqbalb M, Bhuiyab A, Peters DH., *What do they do? Interactions between village doctors and medical representatives in Chakaria, Banladesh.* Int. Health., 2015. **7**(1): p. 266-71.
46. KlemencKetis, Z. and J. Kersnik, *Which Pharmaceutical Sales Representatives' Features do Slovenian Family Physicians Value?* Acta Informatica Medica, 2013. **21**(4): p. 257.
47. Hurley, M.P., R.S. Stafford, and A.T. Lane, *Characterizing the Relationship Between Free Drug Samples and Prescription Patterns for Acne Vulgaris and Rosacea.* JAMA Dermatology, 2014. **150**(5): p. 487.
48. Makowska, M., *Interactions between Doctors and Pharmaceutical Sales Representatives in a Former Communist Country.* Cambridge Quarterly of Healthcare Ethics, 2014. **23**(03): p. 349-355.
49. Siddiqui, U.T., et al., *Attitudes of medical students towards incentives offered by pharmaceutical companies- perspective from a developing nation- a cross sectional study.* BMC Medical Ethics, 2014. **15**(1).
50. Workneh, B.D., et al., *Influence of Medical Representatives on Prescribing Practices in Mekelle, Northern Ethiopia.* PLOS ONE, 2016. **11**(6): p. e0156795.
51. Khan, N., et al., *Perceptions and Attitudes of Medical Sales Representatives (MSRs) and Prescribers Regarding Pharmaceutical Sales Promotion and Prescribing Practices in Pakistan.* Journal of Young Pharmacists, 2016. **8**(3): p. 244-250.
52. DeJong, C., et al., *Pharmaceutical Industry–Sponsored Meals and Physician Prescribing Patterns for Medicare Beneficiaries.* JAMA Internal Medicine, 2016. **176**(8): p. 1114.
53. Yeh, J.S., et al., *Association of Industry Payments to Physicians With the Prescribing of Brand-name Statins in Massachusetts.* JAMA Internal Medicine, 2016. **176**(6): p. 763.
54. Yeh, J.S., et al., *Association of Medical Students' Reports of Interactions with the Pharmaceutical and Medical Device Industries and Medical School Policies and Characteristics: A Cross-Sectional Study.* PLoS Medicine, 2014. **11**(10): p. e1001743.
55. Parikh, K., W. Fleischman, and S. Agrawal, *Industry Relationships With Pediatricians: Findings From the Open Payments Sunshine Act.* PEDIATRICS, 2016. **137**(6): p. e20154440-e20154440.
56. Spingarn, R.W., J.A. Berlin, and B.L. Strom, *When pharmaceutical manufacturers' employees present grand rounds, what do residents remember?* Academic Medicine, 1996. **71**(1): p. 86-8.
57. Orlowski, J.P. and L. Wateska, *The Effects of Pharmaceutical Firm Enticements on Physician Prescribing Patterns: there's no such thing as a free lunch.* Chest, 1992. **102**(1): p. 270-273.
58. Zaki, N.M., *Pharmacists' and physicians' perception and exposure to drug promotion: A Saudi study.* Saudi Pharmaceutical Journal, 2014. **22**(6): p. 528-536.
59. Randall, M., Rosenbaum JR, Rohrbaugh RM, Rosenheck RA., *Attitudes and Behaviors of Psychiatry Residents Toward Pharmaceutical Representatives Before and After an Educational Intervention.* Academic Psychiatry, 2005. **29**(1): p. 33-39.
60. Esmaily, H.M., et al., *Can rational prescribing be improved by an outcome-based educational approach? A randomized trial completed in Iran.* Journal of Continuing Education in the Health Professions, 2010. **30**(1): p. 11-18.

For peer review only

61. Chren MM, L.C., *Physicians' Behavior and Their Interactions With Drug Companies: a controlled study of physicians who requested additions to a hospital drug formulary.* JAMA, 1994. **271**(9): p. 684.
62. Zipkin, D.A. and M.A. Steinman, *Interactions between pharmaceutical representatives and doctors in training.* Journal of General Internal Medicine, 2005. **20**(8): p. 777-786.
63. McMurray, R.J., *Gifts to Physicians From Industry.* JAMA: The Journal of the American Medical Association, 1991. **265**(4): p. 501.
64. Lotfi, T., et al., *Knowledge, beliefs and attitudes of physicians in low and middle-income countries regarding interacting with pharmaceutical companies: a systematic review.* BMC Health Services Research, 2016. **16**(1).
65. Fischer, M.A., et al., *Prescribers and Pharmaceutical Representatives: Why Are We Still Meeting?* Journal of General Internal Medicine, 2009. **24**(7): p. 795-801.
66. Chimonas, S., T.A. Brennan, and D.J. Rothman, *Physicians and Drug Representatives: Exploring the Dynamics of the Relationship.* Journal of General Internal Medicine, 2007. **22**(2): p. 184-190.
67. Saito, S., K. Mukohara, and S. Bito, *Japanese Practicing Physicians' Relationships with Pharmaceutical Representatives: A National Survey.* PLoS ONE, 2010. **5**(8): p. e12193.
68. Lurie, N., et al., *Pharmaceutical representatives in academic medical centers.* Journal of General Internal Medicine, 1990. **5**(3): p. 240-243.
69. Steinbrook, R., *Industry Payments to Physicians and Prescribing of Brand-name Drugs.* JAMA Internal Medicine, 2016. **176**(8): p. 1123.
70. Bowman, M.A. and D.L. Pearle, *Changes in drug prescribing patterns related to commercial company funding of continuing medical education.* Journal of Continuing Education in the Health Professions, 1988. **8**(1): p. 13-20.
71. Grundy Q, B.L., Malone R 2013, *Interactions between non-physician clinicians and industry: a systematic review.* PLoS Med. , 2013. **11**(Nov 26).
72. Dixon, D., et al., *Controlling Quality in CME/CPD by Measuring and Illuminating Bias.* Journal of Continuing Education in the Health Professions, 2011. **31**(2): p. 109-116.
73. Silverman, E., *Everything you need to know about the Sunshine Act.* BMJ, 2013. **347**(jul26 1): p. f4704-f4704.
74. Shalowitz, D.I., M.A. Spillman, and M.A. Morgan, *Interactions with industry under the Sunshine Act: an example from gynecologic oncology.* American Journal of Obstetrics and Gynecology, 2016. **214**(6): p. 703-707.
75. Norris P, H.A., Lexchin J, et al., *Drug promotion—what we know, what we have yet to learn—reviews of materials in the WHO/HAI database on drug promotion.* EDM Research Series, 2005(No. 032.).
76. Carroll, A.E., et al., *To What Extent Do Educational Interventions Impact Medical Trainees' Attitudes and Behaviors Regarding Industry-Trainee and Industry-Physician Relationships?* PEDIATRICS, 2007. **120**(6): p. e1528-e1535.
77. Birkhahn, R.H., et al., *A Review of the Federal Guidelines That Inform and Influence Relationships Between Physicians and Industry.* Academic Emergency Medicine, 2009. **16**(8): p. 776-781.
78. Chressanthis, G.A., et al., *Can Access Limits on Sales Representatives to Physicians Affect Clinical Prescription Decisions? A Study of Recent Events With Diabetes and Lipid Drugs.* The Journal of Clinical Hypertension, 2012. **14**(7): p. 435-446.

For peer review only

79. Sergeant MD, H.P., Godwin M, Walker DM, McHenry P., *Interactions with the pharmaceutical industry: a survey of family medicine residents in Ontario.* CMAJ: Canadian Medial Association Journal, 1996. **155**(9): p. 1243.
80. T Montague, B., A.H. Fortin Vi, and J. Rosenbaum, *A systematic review of curricula on relationships between residents and the pharmaceutical industry.* Medical Education, 2008. **42**(3): p. 301-308.
81. Larkin, I., et al., *Restrictions On Pharmaceutical Detailing Reduced Off-Label Prescribing Of Antidepressants And Antipsychotics In Children.* Health Affairs, 2014. **33**(6): p. 1014-1023.
82. R., P., *Guidelines for faculty involvement in commercially supported continuing medical-education.* Academic Medicine, 1992.
83. Greenland, P., *Time for the Medical Profession to Act.* Archives of Internal Medicine, 2009. **169**(9): p. 829.

**Legends**

**Figure 1:** PRISMA flow diagram showing search strategy and included studies

**Table 1:** Characteristics of included studies

**Table 2:** Impact of physician-pharmaceutical industry interaction on physician

**The Corresponding Author has the right to grant on behalf of all authors and does grant on behalf of all authors, a worldwide licence (http://www.bmj.com/sites/default/files/BMJ%20Author%20Licence%20March%2020 13.doc) to the Publishers and its licensees in perpetuity, in all forms, formats and media (whether known now or created in the future), to i) publish, reproduce, distribute, display and store the Contribution, ii) translate the Contribution into other languages, create adaptations, reprints, include within collections and create summaries, extracts and/or, abstracts of the Contribution and convert or allow conversion into any format including without limitation audio, iii) create any other derivative work(s) based in whole or part on the on the Contribution, iv) to exploit all subsidiary rights to exploit all subsidiary rights that currently exist or as may exist in the future in the Contribution, v) the inclusion of electronic links from the Contribution to third party material where-ever it may be located; and, vi) licence any third party to do any or all of the above. All research articles will be made available on an Open Access basis (with authors being asked to pay an open access fee—see http://www.bmj.com/about-bmj/resources-**

For peer review only

**authors/forms-policies-and-checklists/copyright-open-access-and-permission-reuse). The terms of such Open Access shall be governed by a Creative Commons licence—details as to which Creative Commons licence will apply to the research article are set out in our worldwide licence referred to above.**

For peer review only

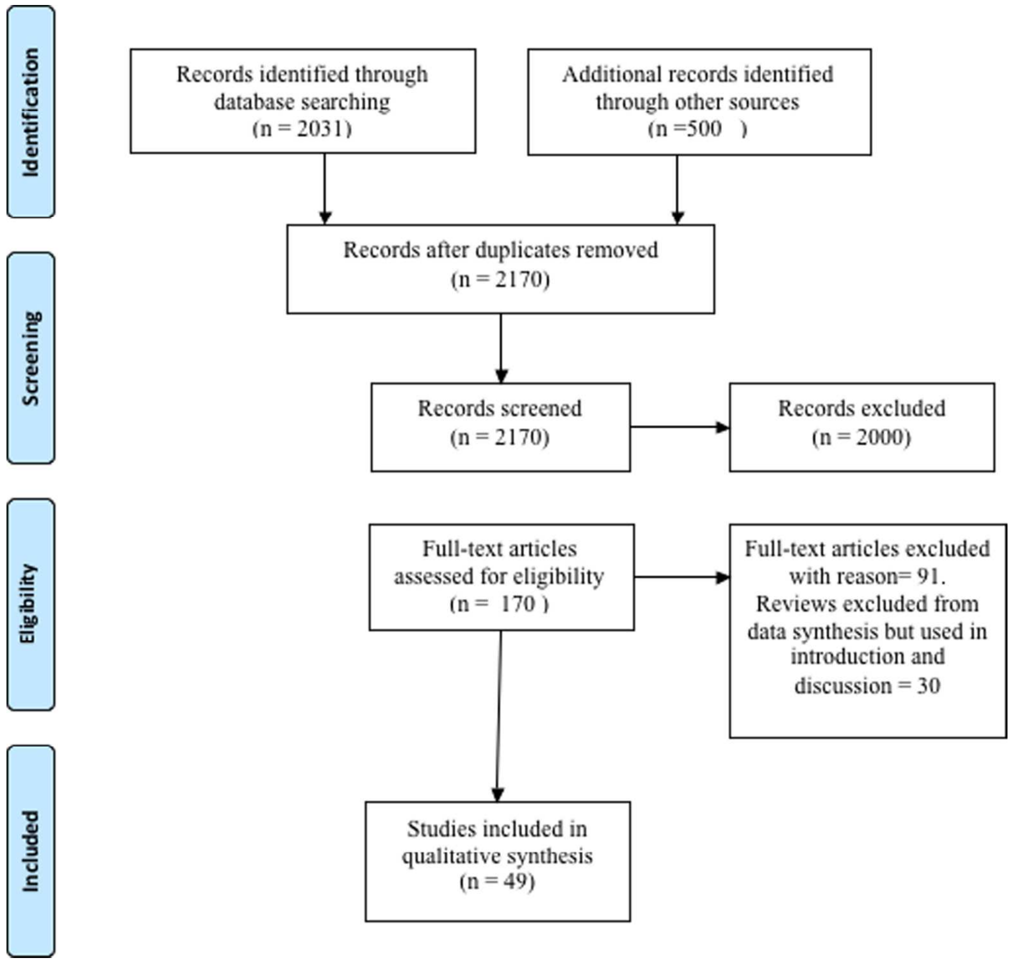


Flow diagram of study selection.

only

Search strategy for PubMed search engine of Medline

In an attempt to find all related literature on the topic, studies related to physician-pharmaceutical representative interactions that affect the prescribing behavior of the physicians were identified through computerized searches using, but not limited to, the following subject headings and text words in PubMed from 1992 to 2016.

1. Physician interactions with pharmaceutical industry
2. Physician attitude towards pharmaceutical representatives
3. Behavior of physicians towards pharmaceutical representatives
4. Gifts AND physician AND pharmaceutical representatives
5. Honoraria AND physician AND pharmaceutical representatives
6. Continuing medical education AND physician AND pharmaceutical representatives
7. Research funding AND physician AND pharmaceutical representatives
8. Conference travel AND physician AND pharmaceutical representatives
9. Industry sponsored meals AND physician behavior

For peer review only

# PRISMA 2009 Checklist

| Section/topic | # | Checklist item | Reported on page # |
|---|---|---|---|
| **TITLE** | | | |
| Title | 1 | Identify the report as a systematic review, meta-analysis, or both. | 1 |
| **ABSTRACT** | | | |
| Structured summary | 2 | Provide a structured summary including, as applicable: background; objectives; data sources; study eligibility criteria, participants, and interventions; study appraisal and synthesis methods; results; limitations; conclusions and implications of key findings; systematic review registration number. | 2 |
| **INTRODUCTION** | | | |
| Rationale | 3 | Describe the rationale for the review in the context of what is already known. | 3 |
| Objectives | 4 | Provide an explicit statement of questions being addressed with reference to participants, interventions, comparisons, outcomes, and study design (PICOS). | 3 |
| **METHODS** | | | |
| Protocol and registration | 5 | Indicate if a review protocol exists, if and where it can be accessed (e.g., Web address), and, if available, provide registration information including registration number. | 4 |
| Eligibility criteria | 6 | Specify study characteristics (e.g., PICOS, length of follow-up) and report characteristics (e.g., years considered, language, publication status) used as criteria for eligibility, giving rationale. | 3 |
| Information sources | 7 | Describe all information sources (e.g., databases with dates of coverage, contact with study authors to identify additional studies) in the search and date last searched. | 4 |
| Search | 8 | Present full electronic search strategy for at least one database, including any limits used, such that it could be repeated. | 4 |
| Study selection | 9 | State the process for selecting studies (i.e., screening, eligibility, included in systematic review, and, if applicable, included in the meta-analysis). | 3/4 |
| Data collection process | 10 | Describe method of data extraction from reports (e.g., piloted forms, independently, in duplicate) and any processes for obtaining and confirming data from investigators. | 4 |
| Data items | 11 | List and define all variables for which data were sought (e.g., PICOS, funding sources) and any assumptions and simplifications made. | 4 |
| Risk of bias in individual studies | 12 | Describe methods used for assessing risk of bias of individual studies (including specification of whether this was done at the study or outcome level), and how this information is to be used in any data synthesis. | 3 |
| Summary measures | 13 | State the principal summary measures (e.g., risk ratio, difference in means). | 3 |
| Synthesis of results | 14 | Describe the methods of handling data and combining results of studies, if done, including measures of consistency (e.g., $I^2$) for each meta-analysis. | 3 |

For peer review only - http://bmjopen.bmj.com/site/about/guidelines.xhtml

# PRISMA 2009 Checklist

| Section/topic | # | Checklist item | Reported on page # |
|---|---|---|---|
| Risk of bias across studies | 15 | Specify any assessment of risk of bias that may affect the cumulative evidence (e.g., publication bias, selective reporting within studies). | 3 |
| Additional analyses | 16 | Describe methods of additional analyses (e.g., sensitivity or subgroup analyses, meta-regression), if done, indicating which were pre-specified. | 3 |
| **RESULTS** | | | |
| Study selection | 17 | Give numbers of studies screened, assessed for eligibility, and included in the review, with reasons for exclusions at each stage, ideally with a flow diagram. | 5 |
| Study characteristics | 18 | For each study, present characteristics for which data were extracted (e.g., study size, PICOS, follow-up period) and provide the citations. | 5-8 |
| Risk of bias within studies | 19 | Present data on risk of bias of each study and, if available, any outcome level assessment (see item 12). | 5-8 |
| Results of individual studies | 20 | For all outcomes considered (benefits or harms), present, for each study: (a) simple summary data for each intervention group (b) effect estimates and confidence intervals, ideally with a forest plot. | 5-8 |
| Synthesis of results | 21 | Present results of each meta-analysis done, including confidence intervals and measures of consistency. | NA |
| Risk of bias across studies | 22 | Present results of any assessment of risk of bias across studies (see Item 15). | 5-8 |
| Additional analysis | 23 | Give results of additional analyses, if done (e.g., sensitivity or subgroup analyses, meta-regression [see Item 16]). | NA |
| **DISCUSSION** | | | |
| Summary of evidence | 24 | Summarize the main findings including the strength of evidence for each main outcome; consider their relevance to key groups (e.g., healthcare providers, users, and policy makers). | 8-9 |
| Limitations | 25 | Discuss limitations at study and outcome level (e.g., risk of bias), and at review-level (e.g., incomplete retrieval of identified research, reporting bias). | 9 |
| Conclusions | 26 | Provide a general interpretation of the results in the context of other evidence, and implications for future research. | 9 |
| **FUNDING** | | | |
| Funding | 27 | Describe sources of funding for the systematic review and other support (e.g., supply of data); role of funders for the systematic review. | 1 |

*From:* Moher D, Liberati A, Tetzlaff J, Altman DG, The PRISMA Group (2009). Preferred Reporting Items for Systematic Reviews and Meta-Analyses: The PRISMA Statement. PLoS Med 6(6): e1000097. doi:10.1371/journal.pmed1000097

For more information, visit: www.prisma-statement.org.



For peer review only - http://bmjopen.bmj.com/site/about/guidelines.xhtml

BMJ Open

# Association between pharmaceutical industry and its sales representatives' interactions on physicians' attitudes and prescribing habits: A systematic review

| | |
|---|---|
| Journal: | *BMJ Open* |
| Manuscript ID | bmjopen-2017-016408.R3 |
| Article Type: | Research |
| Date Submitted by the Author: | 10-Jul-2017 |
| Complete List of Authors: | Fickweiler, Freek; Crowd for Cure<br>Fickweiler, Ward; Crowd for Cure,<br>Urbach, Ewout; Crowd for Cure |
| <b>Primary Subject Heading</b>: | Health policy |
| Secondary Subject Heading: | Patient-centred medicine |
| Keywords: | Change management < HEALTH SERVICES ADMINISTRATION & MANAGEMENT, Health policy < HEALTH SERVICES ADMINISTRATION & MANAGEMENT, Protocols & guidelines < HEALTH SERVICES ADMINISTRATION & MANAGEMENT |

SCHOLARONE™
Manuscripts

# Association between pharmaceutical industry and its sales representatives' interactions on physicians' attitudes and prescribing habits: A systematic review

**Fickweiler F[1], Fickweiler W[1], Urbach E[1],**

**1: Crowd for Cure, Jacob van Ruysdaelstraat 34, 9718 SG Groningen, the Netherlands**

**Corresponding author's email address: freek@crowdforcure.com**

**All authors have completed the ICMJE uniform disclosure form at http://www.icmje.org/coi_disclosure.pdf and declare: no support from any organisation for the submitted work; no financial relationships with any organisations that might have an interest in the submitted work in the previous three years, no other relationships or activities that could appear to have influenced the submitted work**

**Contributor Statement: All authors have contributed equally and have substantial contributions to the conception or design of the work; Author Freek Fickweiler for the acquisition, analysis, and interpretation of data for the work; Author Freek Fickweiler for drafting the work and Authors Ewout Urbach and Ward Fickweiler for revising it critically for important intellectual content; and all authors (Freek Fickweiler, Ward Fickweiler and Ewout Urbach) contributed to final approval of the version to be published and agreed to be accountable for all aspects of the work in ensuring that questions related to the accuracy or integrity of any part of the work are appropriately investigated and resolved.**

**Competing interest: no financial relationships with any organisations that might have an interest in the submitted work in the previous three years, no other relationships or activities that could appear to have influenced the submitted work**

**Funding: all authors declare no support from any organisation for the submitted work.**

**Data sharing statement: any data relevant to a published article will be made available alongside the article when published.**

For peer review only

## Abstract

**Objectives**

The objective of this review is to explore the frequency of physician and pharmaceutical industry and its sales representatives interactions and their impact on physicians' attitude, knowledge and behavior.

**Data Sources**

Pubmed, Embase, Cochrane library and Google scholar electronic databases were searched from 1992 to August 2016 using free text words and medical subject headings relevant to the topic.

**Study Selection**

Studies included cross sectional studies, cohort studies, randomized trials and survey designs. Studies with narrative reviews, case reports, opinion polls, and letters to the editor,were excluded from data synthesis.

**Data Extraction**

Two reviewers independently extracted the data. Data on study design, study year, country, participant characteristics, setting, and number of participants were collected.

**Data Synthesis**

Pharmaceutical industry and pharmaceutical sales representative (PSR) interactions influence physicians' attitudes, their prescribing behavior and increases the number of formulary addition requests for the company's drug.

**Conclusion**

Physician-pharmaceutical industry and its sales representative's interactions and acceptance of gifts from the company's pharmaceutical sales representatives have been found to affect physicians' prescribing behavior and are likely to contribute to irrational prescribing of the company's drug. Therefore, intervention in the form of policy implementation and education about the implications of these interactions is needed.

**Strengths and limitations of the study**

- Large up-to-date systematic review of studies exploring the impact of pharmaceutical industry representative interactions on physicians

For peer review only

- This systematic review used the recommendations outlined in the Cochrane Handbook for conducting systematic reviews and the GRADE methodology to assess the quality of the evidence by outcome.

- Pubmed, Embase, Cochrane library and Google scholar electronic databases were searched from 1992, as well as grey literature.

- Most studies identified were observational and of varying methodological design

- Some studies did not provide evidence for the significance of their findings

**Keywords:** pharmaceutical sales representative; physicians, drug industry; brand prescriptions; conflict of interest ;physicians-industry interactions; pharmaceutical industry; attitude of health personnel; gifts to physicians; medical education; irrational prescriptions

For peer review only

## Introduction

The relationship between physicians and the pharmaceutical industry has evoked heated debate for many decades[1]. In 2012, the pharmaceutical industry spent $89.5 billion on physician-pharmaceutical sales representative (PSR) interactions which accounted for 60% of the global sales and marketing spending[2-8]. Previous reports have demonstrated that PSRs may influence prescribing behavior[,9-16]. However, the evidence determining whether or not pharmaceutical industry and PSRs interactions influence physicians is divided and contradictory. Studies have indicated that physicians may be unable to distinguish between promotional information and scientific evidence[17-18]. Physicians on the other hand believe their colleagues are more susceptible to pharmaceutical industry marketing strategies than themselves [19-22]. The majority of the physicians do not believe that they are affected by pharmaceutical industry and PSR interactions. Most medical and governmental institutions have installed guidelines and self-regulatory and legislative checks to regulate the relationship between physicians and the pharmaceutical industry and its representatives t[5,15,16, 23-26]. However, while administrative proposals for deregulatory reforms that would remove some governmental authority over the industry are increasing, scientific evidence rigorously examining the extent of interactions between physicians and pharmaceutical industry and it PSRs is needed. This review evaluates e critically and systemically the evidence on the impact of pharmaceutical industry and PSR interactions on physicians.

## Methodology

*Protocol*

We followed a detailed methodology that we described in our review protocol, which is available upon request to the corresponding author. Two independent reviewers assessed selected articles as per inclusion/exclusion criteria, shortlisted them for writing the review and cross-checked their decisions about inclusion/exclusion with each other. The review followed the Preferred Reporting Items for Systematic Reviews and Meta-Analyses (PRISMA) guidelines (Appendix 1).

*Eligibility criteria*

The eligibility criteria were:

For peer review

- Types of studies: cross sectional studies, cohort studies, randomized trials and survey designs comparing an intervention of interest on at least one facet of extent, impact on behavior and attitude.
- Types of participants: physicians, pharmaceutical representatives, physicians in training/residents. We did not consider medical students or other health professionals.
- Types of exposure: any type of interaction between physicians and the pharmaceutical industry where there is direct interaction with the physician, such as meeting with drug representatives, participating in pharmaceutical-sponsored CME event, receiving travel funding, free drug samples, industry-provided meals, gifts to the individual and active presentation of industry-related information to the physician
- Types of outcome: knowledge of physicians (e.g. accuracy of knowledge related to a specific medication), beliefs and/or attitudes of physicians regarding physician-industry interactions (e.g. perceived influence of information from the pharmaceutical company on their behavior), behavior of physicians (e.g. prescribing quantity/frequency)
- Type of control: no interaction.
- Exclusion criteria were: qualitative, ecological, econometric studies, editorials, letters to the editor, studies on medical students, small samples sizes, studies assessing non-targeted or indirect interactions (e.g. journal advertisement) and research funding.

We did not exclude studies based on risk of bias. We took risk of bias into account when grading the quality of evidence using GRADE approach.

*Search strategy*

The search strategy included Pubmed, Embase, Cochrane library and Google scholar electronic databases (January 1992 to August 2016). Databases were not searched before 1992, as these studies were already investigated in an earlier review[27]. The search combined terms for physicians and pharmaceutical, and included both free text words and medical subject heading relevant to the topic. We did not use a search filter. The supplementary information file provides the full details for one database. Additional search strategies included a search of the grey literature (theses and dissertations). Also, we reviewed the references lists of included and relevant papers.[27-29]

*Assessment of risk of bias in included studies*

Two reviewers assessed in duplicate and independently the riskof bias in each eligible study. Disagreements were resolved by discussion or adjudication by a third reviewer. We used the recommendations outlined in the Cochrane Handbook to assess the risk of bias in randomized studies. We graded each potential source of bias and rated the studies as high, low or unclear risk of bias.

*Data analysis and synthesis*

The information extracted from the selected studies included type of study, study design, type of pharmaceutical industry and PSR interaction and type of outcome. We did not conduct a meta-analysis due to the heterogeneity of study design, types of interventions, outcomes assessed, and outcome measures used. Instead, we summarized the data narratively. We assessed the quality of evidence by outcome using the GRADE methodology[30].

## Results

We independently screened the titles and abstracts of the 2170 identified records for potential eligibility. Out of 2170, the full text of 49 eligible citations which matched the inclusion criteria were retrieved and used for qualitative assessment during the writing of the review (Figure 1, Table 1)

| Study | Authors | Country | Timeframe | Participants, setting | Study design | Interaction | Outcomes |
|---|---|---|---|---|---|---|---|
| 1 | Steinman et al., 2001 | USA | Spring 1999 | Surveys about attitudes and behaviors toward industry gifts in 105 residents at a university-based internal medicine residency program | Cross-sectional | PSR interactions, gifts | Most participants (61%) hold positive attitudes toward gifts from industry and PSR interactions and believe they do not influence their own prescribing, but only 16% believed other physicians were |

For peer review only

| | | | | | | | |
|---|---|---|---|---|---|---|---|
| | | | | | | | similarly unaffected (P< .0001) |
| 11 | De Ferrari et al., 2014 | Peru | March 2013 | Questionnaire in 155 faculty and trainee physicians of five different clinical departments working in a public general hospital | Cross-sectional | PSR interactions, medical samples, promotional material, dinners | Positive attitude towards representatives (88.5% of participants). Faculty physicians received a larger amount of medical samples and promotional material and were more prone to believe that gifts and lunches do not influence their prescribing behavior (42.2% vs. 23.6%; p=0.036) |
| 12 | Thomson et al., 1994 | New Zealand | 1991 | Questionnaire survey of 67 general practitioners | Cross-sectional | Interactions with PSR | Most general practitioners (67%) had a negative attitude toward PSR interaction |
| 13 | Kamal et al., 2015 | Egypt | July and August 2013 | Interviews with 18 physicians | Cross-sectional | Interaction with PSR | Positive attitude towards PSR interaction |
| 14 | Hodges, 1995 | Canada | October 1993-February 1994 | Survey in 105 residents of psychiatry | Cross-sectional | Interaction with PSR, drug samples, lunches | Positive attitude towards PSR interaction (56.5% of participants). The more money and |

For peer review only

| | | | | | | | |
|---|---|---|---|---|---|---|---|
| | | | | | | | promotional items a participant had received, the more likely he or she was to believe that discussions with representatives did not affect prescribing ($p < 0.05$) |
| 15 | Gibbons et al., 1998 | USA | Not reported | Survey of 392 physicians in two tertiary-care medical centers | Cross-sectional | PSR interactions,gifts, samples, travel, lunches | Positive attitude towards PSR interactions, gifts, samples and lunches |
| 16 | Spingarn et al., 1996 | USA | February 1990 | 75 internal medicine physicians in university medical center | Retrospective cohort | PSR interaction (teaching) | Attendees inappropriately prescribed PSR speakers drug compared to non-attendees ($p=0.029$) |
| 17 | Zaki, 2014 | Saudi Arabia | September-November 2013 | Survey of 250 physicians | Randomized, cross-sectional survey | Conferences, drug samples | Favorable towards promotion |
| 18 | Orlowski et al., 1994 | USA | 1987-1989 | 10 physicians that were invited for a symposium and tracking the pharmacy inventory usage reports for these drugs before and after the symposia | Cohort | Conference travel | Significant increase in the prescribing pattern of drugs occurred following the symposia ($p<0.001$) |

For peer review only

| | | | | | | | |
|---|---|---|---|---|---|---|---|
| 19 | Scheffer et al., 2014 | Brazil | 2007-2009 | Survey of 300 physicians prescribing antiretroviral drugs | Cross-sectional | Interaction with representative, drug samples, journals | Frequency of interaction; the majority of (64%) of the physicians had multiple forms of interactions with PSR |
| 20 | Brett et al., 2003 | USA | Not reported | Questionnaire of 93 physicians in a medical school | Cross-sectional | Interaction with PSR | Impact on attitudes; most physicians believed that most of PSR activities do not pose major ethical problems |
| 21 | Gupta et al., 2016 | India | June-September 2014 | Survey of 81 physicians in single hospital | Cross-sectional | Interaction with representative, drug samples, journals | Impact on prescribing; 61.7% of participants think that PSR has an impact on their prescribing (P = 0.0001) |
| 22 | Morgan et al., 2006 | USA | March 2003 | Survey of 397 obstetrician-gynaecologists | Cross-sectional | Drug samples, promotional material, lunch | Impact on prescribing, positive attitudes; most respondents thought it's proper to accept drug samples (92%), lunch (77%), an anatomical model (75%) or a well-paid consultantship (53%) from PSR |
| 23 | Alosaimi | Saudi | 2012 | Survey of | Cross- | Interaction | Positive |

For peer review only

| | | | | | | | |
|---|---|---|---|---|---|---|---|
| | et al., 2014 | Arabia | | 659 physicians | sectional | with PSR | attitude towards PSR interaction |
| 24 | Chren et al., 1994 | USA | 1989-1990 | 40 case physicians and 80 control physicians | Case control | PSR interactions, honoraria, research | Increased prescription of company's drug after PSR interaction, honoraria and research (p<0.001, all) |
| 25 | Randall et al., 2005 | USA | October 2001 | Intervention group of physicians (n=18) that received education about PSR interaction and control group (n=14) | Controlled trial | Interaction with PSR | The majority of residents found the interactions and gifts useful. Compared to the comparison group, the intervention group significantly decreased the reported number of office supplies and noneducational gifts (p<0.05) |
| 26 | Caudil et al., 1996 | USA | Not reported | Survey of 446 primary care physicians | Cross-sectional | Interaction with PSR | Significant positive correlation between physician cost of prescribing and perceived credibility, availability, applicability, and use of information provided by PSR (p < 0.01) |

For peer review only

| | | | | | | | |
|---|---|---|---|---|---|---|---|
| 27 | Andaleeb et al., 1995 | USA | Not reported | 223 physicians in northwestern Pennsylvania | Cross-sectional | Interaction with PSR | Positive attitude towards PSR interaction |
| 28 | Reeder et al., 1993 | USA | 1991-1992 | 87 residents of emergency medicine | Cross-sectional | Interaction with PSR, gifts | Most participants believed that PSR interaction had no impact on their prescribing |
| 29 | Lichstein et al., 1992 | USA | January-March 1990 | 272 directors of internal medicine residency programs | Cross-sectional | Interaction with PSR | Most participants had a positive attitude towards PSR interactions |
| 30 | Brotzman et al., 1992 | USA | Not reported | Directors of 386 family practice residency programs | Cross-sectional | Interaction with PSR | Majority of programs do not have guidelines for interaction with PSR |
| 31 | Alssageer et al., 2012 | Libya | August-October 2010 | Survey of 608 physicians in public and private practice settings | Cross-sectional | Interaction with PSR, drug samples, printed materials | Positive attitude towards PSR interactions |
| 32 | Lieb & Brandtonies, 2010 | Germany | 2007 | Survey of 208 physicians (neurology, cardiology and general medicine) | Cross-sectional | Interaction with PSR, drug samples, printed materials, lunches | Frequency and impact on attitudes |
| 33 | Lieb & Scheurich, 2014 | Germany | 2010-2011 | Survey of 160 physicians in private and public practices | Cross-sectional | Interaction with representative, drug samples, printed materials, CME | High expenditure prescribing; avoidance of industry-sponsored CME is associated with more |

| | | | | | | | |
|---|---|---|---|---|---|---|---|
| | | | | | | | rational prescribing habits |
| 34 | Lieb & Koch, 2013 | Germany | May-July 2012 | Survey of 1038 medical students at 8 universities | Cross-sectional | Interaction with representative, drug samples, printed materials, lunches | Most participants have contact with the pharmaceutical company; 24.6% of the participants thought gifts would influence their future prescribing behavior, while 45.1% thought gifts would influence their classmates' future prescribing behavior ($p<0.001$) |
| 35 | Brown et al., 2015 | USA | 2008 and 2013 | 251 directors of family medicine residency programs | Cross-sectional | Interaction with PSR, gifts, lunches | Negative attitude towards PSR interactions |
| 37 | Rahmanaer et al., 2015 | Bangladesh | December 2008-January 2009 | Survey of 83 village physicians | Cross-sectional | Interaction with PSR | Impact on their prescribing |
| 38 | Lee & Begley, 2016 | USA | 2008 | Nationally representative survey of 4720 physicians | Cross-sectional | Gifts | Gifts were associated with lower perceived quality of patient care; an inverse relationship between the frequency of received gifts and the perceived quality of care was |

For peer review only

| | | | | | | | |
|---|---|---|---|---|---|---|---|
| | | | | | | | observed |
| 39 | Montastruc et al., 2014 | France | August-October 2011 | Survey among 631 medical residents | Cross-sectional | Interaction with representative | Most participants believed that PSR interaction had no impact on their prescribing; participants who had a more positive opinion were more frequently exposed to PSR ($p<0.001$) |
| 40 | Ketis & Kersnik, 2013 | Slovenia | October 2011 | 895 family physicians at the primary level of care | Cross-sectional | Interaction with PSR | Positive effect on knowledge; Participants value PSRs' selling and communication skills and trustworthiness highly |
| 41 | Hurley et al., 2014 | USA | 2010 | 3500 dermatologists | Cross-sectional | Free drug samples | Impact on their prescribing; the provision of samples with a prescription by dermatologists has been increasing over time, and this increase is correlated ($r = 0.92$) with the use of the branded generic drugs promoted by |

For peer review only

| | | | | | | | |
|---|---|---|---|---|---|---|---|
| | | | | | | | these sample |
| 42 | Makowska, 2014 | Poland | November-December 2008 | Survey of 382 physicians | Cross-sectional | Gifts | Positive attitude towards PSR interactions |
| 43 | Siddiqui et al., 2014 | Pakistan | Not reported | Questionnaires of 352 medical students | Cross-sectional | Interaction with representative | Positive attitude towards PSR interaction |
| 55 | Workneh BD et al., 2016 | Ethiopia | February-March 2015 | Survey of 90 physicians from public and private health facilities | Cross-sectional | Interaction with representative, gifts | Positive attitude towards industry, impact on prescribing behavior; Nearly half of the physicians reported that their prescribing decisions were influenced by PSR |
| 57 | Khan N et al., 2016 | Pakistan | Not reported | Questionnaires in 472 physicians | Cross-sectional | Interaction with representative, gifts | Positive attitude towards PSR interaction |
| 58 | Saito S et al. 2010 | Japan | January-March 2008 | 1417 physicians working in internal medicine, general surgery, orthopedic surgery, pediatrics, obstetrics-gynecology, psychiatry, and ophthalmology | National Survey | Interaction with industry, receipt of gifts, funds, CME, samples | Positive attitude towards PSR and gifts, value information from PSR, interactions higher with physicians who prefer to prescribe brand names |
| 59 | Ziegler MG et al. 1995 | USA | 1993 | 27 physicians working in public and | Survey | Accuracy of information provided by PSRs about | Incorrect information often provided by |

For peer review only

| | | | | | | | |
|---|---|---|---|---|---|---|---|
| | | | | private hospitals | | drugs | speakers goes unnoticed by physicians |
| 60 | Lurie N et al., 1990 | USA | Not reported | 240 internal medicine faculty physicians in academic medical centers | Survey | Effect of interaction with PSR, free meals, honoraria and research support | Impact on prescribing behavior and formulary change requests |
| 62 | DeJong C et al., 2016 | USA | August-September 2013 | 279.669 physicians who wrote Medicare prescriptions in any of 4 drug classes: statins, cardioselective β-blockers, angiotensin-converting enzyme inhibitors and angiotensin-receptor blockers (ACE inhibitors and ARBs), and selective serotonin and serotonin-norepinephrine reuptake inhibitors (SSRIs and SNRIs) Physicians | Cross-sectional | Industry sponsored meals | Receipt of industry-sponsored meals was associated with an increased rate of brand name prescription. |
| 63 | Yeh JS et al., 2016 | USA | 2011 | All licensed Massachusetts physicians who wrote prescriptions for statins paid for | Cross-sectional | Effect of industry payment on prescription of branded drugs for cholesterol control | Payment for meals and educational programs increased prescription of brand name |

For peer review only

| | | | | | | | |
|---|---|---|---|---|---|---|---|
| | | | | under the Medicare drug benefit in 2011 (n=2444) | | | statins. |
| 65 | Bowman MA et al., 1988 | USA | Not reported | 121 physician attendees | Self report survey | Effect of CME on prescribing behavior | Sponsoring company's drugs were favored during prescription |
| 66 | Fischer MA et al., 2009 | USA | November 2006-March 2007 | Multi-disciplinary focus groups with 61 physicians | Survey | Effect of industry marketing strategies on prescription and cognitive dissonance of physicians | Most participants reported no PSR impact on their prescribing, value to have ability to evaluate information of PSRs |
| 67 | Chimonas S et al., 2007 | USA | June 2004 | Six focus groups in 32 academic and community physicians | Survey | PSR interactions | Positive attitude towards PSR interaction |
| 72 | Yeh JS et al., 2014 | USA | Not reported | 1610 US medical students | Cross-sectional | Interaction with representative, gifts, lunches | Policies separating students from representatives reduced number of interactions |
| 73 | Larkin I et al., 2014 | USA | January 2006-June 2009 | Pediatricians, child & adolescent psychiatrists in five medical centers | Survey | Interaction with PSR | Anti detailing policies reduced the prescription of off-label antidepressants and anti psychotics for children |
| 74 | Esmaily HM et al., 2010 | Iran | Not reported | 112 general physicians were randomized in two groups: 1) outcome- | Randomized trial | Effect of outcome and retinal prescribing | Rational prescribing improved in some of the important outcome-based |

For peer review only

| | | | | | | | |
|---|---|---|---|---|---|---|---|
| | | | | based educational intervention for rational prescribing and 2) concurrent CME program in the field of rational prescribing | | | indicators. No difference between two arms of the study |
| 76 | Parikh K et al., 2016 | USA | 2014 | descriptive, cross-sectional analysis of Open Payments data and 9 638 825 payments to physicians and pediatricians from January 1 to December 31, 2014 | Cross-sectional | Comparison of PSR interactions between pediatricians and other specialists; among subspecialities of pediatrics. | Pediatricians get fewer gifts from PSR than internists. There is variation among sub specialities for extent of interaction. |
| 78 | Chressanthis GA et al. 2012 | USA | Not reported | Clinical decisions of 72,114 physicians were statistically analyzed using prescription data | Survey | Effect of restricting PSRs on clinical practice and knowledge | Restricting PSRs affected information flow about drugs, both negative and positive. |

We excluded 2000 records as they were not relevant (n = 1641), not original research (n=269), about medical students (n=4) and non-medical (e.g. ecological, econometric; n=86).

*Characteristics of included studies*

The identified studies were published between 1992 and August 2016. Most of the studies included were cross-sectional studies[1,9-13,19,21,22,31-55]. Only two studies were cohort studies[56, 57], three were randomized trials[58-60] and one study was a case-control study[61].

*Extent of interactions between physicians and the pharmaceutical industry*

We found that PSR interactions are a regular feature in the daily lives of physicians across the world [9-11,13,42,50]. Most of the attending physicians and residents have at least one interaction

For peer review only

with industry representatives per month [10,21,22,36,42]. The frequency of interactions or gifts offered and accepted varies with private versus public hospital setting and the position of the physicians in the medical hierarchy [10,13,31,38,42,43,50,58,62]. Junior residents received twice as much free drug samples from PSR interactions than senior residents [10]. PSR interactions were significantly higher at the beginning of residency[13]. The majority of program directors of internal medicine residencies in the USA allowed PSRs to meet with residents during working hours and permitted PSR sponsorship of conferences[40]. Attending physicians and physician specialists had more PSR interactions and received higher numbers of medical samples and promotional material than residents[9,42]. Participants working in private practice alone or in both sectors were more likely to receive gifts than physicians working in the public sector [38,42,50].. Most common gifts received were medical samples[9,21,22,31,36,37,42,63], promotional material[9,34,42] invitations for dinners[9], invitations for CMEs[22,34], scientific journals[34] and free lunches[21,37].

*Perspectives of physicians towards PSR interactions*

We found that physicians have a positive attitude towards PSRs[1, 13, 19, 20, 22, 31, 32, 40, 49, 58, 64] Physicians perceived PSRs as important sources of education and funding[10, 22, 32, 43, 45, 46]; while some studies reporting skeptical attitudes about the contribution of PSRs towards teaching and education[21, 36, 39, 40, 49]. Conference registration fees, informational luncheons, sponsorship of departmental journal clubs, anatomical models, and free drug samples were considered as appropriate gifts[19, 39, 51, 58]. Most of the physicians considered pharmaceutical information provided by PSRs, industry sponsored conferences and CME events as important instruments for enhancing their scientific knowledge[22, 32, 45, 46]. Compared to senior residents, significantly more junior residents felt that pharmaceutical representatives have a valuable teaching role [10].

Most of the physicians considered themselves immune to the influence of gifts[1, 10, 32, 33, 35, 37, 43, 59]. Most studies found that physicians do not believe that PSR interactions impact their prescribing behavoir,[1, 9-13, 65, 66], while other studies found that there was some extent of influence[21, 22, 34, 36, 37, 39, 43]. In addition, physicians considered their colleagues more susceptible than themselves to PSR marketing strategies[1, 20, 21, 37, 43]. There was a strong correlation between the amount of gifts and the belief that PSR interactions did not influence their prescribing behavior [10].

For peer review only

*Gifts*

We found that better scores on knowledge and attitudes were significantly associated with fewer interactions with representatives and their gifts[19]. Most common gifts received were medical samples[9, 21, 22, 31, 36, 37, 42-44, 47], promotional material[9, 34, 42, 67] invitations for dinners[9] and scientific journals[34]

*Drug samples*

Most of the physicians who accepted drug samples had a positive attitude towards the pharmaceutical representatives[9, 21, 22, 31, 36, 37, 42, 43]. Accepting samples lead to higher branded drug prescription rather than generic prescribing [22,47].

*Pharmaceutical representative speakers*

Sponsored lectures/symposia of pharmaceutical companies influenced behavior of the attendees leading to the attendees prescribing more drugs from the sponsoring companies without sufficient evidence supporting superiority of those drugs[56,57]. The majority of attending physicians failed to identify inaccurate information about the company drug[18].

*Honoraria and Research Funding*

Physicians who received money to attend pharmaceutical symposia or to perform research requested formulary addition of the company's drug more often than other physicians, This association was independent of many confounding factors[61] (Table 2). Brief encounters with PSRs and receipt of honoraria or research support were predictors of faculty requested change in hospital formulary[68].

*Conference travel*

Pharmaceutical company sponsored conference travels to touristic locations have quantifiable impact on the prescribing rational of attendees. A significant increase (three times) in the prescribing rate of two company drugs was observed after the physicians attended a company sponsored symposium with all their expenses covered. Despite this significant difference in the prescribing patterns, physicians insisted there was no impact on their prescribing behaviour.[57]

*Industry paid lunches*

For peer review only

Most physicians received invitations for dinners[9] and free lunches[10, 21, 35, 43] . Clerks, interns and junior residents attended more company sponsored lunches than senior residents [10]. Pharmaceutical companies also sponsored departmental lunches during journal clubs[39]. There was no significant association between attending industry paid lunches[37] and dinners[9] and formulary request for that company's drug (Table 2).

| # | Attitudes | Prescribing behavior | Knowledge | Formulary requests | Quality of Evidence (GRADE) |
|---|---|---|---|---|---|
| **Gifts** | Receiving higher number of gifts associated with belief that PRs have no impact on their prescribing behaviour [1,14,39] | - | - | - | **Moderate** |
| **Drug samples** | Positive attitude towards the drug industry and the representatives[11,21,34] | Higher prescription of the company drug[21, 41] | - | - | **High** |
| **Pharmaceutical representative speakers** | - | Irrational prescribing [16, 18, 34] | Inability to identify false claims[16] | Increased prescription of sponsor's drug[24] | **High** |
| **Honoraria and Research Funding** | Positive attitude towards sponsor's drug [60] | - | - | Increased prescription of sponsor's drug[24] | **Low** |
| **Conference travel** | - | Significant increase in prescribing of sponsor drug[18] | - | Increased prescription of sponsor's drug[24] | **Low** |
| **Industry paid lunches** | Positive attitude towards sponsor's drug [14, 34] | Significant increase in prescribing of sponsor drug[62] | - | Increased formulary request for company drug [11,21] | **High** |
| **CME sponsorship** | Positive attitude towards sponsor's drug [24, 65] | Avoidance of industry-sponsored CME associated with more rational prescribing habits [33] | | | **Moderate** |

For peer review only

| | | | | | |
|---|---|---|---|---|---|
| **Interaction with PRS** | Positive attitude towards PSR drugs [1,11,14,58] | Higher prescription of the company drug[24] | Positive correlation between the physicians' prescribing cost and the information provided by the drug representative during the interaction[26] | Increased prescription of sponsor's drug[24] | **High** |

However, there was a significant association between attending industry paid lunches and increased prescription of branded drugs [52,53, 69].

*CME sponsorship*

Physicians who attended company sponsored CME events had more positive attitudes towards and inclination to prescribe the branded drugs[28, 34, 43, 67, 70-72]. We found that physicians who refused CME sponsorship were seen to prescribe higher proportion of generics and lower expenditure medicines when compared to physicians who attended CMEs[22].

**Discussion**

We report that there is widespread interaction between the pharmaceutical industry and physicians[9-11, 13, 42, 50]. Interactions are in the form of personal communications, free gifts such as drug samples, sponsored meals, sponsored conference travel, funding for research and CMEs and honoraria[9, 21, 22, 31, 36, 42]. The frequency of these interactions is comparable between residents and physicians[10, 21, 22, 36, 42]. However, the amount and type of gifts vary with the position of the physician in medical hierarchy, specialization and location of practice[10, 13, 31, 38, 42, 43, 50, 58, 62]. In general, trainees (residents, interns) are treated with more drug samples, stationery items and free meals than senior physicians[10,13]. Senior physicians usually avail of sponsored conferences/ trips, research funding, honoraria and CME events. The extent of these interactions varies with academic versus non-academic institutions: non-academic hospitals record more interactions than others[31, 38, 42, 50, 55]. The majority of the physicians do not believe that they are affected by PSR interactions[1, 10, 32, 33, 35, 37, 43, 59]. However, a sizeable percentage in various surveys responded in the affirmative when asked whether they thought that their peers are vulnerable[1, 20, 21, 37, 43].

For peer review only

We observe that there is a positive correlation between acceptance of gifts and physicians' urge to reciprocate favorably towards the benefactor[12,22,47, 73, 74] Considering that physicians have a social contract with society at large to provide unbiased and altruistic service, this is an alarming observation. In 2005 a joint report by the WHO and Health Action International (HAI) reported on interventions to counter promotional activities.[75] The evidence presented in that report was not eligible for our systematic review, mostly because it related to interventions on students or residents. Nevertheless, the findings suggested that interventions such as industry self-regulation and guidelines for sales representatives are not effective, while education about drug promotion might influence physician attitudes[76-78].

*Policies and educational intervention*

The relationship of physicians with patients is of a fiduciary nature. Hence activities that might affect that relationship by altering physicians' clinical behavior are not acceptable. Physician-pharmaceutical industry and PSR interactions may put the trust of patients in physicians at risk. Interaction with pharmaceutical industry and PSRs begins early in the physicians' career. Trainees are exposed to pharmaceutical industry marketing and promotional techniques from the initial years of their medical education, which impact their prescribing behavior in future. Overall, trainees, i.e., residents and interns, are more vulnerable to pharmaceutical industry and PSR interactions than senior physicians[11, 41, 62] Physicians are susceptible to pharmaceutical industry and PSR interactions, which influences their clinical decision-making leading to greater prescriptions of branded drugs over low cost generic medicines and increasing healthcare cost[22, 47, 52, 53, 69] Therefore, there is need to institute and implement stringent policies curtailing physician-pharmaceutical industry and PSR relationships, as well as educational programs to increase awareness. Previous reports have indicated that implementing policies and conducting educational programs are effective in increasing awareness of physician's attitudes towards pharmaceutical industry and PSR interactions[54, 59, 60, 76, 79-83]

*Strengths and Limitations of the study*

A major strength of this study is that is a large up-to-date systematic review of studies exploring the effects of physician and pharmaceutical industry representative interactions and residents in different settings (e.g. academic, primary care). Another strength of this study is the use of Cochrane and GRADE methodologies for conducting a review and assessing the quality of the studies. Moreover, we performed an extensive search in 3 databases and the

For peer review only

grey literature. Some of the limitations of this review are related to the included studies, as some did not provide evidence for the significance of their findings or had varying study designs and outcomes, which made it impossible to conduct a meta-analysis. Also, the included studies were subject to risk of bias related to the lack of validity of outcome measurement, and inadequate handling of significant potential confounders.

*Future implications*

Pharmaceutical industry and PSR interactions compromise the objectivity of the physicians. Educating physicians and increasing regulation of pharmaceutical industry and PSR interactions may lower the likelihood of prescribing new non-superior industry drugs and irrational prescription behavior. Further studies are required to evaluate the impact of pharmaceutical industry and PSR interactions on physicians over time and the benefits of various intervention based education programs on the clinical and ethical behavior of the physicians.

For peer review only

**References**


1. Steinman, M.A., M.G. Shlipak, and S.J. McPhee, *Of principles and pens: attitudes and practices of medicine housestaff toward pharmaceutical industry promotions.* The American Journal of Medicine, 2001. **110**(7): p. 551-557.
2. Wazana, A., *Physicians and the Pharmaceutical Industry.* JAMA, 2000. **283**(3): p. 373.
3. Wolfe, S.M., *Why do american drug companies spend more than $12 billion a year pushing drugs? Is it education or promotion?* Journal of General Internal Medicine, 1996. **11**(10): p. 637-639.
4. Wall, L.L. and D. Brown, *The High Cost of Free Lunch.* Obstetrics & Gynecology, 2007. **110**(1): p. 169-173.
5. Chressanthis, G.A., et al., *Determinants of pharmaceutical sales representative access limits to physicians.* Journal of Medical Marketing, 2014. **14**(4): p. 220-243.
6. International, K., *Rising costs of litigation in pharmaceutical industry.* Issues Monitor-Pharmaceuticals, 2011. **9**(14): p. 1-3.
7. MedAdNews, *Table insert on the top 50 pharma companies. Data provided by Cegedim Strategic Data.* 2010. **September**.
8. Mack J, *Pharma promotional spending in 2013.* Pharma marketing news, 2014. **13**(5).
9. De Ferrari, A., et al., *Attitudes and Relationship between Physicians and the Pharmaceutical Industry in a Public General Hospital in Lima, Peru.* PLoS ONE, 2014. **9**(6): p. e100114.
10. Hodges B. *Interactions with the pharmaceutical industry: experiences and attitudes of psychiatry residents, interns and clerks.* CMAJ: Canadian Medial Association Journal, 1995. **153**(5): p. 553.

11. Riese, F., et al., *Pharmaceutical industry interactions of psychiatric trainees from 20 European countries.* European Psychiatry, 2015. **30**(2): p. 284-290.
12. Lee, D. and C.E. Begley, *Physician report of industry gifts and quality of care.* Health Care Management Review, 2016. **41**(3): p. 275-283.
13. Montastruc, F., et al., *Interactions between Medical Residents and Drug Companies: A National Survey after the Mediator® Affair.* PLoS ONE, 2014. **9**(10): p. e104828.
14. Fugh-Berman, A. and S. Ahari, *Following the Script: How Drug Reps Make Friends and Influence Doctors.* PLoS Medicine, 2007. **4**(4): p. e150.
15. Kesselheim, A.S., M.M. Mello, and D.M. Studdert, *Strategies and Practices in Off-Label Marketing of Pharmaceuticals: A Retrospective Analysis of Whistleblower Complaints.* PLoS Medicine, 2011. **8**(4): p. e1000431.
16. Mulinari, S., *Unhealthy marketing of pharmaceutical products: An international public health concern.* Journal of Public Health Policy, 2016. **37**(2): p. 149-159.
17. Othman, N., et al., *Medicines information provided by pharmaceutical representatives: a comparative study in Australia and Malaysia.* BMC Public Health, 2010. **10**(1).
18. Ziegler, M.G., *The Accuracy of Drug Information From Pharmaceutical Sales Representatives.* JAMA: The Journal of the American Medical Association, 1995. **273**(16): p. 1296.
19. Alosaimi FD, A.K.A., Qadi M, Albahlal A, Alabdulkarim Y, Alabduljabbar M, Alqahtani F., *Physicians' attitudes towards interaction with the pharmaceutical industry/Attitudes des médecins par rapport á l'interaction avec l'industrie pharmaceutique.* Eastern Mediterranean Health Journal, 2014. **20**(12): p. 812.
20. Andaleeb, S.S. and R.F. Tallman, *Physician attitudes toward pharmaceutical sales representatives.* Health Care Management Review, 1995. **20**(3): p. 68-76.
21. Lieb K, B.S., *A survey of German physicians in private practice about contacts with pharmaceutical sales representatives.* Dtsch Arztebl Int., 2012. **107**(22): p. 392-8.
22. Lieb, K. and A. Scheurich, *Contact between Doctors and the Pharmaceutical Industry, Their Perceptions, and the Effects on Prescribing Habits.* PLoS ONE, 2014. **9**(10): p. e110130.
23. Francer, J., et al., *Ethical pharmaceutical promotion and communications worldwide: codes and regulations.* Philosophy, Ethics, and Humanities in Medicine, 2014. **9**(1): p. 7.
24. Canada., C.o.f.p.o.C.R.c.o.p.a.s.o., *Guide tot enhancing referrals and consultations between physicians.* College of Family Physicians of Canada., 2009.
25. Grande, D., *Limiting the Influence of Pharmaceutical Industry Gifts on Physicians: Self-Regulation or Government Intervention?* Journal of General Internal Medicine, 2009. **25**(1): p. 79-83.
26. Brennan, T.A., et al., *Health Industry Practices That Create Conflicts of Interest.* JAMA, 2006. **295**(4): p. 429.
27. Spurling, G.K., et al., *Information from Pharmaceutical Companies and the Quality, Quantity, and Cost of Physicians' Prescribing: A Systematic Review.* PLoS Medicine, 2010. **7**(10): p. e1000352.
28. J., Lexchin., *Interactions between physicians and the pharmaceutical industry: what does the literature say? .* CMAJ: Canadian Medial Association Journal, 1993. **14**(10): p. 1401-7.
29. Wazana, A., et al., *Using the Literature in Developing McGill's Guidelines for Interactions between Residents and the Pharmaceutical Industry.* Academic Medicine, 2004. **79**(11): p. 1033-1040.

For peer review only

30. Guyatt, G., et al., *GRADE guidelines: 1. Introduction—GRADE evidence profiles and summary of findings tables.* Journal of Clinical Epidemiology, 2011. **64**(4): p. 383-394.
31. Thomson AN, C.B., Barham PM., *Attitudes of general practitioners in New Zealand to pharmaceutical representatives.* Br J Gen Pract., 1994. **44**(382): p. 220-3.
32. Kamal, S., et al., *Perceptions and Attitudes of Egyptian Health Professionals and Policy-Makers towards Pharmaceutical Sales Representatives and Other Promotional Activities.* PLOS ONE, 2015. **10**(10): p. e0140457.
33. Gibbons, R.V., et al., *A comparison of physicians' and patients' attitudes toward pharmaceutical industry gifts.* Journal of General Internal Medicine, 1998. **13**(3): p. 151-154.
34. Scheffer, M.C., *Interaction between pharmaceutical companies and physicians who prescribe antiretroviral drugs for treating AIDS.* Sao Paulo Medical Journal, 2014. **132**(1): p. 55-60.
35. Brett, A.S., W. Burr, and J. Moloo, *Are Gifts From Pharmaceutical Companies Ethically Problematic?* Archives of Internal Medicine, 2003. **163**(18): p. 2213.
36. Gupta, S., R. Nayak, and R. Sivaranjani, *A study on the interactions of doctors with medical representatives of pharmaceutical companies in a Tertiary Care Teaching Hospital of South India.* Journal of Pharmacy and Bioallied Sciences, 2016. **8**(1): p. 47.
37. Morgan, M.A., *Interactions of doctors with the pharmaceutical industry.* Journal of Medical Ethics, 2006. **32**(10): p. 559-563.
38. Caudill, T.S., *Physicians, pharmaceutical sales representatives, and the cost of prescribing.* Archives of Family Medicine, 1996. **5**(4): p. 201-206.
39. Reeder, M., J. Dougherty, and L.J. White, *Pharmaceutical representatives and emergency medicine residents: A national survey.* Annals of Emergency Medicine, 1993. **22**(10): p. 1593-1596.
40. Lichstein PR, T.R., O'Brien K., *Impact of pharmaceutical company representatives on internal medicine residency programs: a survey of residency program directors.* Archives of Internal Medicine, 1992. **152**(5): p. 1009-13.
41. Brotzman GL, M.D., *Policies regulating the activities of pharmaceutical representatives in residency programs.* Journal of Family Practice, 1992. **34**(1): p. 54-8.
42. Alssageer, M. and S. Kowalski, *A survey of pharmaceutical company representative interactions with doctors in Libya.* Libyan Journal of Medicine, 2012. **7**(1): p. 18556.
43. Lieb K, K.C., *Medical students' attitudes to and contact with the pharmaceutical industry: a survey at eight German University hopitals.* Dtsch. Arztebl. Int., 2013. **110**(35): p. 584-90.
44. Brown SR, E.D., Berman AF., *Pharmaceutical industry interactions in family medicine residencies decreased between 2008 and 2013: a CERA study.* Fam. Med., 2015. **47**(4): p. 279-82.
45. Rahmana MH, A.S., Tuddenhama S, Petoa H, Iqbalb M, Bhuiyab A, Peters DH., *What do they do? Interactions between village doctors and medical representatives in Chakaria, Banladesh.* Int. Health., 2015. **7**(1): p. 266-71.
46. KlemencKetis, Z. and J. Kersnik, *Which Pharmaceutical Sales Representatives' Features do Slovenian Family Physicians Value?* Acta Informatica Medica, 2013. **21**(4): p. 257.
47. Hurley, M.P., R.S. Stafford, and A.T. Lane, *Characterizing the Relationship Between Free Drug Samples and Prescription Patterns for Acne Vulgaris and Rosacea.* JAMA Dermatology, 2014. **150**(5): p. 487.


For peer review only

48. Makowska, M., *Interactions between Doctors and Pharmaceutical Sales Representatives in a Former Communist Country.* Cambridge Quarterly of Healthcare Ethics, 2014. **23**(03): p. 349-355.
49. Siddiqui, U.T., et al., *Attitudes of medical students towards incentives offered by pharmaceutical companies- perspective from a developing nation- a cross sectional study.* BMC Medical Ethics, 2014. **15**(1).
50. Workneh, B.D., et al., *Influence of Medical Representatives on Prescribing Practices in Mekelle, Northern Ethiopia.* PLOS ONE, 2016. **11**(6): p. e0156795.
51. Khan, N., et al., *Perceptions and Attitudes of Medical Sales Representatives (MSRs) and Prescribers Regarding Pharmaceutical Sales Promotion and Prescribing Practices in Pakistan.* Journal of Young Pharmacists, 2016. **8**(3): p. 244-250.
52. DeJong, C., et al., *Pharmaceutical Industry–Sponsored Meals and Physician Prescribing Patterns for Medicare Beneficiaries.* JAMA Internal Medicine, 2016. **176**(8): p. 1114.
53. Yeh, J.S., et al., *Association of Industry Payments to Physicians With the Prescribing of Brand-name Statins in Massachusetts.* JAMA Internal Medicine, 2016. **176**(6): p. 763.
54. Yeh, J.S., et al., *Association of Medical Students' Reports of Interactions with the Pharmaceutical and Medical Device Industries and Medical School Policies and Characteristics: A Cross-Sectional Study.* PLoS Medicine, 2014. **11**(10): p. e1001743.
55. Parikh, K., W. Fleischman, and S. Agrawal, *Industry Relationships With Pediatricians: Findings From the Open Payments Sunshine Act.* PEDIATRICS, 2016. **137**(6): p. e20154440-e20154440.
56. Spingarn, R.W., J.A. Berlin, and B.L. Strom, *When pharmaceutical manufacturers' employees present grand rounds, what do residents remember?* Academic Medicine, 1996. **71**(1): p. 86-8.
57. Orlowski, J.P. and L. Wateska, *The Effects of Pharmaceutical Firm Enticements on Physician Prescribing Patterns: there's no such thing as a free lunch.* Chest, 1992. **102**(1): p. 270-273.
58. Zaki, N.M., *Pharmacists' and physicians' perception and exposure to drug promotion: A Saudi study.* Saudi Pharmaceutical Journal, 2014. **22**(6): p. 528-536.
59. Randall, M., Rosenbaum JR, Rohrbaugh RM, Rosenheck RA., *Attitudes and Behaviors of Psychiatry Residents Toward Pharmaceutical Representatives Before and After an Educational Intervention.* Academic Psychiatry, 2005. **29**(1): p. 33-39.
60. Esmaily, H.M., et al., *Can rational prescribing be improved by an outcome-based educational approach? A randomized trial completed in Iran.* Journal of Continuing Education in the Health Professions, 2010. **30**(1): p. 11-18.
61. Chren MM, L.C., *Physicians' Behavior and Their Interactions With Drug Companies: a controlled study of physicians who requested additions to a hospital drug formulary.* JAMA, 1994. **271**(9): p. 684.
62. Zipkin, D.A. and M.A. Steinman, *Interactions between pharmaceutical representatives and doctors in training.* Journal of General Internal Medicine, 2005. **20**(8): p. 777-786.
63. McMurray, R.J., *Gifts to Physicians From Industry.* JAMA: The Journal of the American Medical Association, 1991. **265**(4): p. 501.
64. Lotfi, T., et al., *Knowledge, beliefs and attitudes of physicians in low and middle-income countries regarding interacting with pharmaceutical companies: a systematic review.* BMC Health Services Research, 2016. **16**(1).
65. Fischer, M.A., et al., *Prescribers and Pharmaceutical Representatives: Why Are We Still Meeting?* Journal of General Internal Medicine, 2009. **24**(7): p. 795-801.

For peer review only

66. Chimonas, S., T.A. Brennan, and D.J. Rothman, *Physicians and Drug Representatives: Exploring the Dynamics of the Relationship.* Journal of General Internal Medicine, 2007. **22**(2): p. 184-190.
67. Saito, S., K. Mukohara, and S. Bito, *Japanese Practicing Physicians' Relationships with Pharmaceutical Representatives: A National Survey.* PLoS ONE, 2010. **5**(8): p. e12193.
68. Lurie, N., et al., *Pharmaceutical representatives in academic medical centers.* Journal of General Internal Medicine, 1990. **5**(3): p. 240-243.
69. Steinbrook, R., *Industry Payments to Physicians and Prescribing of Brand-name Drugs.* JAMA Internal Medicine, 2016. **176**(8): p. 1123.
70. Bowman, M.A. and D.L. Pearle, *Changes in drug prescribing patterns related to commercial company funding of continuing medical education.* Journal of Continuing Education in the Health Professions, 1988. **8**(1): p. 13-20.
71. Grundy Q, B.L., Malone R 2013, *Interactions between non-physician clinicians and industry: a systematic review.* PLoS Med. , 2013. **11**(Nov 26).
72. Dixon, D., et al., *Controlling Quality in CME/CPD by Measuring and Illuminating Bias.* Journal of Continuing Education in the Health Professions, 2011. **31**(2): p. 109-116.
73. Silverman, E., *Everything you need to know about the Sunshine Act.* BMJ, 2013. **347**(jul26 1): p. f4704-f4704.
74. Shalowitz, D.I., M.A. Spillman, and M.A. Morgan, *Interactions with industry under the Sunshine Act: an example from gynecologic oncology.* American Journal of Obstetrics and Gynecology, 2016. **214**(6): p. 703-707.
75. Norris P, H.A., Lexchin J, et al., *Drug promotion—what we know, what we have yet to learn—reviews of materials in the WHO/HAI database on drug promotion.* EDM Research Series, 2005(No. 032.).
76. Carroll, A.E., et al., *To What Extent Do Educational Interventions Impact Medical Trainees' Attitudes and Behaviors Regarding Industry-Trainee and Industry-Physician Relationships?* PEDIATRICS, 2007. **120**(6): p. e1528-e1535.
77. Birkhahn, R.H., et al., *A Review of the Federal Guidelines That Inform and Influence Relationships Between Physicians and Industry.* Academic Emergency Medicine, 2009. **16**(8): p. 776-781.
78. Chressanthis, G.A., et al., *Can Access Limits on Sales Representatives to Physicians Affect Clinical Prescription Decisions? A Study of Recent Events With Diabetes and Lipid Drugs.* The Journal of Clinical Hypertension, 2012. **14**(7): p. 435-446.
79. Sergeant MD, H.P., Godwin M, Walker DM, McHenry P., *Interactions with the pharmaceutical industry: a survey of family medicine residents in Ontario.* CMAJ: Canadian Medial Association Journal, 1996. **155**(9): p. 1243.
80. T Montague, B., A.H. Fortin Vi, and J. Rosenbaum, *A systematic review of curricula on relationships between residents and the pharmaceutical industry.* Medical Education, 2008. **42**(3): p. 301-308.
81. Larkin, I., et al., *Restrictions On Pharmaceutical Detailing Reduced Off-Label Prescribing Of Antidepressants And Antipsychotics In Children.* Health Affairs, 2014. **33**(6): p. 1014-1023.
82. R., P., *Guidelines for faculty involvement in commercially supported continuing medical-education.* Academic Medicine, 1992.
83. Greenland, P., *Time for the Medical Profession to Act.* Archives of Internal Medicine, 2009. **169**(9): p. 829.

**Legends**

For peer review only

**Figure 1:** PRISMA flow diagram showing search strategy and included studies

**Table 1:** Characteristics of included studies

**Table 2:** Impact of physician-pharmaceutical industry interaction on physician

**The Corresponding Author has the right to grant on behalf of all authors and does grant on behalf of all authors, a worldwide licence (http://www.bmj.com/sites/default/files/BMJ%20Author%20Licence%20March%2020 13.doc) to the Publishers and its licensees in perpetuity, in all forms, formats and media (whether known now or created in the future), to i) publish, reproduce, distribute, display and store the Contribution, ii) translate the Contribution into other languages, create adaptations, reprints, include within collections and create summaries, extracts and/or, abstracts of the Contribution and convert or allow conversion into any format including without limitation audio, iii) create any other derivative work(s) based in whole or part on the on the Contribution, iv) to exploit all subsidiary rights to exploit all subsidiary rights that currently exist or as may exist in the future in the Contribution, v) the inclusion of electronic links from the Contribution to third party material where-ever it may be located; and, vi) licence any third party to do any or all of the above. All research articles will be made available on an Open Access basis (with authors being asked to pay an open access fee—see http://www.bmj.com/about-bmj/resources-authors/forms-policies-and-checklists/copyright-open-access-and-permission-reuse).**
**The terms of such Open Access shall be governed by a Creative Commons licence—details as to which Creative Commons licence will apply to the research article are set out in our worldwide licence referred to above.**

For peer review only

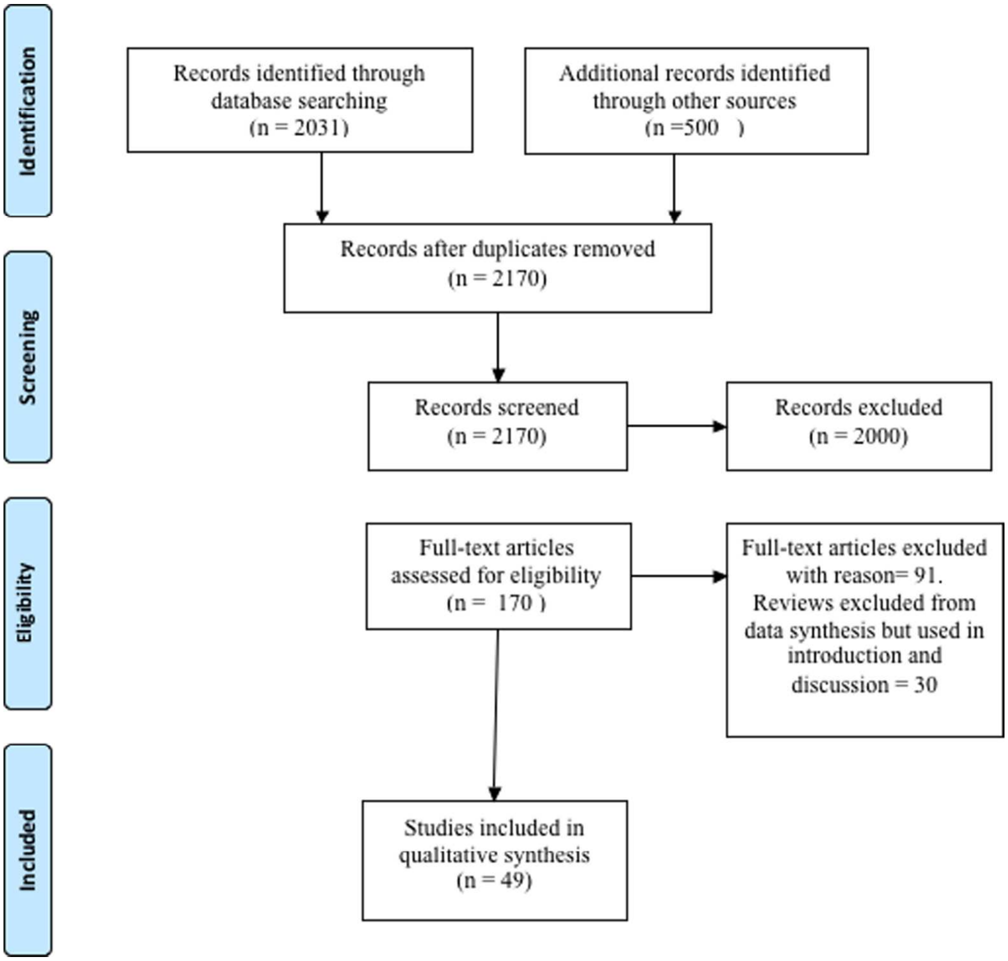


Flow diagram of study selection.

only

Search strategy for PubMed search engine of Medline

In an attempt to find all related literature on the topic, studies related to physician-pharmaceutical representative interactions that affect the prescribing behavior of the physicians were identified through computerized searches using, but not limited to, the following subject headings and text words in PubMed from 1992 to 2016.

1. Physician interactions with pharmaceutical industry
2. Physician attitude towards pharmaceutical representatives
3. Behavior of physicians towards pharmaceutical representatives
4. Gifts AND physician AND pharmaceutical representatives
5. Honoraria AND physician AND pharmaceutical representatives
6. Continuing medical education AND physician AND pharmaceutical representatives
7. Research funding AND physician AND pharmaceutical representatives
8. Conference travel AND physician AND pharmaceutical representatives
9. Industry sponsored meals AND physician behavior

For peer review only

# PRISMA 2009 Checklist

| Section/topic | # | Checklist item | Reported on page # |
|---|---|---|---|
| **TITLE** | | | |
| Title | 1 | Identify the report as a systematic review, meta-analysis, or both. | 1 |
| **ABSTRACT** | | | |
| Structured summary | 2 | Provide a structured summary including, as applicable: background; objectives; data sources; study eligibility criteria, participants, and interventions; study appraisal and synthesis methods; results; limitations; conclusions and implications of key findings; systematic review registration number. | 2 |
| **INTRODUCTION** | | | |
| Rationale | 3 | Describe the rationale for the review in the context of what is already known. | 3 |
| Objectives | 4 | Provide an explicit statement of questions being addressed with reference to participants, interventions, comparisons, outcomes, and study design (PICOS). | 3 |
| **METHODS** | | | |
| Protocol and registration | 5 | Indicate if a review protocol exists, if and where it can be accessed (e.g., Web address), and, if available, provide registration information including registration number. | 4 |
| Eligibility criteria | 6 | Specify study characteristics (e.g., PICOS, length of follow-up) and report characteristics (e.g., years considered, language, publication status) used as criteria for eligibility, giving rationale. | 3 |
| Information sources | 7 | Describe all information sources (e.g., databases with dates of coverage, contact with study authors to identify additional studies) in the search and date last searched. | 4 |
| Search | 8 | Present full electronic search strategy for at least one database, including any limits used, such that it could be repeated. | 4 |
| Study selection | 9 | State the process for selecting studies (i.e., screening, eligibility, included in systematic review, and, if applicable, included in the meta-analysis). | 3/4 |
| Data collection process | 10 | Describe method of data extraction from reports (e.g., piloted forms, independently, in duplicate) and any processes for obtaining and confirming data from investigators. | 4 |
| Data items | 11 | List and define all variables for which data were sought (e.g., PICOS, funding sources) and any assumptions and simplifications made. | 4 |
| Risk of bias in individual studies | 12 | Describe methods used for assessing risk of bias of individual studies (including specification of whether this was done at the study or outcome level), and how this information is to be used in any data synthesis. | 3 |
| Summary measures | 13 | State the principal summary measures (e.g., risk ratio, difference in means). | 3 |
| Synthesis of results | 14 | Describe the methods of handling data and combining results of studies, if done, including measures of consistency (e.g., $I^2$) for each meta-analysis. | 3 |

For peer review only - http://bmjopen.bmj.com/site/about/guidelines.xhtml

# PRISMA 2009 Checklist

| Section/topic | # | Checklist item | Reported on page # |
|---|---|---|---|
| Risk of bias across studies | 15 | Specify any assessment of risk of bias that may affect the cumulative evidence (e.g., publication bias, selective reporting within studies). | 3 |
| Additional analyses | 16 | Describe methods of additional analyses (e.g., sensitivity or subgroup analyses, meta-regression), if done, indicating which were pre-specified. | 3 |
| **RESULTS** | | | |
| Study selection | 17 | Give numbers of studies screened, assessed for eligibility, and included in the review, with reasons for exclusions at each stage, ideally with a flow diagram. | 5 |
| Study characteristics | 18 | For each study, present characteristics for which data were extracted (e.g., study size, PICOS, follow-up period) and provide the citations. | 5-8 |
| Risk of bias within studies | 19 | Present data on risk of bias of each study and, if available, any outcome level assessment (see item 12). | 5-8 |
| Results of individual studies | 20 | For all outcomes considered (benefits or harms), present, for each study: (a) simple summary data for each intervention group (b) effect estimates and confidence intervals, ideally with a forest plot. | 5-8 |
| Synthesis of results | 21 | Present results of each meta-analysis done, including confidence intervals and measures of consistency. | NA |
| Risk of bias across studies | 22 | Present results of any assessment of risk of bias across studies (see Item 15). | 5-8 |
| Additional analysis | 23 | Give results of additional analyses, if done (e.g., sensitivity or subgroup analyses, meta-regression [see Item 16]). | NA |
| **DISCUSSION** | | | |
| Summary of evidence | 24 | Summarize the main findings including the strength of evidence for each main outcome; consider their relevance to key groups (e.g., healthcare providers, users, and policy makers). | 8-9 |
| Limitations | 25 | Discuss limitations at study and outcome level (e.g., risk of bias), and at review-level (e.g., incomplete retrieval of identified research, reporting bias). | 9 |
| Conclusions | 26 | Provide a general interpretation of the results in the context of other evidence, and implications for future research. | 9 |
| **FUNDING** | | | |
| Funding | 27 | Describe sources of funding for the systematic review and other support (e.g., supply of data); role of funders for the systematic review. | 1 |

*From:* Moher D, Liberati A, Tetzlaff J, Altman DG, The PRISMA Group (2009). Preferred Reporting Items for Systematic Reviews and Meta-Analyses: The PRISMA Statement. PLoS Med 6(6): e1000097. doi:10.1371/journal.pmed1000097

For more information, visit: **www.prisma-statement.org**.



For peer review only

For peer review only - http://bmjopen.bmj.com/site/about/guidelines.xhtml

BMJ Open

# Interactions between physicians and the pharmaceutical industry generally and sales representatives specifically and their association with physicians attitudes and prescribing habits: A systematic review

For peer review only

| | |
|---|---|
| Journal: | *BMJ Open* |
| Manuscript ID | bmjopen-2017-016408.R4 |
| Article Type: | Research |
| Date Submitted by the Author: | 20-Jul-2017 |
| Complete List of Authors: | Fickweiler, Freek; Crowd for Cure<br>Fickweiler, Ward; Crowd for Cure,<br>Urbach, Ewout; Crowd for Cure |
| <b>Primary Subject Heading</b>: | Health policy |
| Secondary Subject Heading: | Patient-centred medicine |
| Keywords: | Change management < HEALTH SERVICES ADMINISTRATION & MANAGEMENT, Health policy < HEALTH SERVICES ADMINISTRATION & MANAGEMENT, Protocols & guidelines < HEALTH SERVICES ADMINISTRATION & MANAGEMENT |
| | |

SCHOLARONE™
Manuscripts

# Interactions between physicians and the pharmaceutical industry generally and sales representatives specifically and their association with physicians attitudes and prescribing habits: A systematic review

**Fickweiler F[1], Fickweiler W[1], Urbach E[1],**

**1: Crowd for Cure, Jacob van Ruysdaelstraat 34, 9718 SG Groningen, the Netherlands**

**Corresponding author's email address: freek@crowdforcure.com**

**All authors have completed the ICMJE uniform disclosure form at http://www.icmje.org/coi_disclosure.pdf and declare: no support from any organisation for the submitted work; no financial relationships with any organisations that might have an interest in the submitted work in the previous three years, no other relationships or activities that could appear to have influenced the submitted workContributor Statement: All authors have contributed equally and have substantial contributions to the conception or design of the work; Author Freek Fickweiler for the acquisition, analysis, and interpretation of data for the work; Author Freek Fickweiler for drafting the work and Authors Ewout Urbach and Ward Fickweiler for revising it critically for important intellectual content; and all authors (Freek Fickweiler, Ward Fickweiler and Ewout Urbach) contributed to final approval of the version to be published and agreed to be accountable for all aspects of the work in ensuring that questions related to the accuracy or integrity of any part of the work are appropriately investigated and resolved.**

**Competing interest: no financial relationships with any organisations that might have an interest in the submitted work in the previous three years, no other relationships or activities that could appear to have influenced the submitted work**

**Funding: all authors declare no support from any organisation for the submitted work.**
**Data sharing statement: any data relevant to a published article will be made available alongside the article when published.**

For peer review only

**Abstract**

**Objectives**

The objective of this review is to explore interactions between physicians and the pharmaceutical industry including sales representatives and their impact on physicians' attitude and prescribing habits

**Data Sources**

Pubmed, Embase, Cochrane library and Google scholar electronic databases were searched from 1992 to August 2016 using free text words and medical subject headings relevant to the topic.

**Study Selection**

Studies included cross sectional studies, cohort studies, randomized trials and survey designs. Studies with narrative reviews, case reports, opinion polls, and letters to the editor,were excluded from data synthesis.

**Data Extraction**

Two reviewers independently extracted the data. Data on study design, study year, country, participant characteristics, setting, and number of participants were collected.

**Data Synthesis**

Pharmaceutical industry and pharmaceutical sales representative (PSR) interactions influence physicians' attitudes, their prescribing behavior and increases the number of formulary addition requests for the company's drug.

**Conclusion**

Physician-pharmaceutical industry and its sales representative's interactions and acceptance of gifts from the company's pharmaceutical sales representatives have been found to affect physicians' prescribing behavior and are likely to contribute to irrational prescribing of the company's drug. Therefore, intervention in the form of policy implementation and education about the implications of these interactions is needed.

**Strengths and limitations of the study**

- Large up-to-date systematic review of studies exploring the impact of pharmaceutical industry representative interactions on physicians

For peer review only

- This systematic review used the recommendations outlined in the Cochrane Handbook for conducting systematic reviews and the GRADE methodology to assess the quality of the evidence by outcome.
- Pubmed, Embase, Cochrane library and Google scholar electronic databases were searched from 1992, as well as grey literature.
- Most studies identified were observational and of varying methodological design
- Some studies did not provide evidence for the significance of their findings

**Keywords:** pharmaceutical sales representative; physicians, drug industry; brand prescriptions; conflict of interest ;physicians-industry interactions; pharmaceutical industry; attitude of health personnel; gifts to physicians; medical education; irrational prescriptions

For peer review only

## Introduction

The relationship between physicians and the pharmaceutical industry has evoked heated debate for many decades[1]. In 2012, the pharmaceutical industry spent $89.5 billion on physician-pharmaceutical sales representative (PSR) interactions which accounted for 60% of the global sales and marketing spending[2-8]. Previous reports have demonstrated that PSRs may influence prescribing behavior[,9-16]. However, the evidence determining whether or not pharmaceutical industry and PSRs interactions influence physicians is divided and contradictory. Studies have indicated that physicians may be unable to distinguish between promotional information and scientific evidence[17-18]. Physicians on the other hand believe their colleagues are more susceptible to pharmaceutical industry marketing strategies than themselves [19-22]. The majority of the physicians do not believe that they are affected by pharmaceutical industry and PSR interactions. Most medical and governmental institutions have installed guidelines and self-regulatory and legislative checks to regulate the relationship between physicians and the pharmaceutical industry and its representatives t[5,15,16, 23-26]. However, while administrative proposals for deregulatory reforms that would remove some governmental authority over the industry are increasing, scientific evidence rigorously examining the extent of interactions between physicians and pharmaceutical industry and it PSRs is needed. This review evaluates e critically and systemically the evidence on the impact of pharmaceutical industry and PSR interactions on physicians.

## Methodology

### *Protocol*

We followed a detailed methodology that we described in our review protocol, which is available upon request to the corresponding author. Two independent reviewers assessed selected articles as per inclusion/exclusion criteria, shortlisted them for writing the review and cross-checked their decisions about inclusion/exclusion with each other. The review followed the Preferred Reporting Items for Systematic Reviews and Meta-Analyses (PRISMA) guidelines (Appendix 1).

### *Eligibility criteria*

The eligibility criteria were:

For peer review

- Types of studies: cross sectional studies, cohort studies, randomized trials and survey designs comparing an intervention of interest on at least one facet of extent, impact on behavior and attitude.
- Types of participants: physicians, pharmaceutical representatives, physicians in training/residents. We did not consider medical students or other health professionals.
- Types of exposure: any type of interaction between physicians and the pharmaceutical industry where there is direct interaction with the physician, such as meeting with drug representatives, participating in pharmaceutical-sponsored CME event, receiving travel funding, free drug samples, industry-provided meals, gifts to the individual and active presentation of industry-related information to the physician
- Types of outcome: knowledge of physicians (e.g. accuracy of knowledge related to a specific medication), beliefs and/or attitudes of physicians regarding physician-industry interactions (e.g. perceived influence of information from the pharmaceutical company on their behavior), behavior of physicians (e.g. prescribing quantity/frequency)
- Type of control: no interaction.
- Exclusion criteria were: qualitative, ecological, econometric studies, editorials, letters to the editor, studies on medical students, small samples sizes, studies assessing non-targeted or indirect interactions (e.g. journal advertisement) and research funding.

We did not exclude studies based on risk of bias. We took risk of bias into account when grading the quality of evidence using GRADE approach.

*Search strategy*

The search strategy included Pubmed, Embase, Cochrane library and Google scholar electronic databases (January 1992 to August 2016). Databases were not searched before 1992, as these studies were already investigated in an earlier review[27]. The search combined terms for physicians and pharmaceutical, and included both free text words and medical subject heading relevant to the topic. We did not use a search filter. The supplementary information file provides the full details for one database. Additional search strategies included a search of the grey literature (theses and dissertations). Also, we reviewed the references lists of included and relevant papers.[27-29]

*Assessment of risk of bias in included studies*

Two reviewers assessed in duplicate and independently the riskof bias in each eligible study. Disagreements were resolved by discussion or adjudication by a third reviewer. We used the recommendations outlined in the Cochrane Handbook to assess the risk of bias in randomized studies. We graded each potential source of bias and rated the studies as high, low or unclear risk of bias.

*Data analysis and synthesis*

The information extracted from the selected studies included type of study, study design, type of pharmaceutical industry and PSR interaction and type of outcome. We did not conduct a meta-analysis due to the heterogeneity of study design, types of interventions, outcomes assessed, and outcome measures used. Instead, we summarized the data narratively. We assessed the quality of evidence by outcome using the GRADE methodology[30].

**Results**

We independently screened the titles and abstracts of the 2170 identified records for potential eligibility. Out of 2170, the full text of 49 eligible citations which matched the inclusion criteria were retrieved and used for qualitative assessment during the writing of the review (Figure 1, Table 1)

| Study | Authors | Country | Timeframe | Participants, setting | Study design | Interaction | Outcomes |
|---|---|---|---|---|---|---|---|
| 1 | Steinman et al., 2001 | USA | Spring 1999 | Surveys about attitudes and behaviors toward industry gifts in 105 residents at a university-based internal medicine residency program | Cross-sectional | PSR interactions, gifts | Most participants (61%) hold positive attitudes toward gifts from industry and PSR interactions and believe they do not influence their own prescribing, but only 16% believed other physicians were |

For peer review only

| | | | | | | | |
|---|---|---|---|---|---|---|---|
| | | | | | | | similarly unaffected (P< .0001) |
| 11 | De Ferrari et al., 2014 | Peru | March 2013 | Questionnaire in 155 faculty and trainee physicians of five different clinical departments working in a public general hospital | Cross-sectional | PSR interactions, medical samples, promotional material, dinners | Positive attitude towards representatives (88.5% of participants). Faculty physicians received a larger amount of medical samples and promotional material and were more prone to believe that gifts and lunches do not influence their prescribing behavior (42.2% vs. 23.6%; p=0.036) |
| 12 | Thomson et al., 1994 | New Zealand | 1991 | Questionnaire survey of 67 general practitioners | Cross-sectional | Interactions with PSR | Most general practitioners (67%) had a negative attitude toward PSR interaction |
| 13 | Kamal et al., 2015 | Egypt | July and August 2013 | Interviews with 18 physicians | Cross-sectional | Interaction with PSR | Positive attitude towards PSR interaction |
| 14 | Hodges, 1995 | Canada | October 1993-February 1994 | Survey in 105 residents of psychiatry | Cross-sectional | Interaction with PSR, drug samples, lunches | Positive attitude towards PSR interaction (56.5% of participants). The more money and |

For peer review only

| | | | | | | | |
|---|---|---|---|---|---|---|---|
| | | | | | | | promotional items a participant had received, the more likely he or she was to believe that discussions with representatives did not affect prescribing ($p < 0.05$) |
| 15 | Gibbons et al., 1998 | USA | Not reported | Survey of 392 physicians in two tertiary-care medical centers | Cross-sectional | PSR interactions,gifts, samples, travel, lunches | Positive attitude towards PSR interactions, gifts, samples and lunches |
| 16 | Spingarn et al., 1996 | USA | February 1990 | 75 internal medicine physicians in university medical center | Retrospective cohort | PSR interaction (teaching) | Attendees inappropriately prescribed PSR speakers drug compared to non-attendees ($p=0.029$) |
| 17 | Zaki, 2014 | Saudi Arabia | September-November 2013 | Survey of 250 physicians | Randomized, cross-sectional survey | Conferences, drug samples | Favorable towards promotion |
| 18 | Orlowski et al., 1994 | USA | 1987-1989 | 10 physicians that were invited for a symposium and tracking the pharmacy inventory usage reports for these drugs before and after the symposia | Cohort | Conference travel | Significant increase in the prescribing pattern of drugs occurred following the symposia ($p<0.001$) |

For peer review only

| | | | | | | | |
|---|---|---|---|---|---|---|---|
| 19 | Scheffer et al., 2014 | Brazil | 2007-2009 | Survey of 300 physicians prescribing antiretroviral drugs | Cross-sectional | Interaction with representative, drug samples, journals | Frequency of interaction; the majority of (64%) of the physicians had multiple forms of interactions with PSR |
| 20 | Brett et al., 2003 | USA | Not reported | Questionnaire of 93 physicians in a medical school | Cross-sectional | Interaction with PSR | Impact on attitudes; most physicians believed that most of PSR activities do not pose major ethical problems |
| 21 | Gupta et al., 2016 | India | June-September 2014 | Survey of 81 physicians in single hospital | Cross-sectional | Interaction with representative, drug samples, journals | Impact on prescribing; 61.7% of participants think that PSR has an impact on their prescribing (P = 0.0001) |
| 22 | Morgan et al., 2006 | USA | March 2003 | Survey of 397 obstetrician-gynaecologists | Cross-sectional | Drug samples, promotional material, lunch | Impact on prescribing, positive attitudes; most respondents thought it's proper to accept drug samples (92%), lunch (77%), an anatomical model (75%) or a well-paid consultantship (53%) from PSR |
| 23 | Alosaimi | Saudi | 2012 | Survey of | Cross- | Interaction | Positive |

For peer review only

| | | | | | | | |
|---|---|---|---|---|---|---|---|
| | et al., 2014 | Arabia | | 659 physicians | sectional | with PSR | attitude towards PSR interaction |
| 24 | Chren et al., 1994 | USA | 1989-1990 | 40 case physicians and 80 control physicians | Case control | PSR interactions, honoraria, research | Increased prescription of company's drug after PSR interaction, honoraria and research (p<0.001, all) |
| 25 | Randall et al., 2005 | USA | October 2001 | Intervention group of physicians (n=18) that received education about PSR interaction and control group (n=14) | Controlled trial | Interaction with PSR | The majority of residents found the interactions and gifts useful. Compared to the comparison group, the intervention group significantly decreased the reported number of office supplies and noneducational gifts (p<0.05) |
| 26 | Caudil et al., 1996 | USA | Not reported | Survey of 446 primary care physicians | Cross-sectional | Interaction with PSR | Significant positive correlation between physician cost of prescribing and perceived credibility, availability, applicability, and use of information provided by PSR (p < 0.01) |

For peer review only

| | | | | | | | |
|---|---|---|---|---|---|---|---|
| 27 | Andaleeb et al., 1995 | USA | Not reported | 223 physicians in northwestern Pennsylvania | Cross-sectional | Interaction with PSR | Positive attitude towards PSR interaction |
| 28 | Reeder et al., 1993 | USA | 1991-1992 | 87 residents of emergency medicine | Cross-sectional | Interaction with PSR, gifts | Most participants believed that PSR interaction had no impact on their prescribing |
| 29 | Lichstein et al., 1992 | USA | January-March 1990 | 272 directors of internal medicine residency programs | Cross-sectional | Interaction with PSR | Most participants had a positive attitude towards PSR interactions |
| 30 | Brotzman et al., 1992 | USA | Not reported | Directors of 386 family practice residency programs | Cross-sectional | Interaction with PSR | Majority of programs do not have guidelines for interaction with PSR |
| 31 | Alssageer et al., 2012 | Libya | August-October 2010 | Survey of 608 physicians in public and private practice settings | Cross-sectional | Interaction with PSR, drug samples, printed materials | Positive attitude towards PSR interactions |
| 32 | Lieb & Brandtonies, 2010 | Germany | 2007 | Survey of 208 physicians (neurology, cardiology and general medicine) | Cross-sectional | Interaction with PSR, drug samples, printed materials, lunches | Frequency and impact on attitudes |
| 33 | Lieb & Scheurich, 2014 | Germany | 2010-2011 | Survey of 160 physicians in private and public practices | Cross-sectional | Interaction with representative, drug samples, printed materials, CME | High expenditure prescribing; avoidance of industry-sponsored CME is associated with more |

For peer review only

| | | | | | | | |
|---|---|---|---|---|---|---|---|
| | | | | | | | rational prescribing habits |
| 34 | Lieb & Koch, 2013 | Germany | May-July 2012 | Survey of 1038 medical students at 8 universities | Cross-sectional | Interaction with representative, drug samples, printed materials, lunches | Most participants have contact with the pharmaceutical company; 24.6% of the participants thought gifts would influence their future prescribing behavior, while 45.1% thought gifts would influence their classmates' future prescribing behavior (p<0.001) |
| 35 | Brown et al., 2015 | USA | 2008 and 2013 | 251 directors of family medicine residency programs | Cross-sectional | Interaction with PSR, gifts, lunches | Negative attitude towards PSR interactions |
| 37 | Rahmanaer et al., 2015 | Bangladesh | December 2008-January 2009 | Survey of 83 village physicians | Cross-sectional | Interaction with PSR | Impact on their prescribing |
| 38 | Lee & Begley, 2016 | USA | 2008 | Nationally representative survey of 4720 physicians | Cross-sectional | Gifts | Gifts were associated with lower perceived quality of patient care; an inverse relationship between the frequency of received gifts and the perceived quality of care was |

For peer review only

| | | | | | | | |
|---|---|---|---|---|---|---|---|
| | | | | | | | observed |
| 39 | Montastruc et al., 2014 | France | August-October 2011 | Survey among 631 medical residents | Cross-sectional | Interaction with representative | Most participants believed that PSR interaction had no impact on their prescribing; participants who had a more positive opinion were more frequently exposed to PSR (p<0.001) |
| 40 | Ketis & Kersnik, 2013 | Slovenia | October 2011 | 895 family physicians at the primary level of care | Cross-sectional | Interaction with PSR | Positive effect on knowledge; Participants value PSRs' selling and communication skills and trustworthiness highly |
| 41 | Hurley et al., 2014 | USA | 2010 | 3500 dermatologists | Cross-sectional | Free drug samples | Impact on their prescribing; the provision of samples with a prescription by dermatologists has been increasing over time, and this increase is correlated (r = 0.92) with the use of the branded generic drugs promoted by |

For peer review only

| | | | | | | | |
|---|---|---|---|---|---|---|---|
| | | | | | | | these sample |
| 42 | Makowska, 2014 | Poland | November-December 2008 | Survey of 382 physicians | Cross-sectional | Gifts | Positive attitude towards PSR interactions |
| 43 | Siddiqui et al., 2014 | Pakistan | Not reported | Questionnaires of 352 medical students | Cross-sectional | Interaction with representative | Positive attitude towards PSR interaction |
| 55 | Workneh BD et al., 2016 | Ethiopia | February-March 2015 | Survey of 90 physicians from public and private health facilities | Cross-sectional | Interaction with representative, gifts | Positive attitude towards industry, impact on prescribing behavior; Nearly half of the physicians reported that their prescribing decisions were influenced by PSR |
| 57 | Khan N et al., 2016 | Pakistan | Not reported | Questionnaires in 472 physicians | Cross-sectional | Interaction with representative, gifts | Positive attitude towards PSR interaction |
| 58 | Saito S et al. 2010 | Japan | January-March 2008 | 1417 physicians working in internal medicine, general surgery, orthopedic surgery, pediatrics, obstetrics-gynecology, psychiatry, and ophthalmology | National Survey | Interaction with industry, receipt of gifts, funds, CME, samples | Positive attitude towards PSR and gifts, value information from PSR, interactions higher with physicians who prefer to prescribe brand names |
| 59 | Ziegler MG et al. 1995 | USA | 1993 | 27 physicians working in public and | Survey | Accuracy of information provided by PSRs about | Incorrect information often provided by |

For peer review only

| | | | | | | | |
|---|---|---|---|---|---|---|---|
| | | | | private hospitals | | drugs | speakers goes unnoticed by physicians |
| 60 | Lurie N et al., 1990 | USA | Not reported | 240 internal medicine faculty physicians in academic medical centers | Survey | Effect of interaction with PSR, free meals, honoraria and research support | Impact on prescribing behavior and formulary change requests |
| 62 | DeJong C et al., 2016 | USA | August-September 2013 | 279.669 physicians who wrote Medicare prescriptions in any of 4 drug classes: statins, cardioselective β-blockers, angiotensin-converting enzyme inhibitors and angiotensin-receptor blockers (ACE inhibitors and ARBs), and selective serotonin and serotonin-norepinephrine reuptake inhibitors (SSRIs and SNRIs) Physicians | Cross-sectional | Industry sponsored meals | Receipt of industry-sponsored meals was associated with an increased rate of brand name prescription. |
| 63 | Yeh JS et al., 2016 | USA | 2011 | All licensed Massachusetts physicians who wrote prescriptions for statins paid for | Cross-sectional | Effect of industry payment on prescription of branded drugs for cholesterol control | Payment for meals and educational programs increased prescription of brand name |



For peer review only

| | | | | | | | |
|---|---|---|---|---|---|---|---|
| | | | | under the Medicare drug benefit in 2011 (n=2444) | | | statins. |
| 65 | Bowman MA et al., 1988 | USA | Not reported | 121 physician attendees | Self report survey | Effect of CME on prescribing behavior | Sponsoring company's drugs were favored during prescription |
| 66 | Fischer MA et al., 2009 | USA | November 2006-March 2007 | Multi-disciplinary focus groups with 61 physicians | Survey | Effect of industry marketing strategies on prescription and cognitive dissonance of physicians | Most participants reported no PSR impact on their prescribing, value to have ability to evaluate information of PSRs |
| 67 | Chimonas S et al., 2007 | USA | June 2004 | Six focus groups in 32 academic and community physicians | Survey | PSR interactions | Positive attitude towards PSR interaction |
| 72 | Yeh JS et al., 2014 | USA | Not reported | 1610 US medical students | Cross-sectional | Interaction with representative, gifts, lunches | Policies separating students from representatives reduced number of interactions |
| 73 | Larkin I et al., 2014 | USA | January 2006-June 2009 | Pediatricians, child & adolescent psychiatrists in five medical centers | Survey | Interaction with PSR | Anti detailing policies reduced the prescription of off-label antidepressants and anti psychotics for children |
| 74 | Esmaily HM et al., 2010 | Iran | Not reported | 112 general physicians were randomized in two groups: 1) outcome- | Randomized trial | Effect of outcome and retinal prescribing | Rational prescribing improved in some of the important outcome-based |

For peer review only

| | | | | | | | |
|---|---|---|---|---|---|---|---|
| | | | | based educational intervention for rational prescribing and 2) concurrent CME program in the field of rational prescribing | | | indicators. No difference between two arms of the study |
| 76 | Parikh K et al., 2016 | USA | 2014 | descriptive, cross-sectional analysis of Open Payments data and 9 638 825 payments to physicians and pediatricians from January 1 to December 31, 2014 | Cross-sectional | Comparison of PSR interactions between pediatricians and other specialists; among subspecialities of pediatrics. | Pediatricians get fewer gifts from PSR than internists. There is variation among sub specialities for extent of interaction. |
| 78 | Chressanthis GA et al. 2012 | USA | Not reported | Clinical decisions of 72,114 physicians were statistically analyzed using prescription data | Survey | Effect of restricting PSRs on clinical practice and knowledge | Restricting PSRs affected information flow about drugs, both negative and positive. |

. We excluded 2000 records as they were not relevant (n = 1641), not original research (n=269), about medical students (n=4) and non-medical (e.g. ecological, econometric; n=86).

*Characteristics of included studies*

The identified studies were published between 1992 and August 2016. Most of the studies included were cross-sectional studies[1,9-13,19,21,22,31-55]. Only two studies were cohort studies[56, 57], three were randomized trials[58-60] and one study was a case-control study[61].

*Extent of interactions between physicians and the pharmaceutical industry*

We found that PSR interactions are a regular feature in the daily lives of physicians across the world [9-11,13,42,50]. Most of the attending physicians and residents have at least one interaction

For peer review only

with industry representatives per month [10,21,22,36,42]. The frequency of interactions or gifts offered and accepted varies with private versus public hospital setting and the position of the physicians in the medical hierarchy [10,13,31,38,42,43,50,58,62]. Junior residents received twice as much free drug samples from PSR interactions than senior residents [10]. PSR interactions were significantly higher at the beginning of residency[13]. The majority of program directors of internal medicine residencies in the USA allowed PSRs to meet with residents during working hours and permitted PSR sponsorship of conferences[40]. Attending physicians and physician specialists had more PSR interactions and received higher numbers of medical samples and promotional material than residents[9,42]. Participants working in private practice alone or in both sectors were more likely to receive gifts than physicians working in the public sector [38,42,50].. Most common gifts received were medical samples[9,21,22,31,36,37,42,63], promotional material[9,34,42] invitations for dinners[9], invitations for CMEs[22,34], scientific journals[34] and free lunches[21,37].

*Perspectives of physicians towards PSR interactions*

We found that physicians have a positive attitude towards PSRs[1, 13, 19, 20, 22, 31, 32, 40, 49, 58, 64] Physicians perceived PSRs as important sources of education and funding[10, 22, 32, 43, 45, 46]; while some studies reporting skeptical attitudes about the contribution of PSRs towards teaching and education[21, 36, 39, 40, 49]. Conference registration fees, informational luncheons, sponsorship of departmental journal clubs, anatomical models, and free drug samples were considered as appropriate gifts[19, 39, 51, 58]. Most of the physicians considered pharmaceutical information provided by PSRs, industry sponsored conferences and CME events as important instruments for enhancing their scientific knowledge[22, 32, 45, 46]. Compared to senior residents, significantly more junior residents felt that pharmaceutical representatives have a valuable teaching role [10].

. Most studies found that physicians do not believe that PSR interactions impact their prescribing behavoir,[1, 9-13, 65, 66], while other studies found that there was some extent of influence[21, 22, 34, 36, 37, 39, 43]. In addition, physicians considered their colleagues more susceptible than themselves to PSR marketing strategies[1, 20, 21, 37, 43]. There was a strong correlation between the amount of gifts and the belief that PSR interactions did not influence their prescribing behavior [10].

*Gifts*

For peer review only

We found that better scores on knowledge and attitudes were significantly associated with fewer interactions with representatives and their gifts[19]. Conference registration fees, informational luncheons, sponsorship of departmental journal clubs, anatomical models, and free drug samples were considered as appropriate gifts[19, 39, 51, 58.] Most of the physicians considered themselves immune to the influence of gifts[1, 10, 32, 33, 35, 37, 43, 59.] Most common gifts received were medical samples[9, 21, 22, 31, 36, 37, 42-44, 47], promotional material[9, 34, 42, 67] invitations for dinners[9] and scientific journals[34]

*Drug samples*

Most of the physicians who accepted drug samples had a positive attitude towards the pharmaceutical representatives[9, 21, 22, 31, 36, 37, 42, 43]. Accepting samples lead to higher branded drug prescription rather than generic prescribing [22,47].

*Pharmaceutical representative speakers*

Sponsored lectures/symposia of pharmaceutical companies influenced behavior of the attendees leading to the attendees prescribing more drugs from the sponsoring companies without sufficient evidence supporting superiority of those drugs[56,57]. The majority of attending physicians failed to identify inaccurate information about the company drug[18].

*Honoraria and Research Funding*

Physicians who received money to attend pharmaceutical symposia or to perform research requested formulary addition of the company's drug more often than other physicians, This association was independent of many confounding factors[61] (Table 2). Brief encounters with PSRs and receipt of honoraria or research support were predictors of faculty requested change in hospital formulary[68].

*Conference travel*

Pharmaceutical company sponsored conference travels to touristic locations have quantifiable impact on the prescribing rational of attendees. A significant increase (three times) in the prescribing rate of two company drugs was observed after the physicians attended a company sponsored symposium with all their expenses covered. Despite this significant difference in the prescribing patterns, physicians insisted there was no impact on their prescribing behaviour.[57]

For peer review only

*Industry paid lunches*

Most physicians received invitations for dinners[9] and free lunches[10, 21, 35, 43] . Clerks, interns and junior residents attended more company sponsored lunches than senior residents [10]. Pharmaceutical companies also sponsored departmental lunches during journal clubs[39]. There was no significant association between attending industry paid lunches[37] and dinners[9] and formulary request for that company's drug (Table 2).

| # | Attitudes | Prescribing behavior | Knowledge | Formulary requests | Quality of Evidence (GRADE) |
|---|---|---|---|---|---|
| **Gifts** | Receiving higher number of gifts associated with belief that PRs have no impact on their prescribing behaviour [1,14,39] | - | - | - | **Moderate** |
| **Drug samples** | Positive attitude towards the drug industry and the representatives[11,21,34] | Higher prescription of the company drug[21, 41] | - | - | **High** |
| **Pharmaceutical representative speakers** | - | Irrational prescribing [16, 18, 34] | Inability to identify false claims[16] | Increased prescription of sponsor's drug[24] | **High** |
| **Honoraria and Research Funding** | Positive attitude towards sponsor's drug [60] | - | - | Increased prescription of sponsor's drug[24] | **Low** |
| **Conference travel** | - | Significant increase in prescribing of sponsor drug[18] | - | Increased prescription of sponsor's drug[24] | **Low** |
| **Industry paid lunches** | Positive attitude towards sponsor's drug [14, 34] | Significant increase in prescribing of sponsor drug[62] | - | Increased formulary request for company drug [11,21] | **High** |
| **CME sponsorship** | Positive attitude towards sponsor's drug [24, 65] | Avoidance of industry-sponsored CME associated with more rational prescribing habits [33] | | | **Moderate** |

For peer review only

| | | | | | |
|---|---|---|---|---|---|
| **Interaction with PRS** | Positive attitude towards PSR drugs [1,11,14,58] | Higher prescription of the company drug[24] | Positive correlation between the physicians' prescribing cost and the information provided by the drug representative during the interaction[26] | Increased prescription of sponsor's drug[24] | **High** |

However, there was a significant association between attending industry paid lunches and increased prescription of branded drugs [52,53, 69].

*CME sponsorship*

Physicians who attended company sponsored CME events had more positive attitudes towards and inclination to prescribe the branded drugs[28, 34, 43, 67, 70-72]. We found that physicians who refused CME sponsorship were seen to prescribe higher proportion of generics and lower expenditure medicines when compared to physicians who attended CMEs[22].

**Discussion**

We report that there is widespread interaction between the pharmaceutical industry and physicians[9-11, 13, 42, 50]. Interactions are in the form of personal communications, free gifts such as drug samples, sponsored meals, sponsored conference travel, funding for research and CMEs and honoraria[9, 21, 22, 31, 36, 42]. The frequency of these interactions is comparable between residents and physicians[10, 21, 22, 36, 42]. However, the amount and type of gifts vary with the position of the physician in medical hierarchy, specialization and location of practice[10, 13, 31, 38, 42, 43, 50, 58, 62]. In general, trainees (residents, interns) are treated with more drug samples, stationery items and free meals than senior physicians[10,13]. Senior physicians usually avail of sponsored conferences/ trips, research funding, honoraria and CME events. The extent of these interactions varies with academic versus non-academic institutions: non-academic hospitals record more interactions than others[31, 38, 42, 50, 55]. The majority of the physicians do not believe that they are affected by PSR interactions[1, 10, 32, 33, 35, 37, 43, 59]. However, a sizeable percentage in various surveys responded in the affirmative when asked whether they thought that their peers are vulnerable[1, 20, 21, 37, 43].

For peer review only

We observe that there is a positive correlation between acceptance of gifts and physicians' urge to reciprocate favorably towards the benefactor[12,22,47, 73, 74] Considering that physicians have a social contract with society at large to provide unbiased and altruistic service, this is an alarming observation. In 2005 a joint report by the WHO and Health Action International (HAI) reported on interventions to counter promotional activities.[75] The evidence presented in that report was not eligible for our systematic review, mostly because it related to interventions on students or residents. Nevertheless, the findings suggested that interventions such as industry self-regulation and guidelines for sales representatives are not effective, while education about drug promotion might influence physician attitudes[76-78].

*Policies and educational intervention*

The relationship of physicians with patients is of a fiduciary nature. Hence activities that might affect that relationship by altering physicians' clinical behavior are not acceptable. Physician-pharmaceutical industry and PSR interactions may put the trust of patients in physicians at risk. Interaction with pharmaceutical industry and PSRs begins early in the physicians' career. Trainees are exposed to pharmaceutical industry marketing and promotional techniques from the initial years of their medical education, which impact their prescribing behavior in future. Overall, trainees, i.e., residents and interns, are more vulnerable to pharmaceutical industry and PSR interactions than senior physicians[11, 41, 62] Physicians are susceptible to pharmaceutical industry and PSR interactions, which influences their clinical decision-making leading to greater prescriptions of branded drugs over low cost generic medicines and increasing healthcare cost[22, 47, 52, 53, 69] Therefore, there is need to institute and implement stringent policies curtailing physician-pharmaceutical industry and PSR relationships, as well as educational programs to increase awareness. Previous reports have indicated that implementing policies and conducting educational programs are effective in increasing awareness of physician's attitudes towards pharmaceutical industry and PSR interactions[54, 59, 60, 76, 79-83]

*Strengths and Limitations of the study*

A major strength of this study is that is a large up-to-date systematic review of studies exploring the effects of physician and pharmaceutical industry representative interactions and residents in different settings (e.g. academic, primary care). Another strength of this study is the use of Cochrane and GRADE methodologies for conducting a review and assessing the quality of the studies. Moreover, we performed an extensive search in 3 databases and the

For peer review only

grey literature. Some of the limitations of this review are related to the included studies, as some did not provide evidence for the significance of their findings or had varying study designs and outcomes, which made it impossible to conduct a meta-analysis. Also, the included studies were subject to risk of bias related to the lack of validity of outcome measurement, and inadequate handling of significant potential confounders.

*Future implications*

Pharmaceutical industry and PSR interactions compromise the objectivity of the physicians. Educating physicians and increasing regulation of pharmaceutical industry and PSR interactions may lower the likelihood of prescribing new non-superior industry drugs and irrational prescription behavior. Further studies are required to evaluate the impact of pharmaceutical industry and PSR interactions on physicians over time and the benefits of various intervention based education programs on the clinical and ethical behavior of the physicians.

**References**

1. Steinman, M.A., M.G. Shlipak, and S.J. McPhee, *Of principles and pens: attitudes and practices of medicine housestaff toward pharmaceutical industry promotions.* The American Journal of Medicine, 2001. **110**(7): p. 551-557.
2. Wazana, A., *Physicians and the Pharmaceutical Industry.* JAMA, 2000. **283**(3): p. 373.
3. Wolfe, S.M., *Why do american drug companies spend more than $12 billion a year pushing drugs? Is it education or promotion?* Journal of General Internal Medicine, 1996. **11**(10): p. 637-639.
4. Wall, L.L. and D. Brown, *The High Cost of Free Lunch.* Obstetrics & Gynecology, 2007. **110**(1): p. 169-173.
5. Chressanthis, G.A., et al., *Determinants of pharmaceutical sales representative access limits to physicians.* Journal of Medical Marketing, 2014. **14**(4): p. 220-243.
6. International, K., *Rising costs of litigation in pharmaceutical industry.* Issues Monitor-Pharmaceuticals, 2011. **9**(14): p. 1-3.
7. MedAdNews, *Table insert on the top 50 pharma companies. Data provided by Cegedim Strategic Data.* 2010. **September**.
8. Mack J, *Pharma promotional spending in 2013.* Pharma marketing news, 2014. **13**(5).
9. De Ferrari, A., et al., *Attitudes and Relationship between Physicians and the Pharmaceutical Industry in a Public General Hospital in Lima, Peru.* PLoS ONE, 2014. **9**(6): p. e100114.
10. Hodges B. *Interactions with the pharmaceutical industry: experiences and attitudes of psychiatry residents, interns and clerks.* CMAJ: Canadian Medial Association Journal, 1995. **153**(5): p. 553.

For peer review only

11. Riese, F., et al., *Pharmaceutical industry interactions of psychiatric trainees from 20 European countries.* European Psychiatry, 2015. **30**(2): p. 284-290.
12. Lee, D. and C.E. Begley, *Physician report of industry gifts and quality of care.* Health Care Management Review, 2016. **41**(3): p. 275-283.
13. Montastruc, F., et al., *Interactions between Medical Residents and Drug Companies: A National Survey after the Mediator® Affair.* PLoS ONE, 2014. **9**(10): p. e104828.
14. Fugh-Berman, A. and S. Ahari, *Following the Script: How Drug Reps Make Friends and Influence Doctors.* PLoS Medicine, 2007. **4**(4): p. e150.
15. Kesselheim, A.S., M.M. Mello, and D.M. Studdert, *Strategies and Practices in Off-Label Marketing of Pharmaceuticals: A Retrospective Analysis of Whistleblower Complaints.* PLoS Medicine, 2011. **8**(4): p. e1000431.
16. Mulinari, S., *Unhealthy marketing of pharmaceutical products: An international public health concern.* Journal of Public Health Policy, 2016. **37**(2): p. 149-159.
17. Othman, N., et al., *Medicines information provided by pharmaceutical representatives: a comparative study in Australia and Malaysia.* BMC Public Health, 2010. **10**(1).
18. Ziegler, M.G., *The Accuracy of Drug Information From Pharmaceutical Sales Representatives.* JAMA: The Journal of the American Medical Association, 1995. **273**(16): p. 1296.
19. Alosaimi FD, A.K.A., Qadi M, Albahlal A, Alabdulkarim Y, Alabduljabbar M, Alqahtani F., *Physicians' attitudes towards interaction with the pharmaceutical industry/Attitudes des médecins par rapport á l'interaction avec l'industrie pharmaceutique.* Eastern Mediterranean Health Journal, 2014. **20**(12): p. 812.
20. Andaleeb, S.S. and R.F. Tallman, *Physician attitudes toward pharmaceutical sales representatives.* Health Care Management Review, 1995. **20**(3): p. 68-76.
21. Lieb K, B.S., *A survey of German physicians in private practice about contacts with pharmaceutical sales representatives.* Dtsch Arztebl Int., 2012. **107**(22): p. 392-8.
22. Lieb, K. and A. Scheurich, *Contact between Doctors and the Pharmaceutical Industry, Their Perceptions, and the Effects on Prescribing Habits.* PLoS ONE, 2014. **9**(10): p. e110130.
23. Francer, J., et al., *Ethical pharmaceutical promotion and communications worldwide: codes and regulations.* Philosophy, Ethics, and Humanities in Medicine, 2014. **9**(1): p. 7.
24. Canada., C.o.f.p.o.C.R.c.o.p.a.s.o., *Guide tot enhancing referrals and consultations between physicians.* College of Family Physicians of Canada., 2009.
25. Grande, D., *Limiting the Influence of Pharmaceutical Industry Gifts on Physicians: Self-Regulation or Government Intervention?* Journal of General Internal Medicine, 2009. **25**(1): p. 79-83.
26. Brennan, T.A., et al., *Health Industry Practices That Create Conflicts of Interest.* JAMA, 2006. **295**(4): p. 429.
27. Spurling, G.K., et al., *Information from Pharmaceutical Companies and the Quality, Quantity, and Cost of Physicians' Prescribing: A Systematic Review.* PLoS Medicine, 2010. **7**(10): p. e1000352.
28. J., Lexchin., *Interactions between physicians and the pharmaceutical industry: what does the literature say?* . CMAJ: Canadian Medial Association Journal, 1993. **14**(10): p. 1401-7.
29. Wazana, A., et al., *Using the Literature in Developing McGill's Guidelines for Interactions between Residents and the Pharmaceutical Industry.* Academic Medicine, 2004. **79**(11): p. 1033-1040.

For peer review only

30. Guyatt, G., et al., *GRADE guidelines: 1. Introduction—GRADE evidence profiles and summary of findings tables.* Journal of Clinical Epidemiology, 2011. **64**(4): p. 383-394.
31. Thomson AN, C.B., Barham PM., *Attitudes of general practitioners in New Zealand to pharmaceutical representatives.* Br J Gen Pract., 1994. **44**(382): p. 220-3.
32. Kamal, S., et al., *Perceptions and Attitudes of Egyptian Health Professionals and Policy-Makers towards Pharmaceutical Sales Representatives and Other Promotional Activities.* PLOS ONE, 2015. **10**(10): p. e0140457.
33. Gibbons, R.V., et al., *A comparison of physicians' and patients' attitudes toward pharmaceutical industry gifts.* Journal of General Internal Medicine, 1998. **13**(3): p. 151-154.
34. Scheffer, M.C., *Interaction between pharmaceutical companies and physicians who prescribe antiretroviral drugs for treating AIDS.* Sao Paulo Medical Journal, 2014. **132**(1): p. 55-60.
35. Brett, A.S., W. Burr, and J. Moloo, *Are Gifts From Pharmaceutical Companies Ethically Problematic?* Archives of Internal Medicine, 2003. **163**(18): p. 2213.
36. Gupta, S., R. Nayak, and R. Sivaranjani, *A study on the interactions of doctors with medical representatives of pharmaceutical companies in a Tertiary Care Teaching Hospital of South India.* Journal of Pharmacy and Bioallied Sciences, 2016. **8**(1): p. 47.
37. Morgan, M.A., *Interactions of doctors with the pharmaceutical industry.* Journal of Medical Ethics, 2006. **32**(10): p. 559-563.
38. Caudill, T.S., *Physicians, pharmaceutical sales representatives, and the cost of prescribing.* Archives of Family Medicine, 1996. **5**(4): p. 201-206.
39. Reeder, M., J. Dougherty, and L.J. White, *Pharmaceutical representatives and emergency medicine residents: A national survey.* Annals of Emergency Medicine, 1993. **22**(10): p. 1593-1596.
40. Lichstein PR, T.R., O'Brien K., *Impact of pharmaceutical company representatives on internal medicine residency programs: a survey of residency program directors.* Archives of Internal Medicine, 1992. **152**(5): p. 1009-13.
41. Brotzman GL, M.D., *Policies regulating the activities of pharmaceutical representatives in residency programs.* Journal of Family Practice, 1992. **34**(1): p. 54-8.
42. Alssageer, M. and S. Kowalski, *A survey of pharmaceutical company representative interactions with doctors in Libya.* Libyan Journal of Medicine, 2012. **7**(1): p. 18556.
43. Lieb K, K.C., *Medical students' attitudes to and contact with the pharmaceutical industry: a survey at eight German University hopitals.* Dtsch. Arztebl. Int., 2013. **110**(35): p. 584-90.
44. Brown SR, E.D., Berman AF., *Pharmaceutical industry interactions in family medicine residencies decreased between 2008 and 2013: a CERA study.* Fam. Med., 2015. **47**(4): p. 279-82.
45. Rahmana MH, A.S., Tuddenhama S, Petoa H, Iqbalb M, Bhuiyab A, Peters DH., *What do they do? Interactions between village doctors and medical representatives in Chakaria, Banladesh.* Int. Health., 2015. **7**(1): p. 266-71.
46. KlemencKetis, Z. and J. Kersnik, *Which Pharmaceutical Sales Representatives' Features do Slovenian Family Physicians Value?* Acta Informatica Medica, 2013. **21**(4): p. 257.
47. Hurley, M.P., R.S. Stafford, and A.T. Lane, *Characterizing the Relationship Between Free Drug Samples and Prescription Patterns for Acne Vulgaris and Rosacea.* JAMA Dermatology, 2014. **150**(5): p. 487.

For peer review only

48. Makowska, M., *Interactions between Doctors and Pharmaceutical Sales Representatives in a Former Communist Country.* Cambridge Quarterly of Healthcare Ethics, 2014. **23**(03): p. 349-355.
49. Siddiqui, U.T., et al., *Attitudes of medical students towards incentives offered by pharmaceutical companies- perspective from a developing nation- a cross sectional study.* BMC Medical Ethics, 2014. **15**(1).
50. Workneh, B.D., et al., *Influence of Medical Representatives on Prescribing Practices in Mekelle, Northern Ethiopia.* PLOS ONE, 2016. **11**(6): p. e0156795.
51. Khan, N., et al., *Perceptions and Attitudes of Medical Sales Representatives (MSRs) and Prescribers Regarding Pharmaceutical Sales Promotion and Prescribing Practices in Pakistan.* Journal of Young Pharmacists, 2016. **8**(3): p. 244-250.
52. DeJong, C., et al., *Pharmaceutical Industry–Sponsored Meals and Physician Prescribing Patterns for Medicare Beneficiaries.* JAMA Internal Medicine, 2016. **176**(8): p. 1114.
53. Yeh, J.S., et al., *Association of Industry Payments to Physicians With the Prescribing of Brand-name Statins in Massachusetts.* JAMA Internal Medicine, 2016. **176**(6): p. 763.
54. Yeh, J.S., et al., *Association of Medical Students' Reports of Interactions with the Pharmaceutical and Medical Device Industries and Medical School Policies and Characteristics: A Cross-Sectional Study.* PLoS Medicine, 2014. **11**(10): p. e1001743.
55. Parikh, K., W. Fleischman, and S. Agrawal, *Industry Relationships With Pediatricians: Findings From the Open Payments Sunshine Act.* PEDIATRICS, 2016. **137**(6): p. e20154440-e20154440.
56. Spingarn, R.W., J.A. Berlin, and B.L. Strom, *When pharmaceutical manufacturers' employees present grand rounds, what do residents remember?* Academic Medicine, 1996. **71**(1): p. 86-8.
57. Orlowski, J.P. and L. Wateska, *The Effects of Pharmaceutical Firm Enticements on Physician Prescribing Patterns: there's no such thing as a free lunch.* Chest, 1992. **102**(1): p. 270-273.
58. Zaki, N.M., *Pharmacists' and physicians' perception and exposure to drug promotion: A Saudi study.* Saudi Pharmaceutical Journal, 2014. **22**(6): p. 528-536.
59. Randall, M., Rosenbaum JR, Rohrbaugh RM, Rosenheck RA., *Attitudes and Behaviors of Psychiatry Residents Toward Pharmaceutical Representatives Before and After an Educational Intervention.* Academic Psychiatry, 2005. **29**(1): p. 33-39.
60. Esmaily, H.M., et al., *Can rational prescribing be improved by an outcome-based educational approach? A randomized trial completed in Iran.* Journal of Continuing Education in the Health Professions, 2010. **30**(1): p. 11-18.
61. Chren MM, L.C., *Physicians' Behavior and Their Interactions With Drug Companies: a controlled study of physicians who requested additions to a hospital drug formulary.* JAMA, 1994. **271**(9): p. 684.
62. Zipkin, D.A. and M.A. Steinman, *Interactions between pharmaceutical representatives and doctors in training.* Journal of General Internal Medicine, 2005. **20**(8): p. 777-786.
63. McMurray, R.J., *Gifts to Physicians From Industry.* JAMA: The Journal of the American Medical Association, 1991. **265**(4): p. 501.
64. Lotfi, T., et al., *Knowledge, beliefs and attitudes of physicians in low and middle-income countries regarding interacting with pharmaceutical companies: a systematic review.* BMC Health Services Research, 2016. **16**(1).
65. Fischer, M.A., et al., *Prescribers and Pharmaceutical Representatives: Why Are We Still Meeting?* Journal of General Internal Medicine, 2009. **24**(7): p. 795-801.

For peer review only

66. Chimonas, S., T.A. Brennan, and D.J. Rothman, *Physicians and Drug Representatives: Exploring the Dynamics of the Relationship.* Journal of General Internal Medicine, 2007. **22**(2): p. 184-190.
67. Saito, S., K. Mukohara, and S. Bito, *Japanese Practicing Physicians' Relationships with Pharmaceutical Representatives: A National Survey.* PLoS ONE, 2010. **5**(8): p. e12193.
68. Lurie, N., et al., *Pharmaceutical representatives in academic medical centers.* Journal of General Internal Medicine, 1990. **5**(3): p. 240-243.
69. Steinbrook, R., *Industry Payments to Physicians and Prescribing of Brand-name Drugs.* JAMA Internal Medicine, 2016. **176**(8): p. 1123.
70. Bowman, M.A. and D.L. Pearle, *Changes in drug prescribing patterns related to commercial company funding of continuing medical education.* Journal of Continuing Education in the Health Professions, 1988. **8**(1): p. 13-20.
71. Grundy Q, B.L., Malone R 2013, *Interactions between non-physician clinicians and industry: a systematic review.* PLoS Med. , 2013. **11**(Nov 26).
72. Dixon, D., et al., *Controlling Quality in CME/CPD by Measuring and Illuminating Bias.* Journal of Continuing Education in the Health Professions, 2011. **31**(2): p. 109-116.
73. Silverman, E., *Everything you need to know about the Sunshine Act.* BMJ, 2013. **347**(jul26 1): p. f4704-f4704.
74. Shalowitz, D.I., M.A. Spillman, and M.A. Morgan, *Interactions with industry under the Sunshine Act: an example from gynecologic oncology.* American Journal of Obstetrics and Gynecology, 2016. **214**(6): p. 703-707.
75. Norris P, H.A., Lexchin J, et al., *Drug promotion—what we know, what we have yet to learn—reviews of materials in the WHO/HAI database on drug promotion.* EDM Research Series, 2005(No. 032.).
76. Carroll, A.E., et al., *To What Extent Do Educational Interventions Impact Medical Trainees' Attitudes and Behaviors Regarding Industry-Trainee and Industry-Physician Relationships?* PEDIATRICS, 2007. **120**(6): p. e1528-e1535.
77. Birkhahn, R.H., et al., *A Review of the Federal Guidelines That Inform and Influence Relationships Between Physicians and Industry.* Academic Emergency Medicine, 2009. **16**(8): p. 776-781.
78. Chressanthis, G.A., et al., *Can Access Limits on Sales Representatives to Physicians Affect Clinical Prescription Decisions? A Study of Recent Events With Diabetes and Lipid Drugs.* The Journal of Clinical Hypertension, 2012. **14**(7): p. 435-446.
79. Sergeant MD, H.P., Godwin M, Walker DM, McHenry P., *Interactions with the pharmaceutical industry: a survey of family medicine residents in Ontario.* CMAJ: Canadian Medial Association Journal, 1996. **155**(9): p. 1243.
80. T Montague, B., A.H. Fortin Vi, and J. Rosenbaum, *A systematic review of curricula on relationships between residents and the pharmaceutical industry.* Medical Education, 2008. **42**(3): p. 301-308.
81. Larkin, I., et al., *Restrictions On Pharmaceutical Detailing Reduced Off-Label Prescribing Of Antidepressants And Antipsychotics In Children.* Health Affairs, 2014. **33**(6): p. 1014-1023.
82. R., P., *Guidelines for faculty involvement in commercially supported continuing medical-education.* Academic Medicine, 1992.
83. Greenland, P., *Time for the Medical Profession to Act.* Archives of Internal Medicine, 2009. **169**(9): p. 829.

**Legends**

For peer review only

**Figure 1:** PRISMA flow diagram showing search strategy and included studies

**Table 1:** Characteristics of included studies

**Table 2:** Impact of physician-pharmaceutical industry interaction on physician

**Supplementary information file:** Search strategy

**The Corresponding Author has the right to grant on behalf of all authors and does grant on behalf of all authors, a worldwide licence (http://www.bmj.com/sites/default/files/BMJ%20Author%20Licence%20March%202013.doc) to the Publishers and its licensees in perpetuity, in all forms, formats and media (whether known now or created in the future), to i) publish, reproduce, distribute, display and store the Contribution, ii) translate the Contribution into other languages, create adaptations, reprints, include within collections and create summaries, extracts and/or, abstracts of the Contribution and convert or allow conversion into any format including without limitation audio, iii) create any other derivative work(s) based in whole or part on the on the Contribution, iv) to exploit all subsidiary rights to exploit all subsidiary rights that currently exist or as may exist in the future in the Contribution, v) the inclusion of electronic links from the Contribution to third party material where-ever it may be located; and, vi) licence any third party to do any or all of the above. All research articles will be made available on an Open Access basis (with authors being asked to pay an open access fee—see http://www.bmj.com/about-bmj/resources-authors/forms-policies-and-checklists/copyright-open-access-and-permission-reuse).**
**The terms of such Open Access shall be governed by a Creative Commons licence—details as to which Creative Commons licence will apply to the research article are set out in our worldwide licence referred to above.**

For peer review only

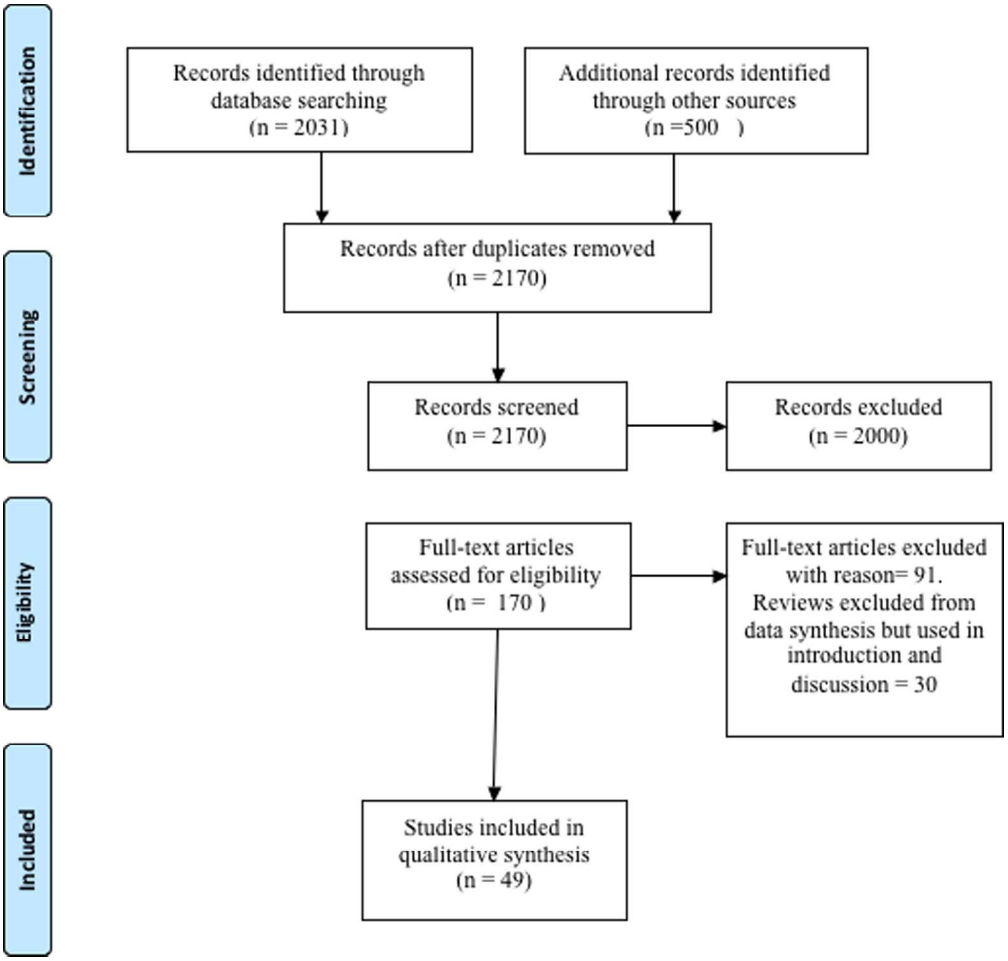


Flow diagram of study selection.

only

# PRISMA 2009 Checklist

| Section/topic | # | Checklist item | Reported on page # |
|---|---|---|---|
| **TITLE** | | | |
| Title | 1 | Identify the report as a systematic review, meta-analysis, or both. | 1 |
| **ABSTRACT** | | | |
| Structured summary | 2 | Provide a structured summary including, as applicable: background; objectives; data sources; study eligibility criteria, participants, and interventions; study appraisal and synthesis methods; results; limitations; conclusions and implications of key findings; systematic review registration number. | 2 |
| **INTRODUCTION** | | | |
| Rationale | 3 | Describe the rationale for the review in the context of what is already known. | 3 |
| Objectives | 4 | Provide an explicit statement of questions being addressed with reference to participants, interventions, comparisons, outcomes, and study design (PICOS). | 3 |
| **METHODS** | | | |
| Protocol and registration | 5 | Indicate if a review protocol exists, if and where it can be accessed (e.g., Web address), and, if available, provide registration information including registration number. | 4 |
| Eligibility criteria | 6 | Specify study characteristics (e.g., PICOS, length of follow-up) and report characteristics (e.g., years considered, language, publication status) used as criteria for eligibility, giving rationale. | 3 |
| Information sources | 7 | Describe all information sources (e.g., databases with dates of coverage, contact with study authors to identify additional studies) in the search and date last searched. | 4 |
| Search | 8 | Present full electronic search strategy for at least one database, including any limits used, such that it could be repeated. | 4 |
| Study selection | 9 | State the process for selecting studies (i.e., screening, eligibility, included in systematic review, and, if applicable, included in the meta-analysis). | 3/4 |
| Data collection process | 10 | Describe method of data extraction from reports (e.g., piloted forms, independently, in duplicate) and any processes for obtaining and confirming data from investigators. | 4 |
| Data items | 11 | List and define all variables for which data were sought (e.g., PICOS, funding sources) and any assumptions and simplifications made. | 4 |
| Risk of bias in individual studies | 12 | Describe methods used for assessing risk of bias of individual studies (including specification of whether this was done at the study or outcome level), and how this information is to be used in any data synthesis. | 3 |
| Summary measures | 13 | State the principal summary measures (e.g., risk ratio, difference in means). | 3 |
| Synthesis of results | 14 | Describe the methods of handling data and combining results of studies, if done, including measures of consistency (e.g., $I^2$) for each meta-analysis. | 3 |

For peer review only - http://bmjopen.bmj.com/site/about/guidelines.xhtml

# PRISMA 2009 Checklist

| Section/topic | # | Checklist item | Reported on page # |
|---|---|---|---|
| Risk of bias across studies | 15 | Specify any assessment of risk of bias that may affect the cumulative evidence (e.g., publication bias, selective reporting within studies). | 3 |
| Additional analyses | 16 | Describe methods of additional analyses (e.g., sensitivity or subgroup analyses, meta-regression), if done, indicating which were pre-specified. | 3 |
| **RESULTS** | | | |
| Study selection | 17 | Give numbers of studies screened, assessed for eligibility, and included in the review, with reasons for exclusions at each stage, ideally with a flow diagram. | 5 |
| Study characteristics | 18 | For each study, present characteristics for which data were extracted (e.g., study size, PICOS, follow-up period) and provide the citations. | 5-8 |
| Risk of bias within studies | 19 | Present data on risk of bias of each study and, if available, any outcome level assessment (see item 12). | 5-8 |
| Results of individual studies | 20 | For all outcomes considered (benefits or harms), present, for each study: (a) simple summary data for each intervention group (b) effect estimates and confidence intervals, ideally with a forest plot. | 5-8 |
| Synthesis of results | 21 | Present results of each meta-analysis done, including confidence intervals and measures of consistency. | NA |
| Risk of bias across studies | 22 | Present results of any assessment of risk of bias across studies (see Item 15). | 5-8 |
| Additional analysis | 23 | Give results of additional analyses, if done (e.g., sensitivity or subgroup analyses, meta-regression [see Item 16]). | NA |
| **DISCUSSION** | | | |
| Summary of evidence | 24 | Summarize the main findings including the strength of evidence for each main outcome; consider their relevance to key groups (e.g., healthcare providers, users, and policy makers). | 8-9 |
| Limitations | 25 | Discuss limitations at study and outcome level (e.g., risk of bias), and at review-level (e.g., incomplete retrieval of identified research, reporting bias). | 9 |
| Conclusions | 26 | Provide a general interpretation of the results in the context of other evidence, and implications for future research. | 9 |
| **FUNDING** | | | |
| Funding | 27 | Describe sources of funding for the systematic review and other support (e.g., supply of data); role of funders for the systematic review. | 1 |

*From:* Moher D, Liberati A, Tetzlaff J, Altman DG, The PRISMA Group (2009). Preferred Reporting Items for Systematic Reviews and Meta-Analyses: The PRISMA Statement. PLoS Med 6(6): e1000097. doi:10.1371/journal.pmed1000097

For more information, visit: **www.prisma-statement.org**.



For peer review only - http://bmjopen.bmj.com/site/about/guidelines.xhtml

Search strategy for PubMed search engine of Medline

In an attempt to find all related literature on the topic, studies related to physician-pharmaceutical representative interactions that affect the prescribing behavior of the physicians were identified through computerized searches using, but not limited to, the following subject headings and text words in PubMed from 1992 to 2016.

1. Physician interactions with pharmaceutical industry
2. Physician attitude towards pharmaceutical representatives
3. Behavior of physicians towards pharmaceutical representatives
4. Gifts AND physician AND pharmaceutical representatives
5. Honoraria AND physician AND pharmaceutical representatives
6. Continuing medical education AND physician AND pharmaceutical representatives
7. Research funding AND physician AND pharmaceutical representatives
8. Conference travel AND physician AND pharmaceutical representatives
9. Industry sponsored meals AND physician behavior

For peer review only
